# Climate change, hunger and rural health through the lens of farming styles: An agent-based model to assess the potential role of peasant farming

**DOI:** 10.1371/journal.pone.0246788

**Published:** 2021-02-11

**Authors:** Simon J. Lloyd, Zaid Chalabi

**Affiliations:** 1 Faculty of Public Health and Policy, Department of Public Health, Environments and Society, London School of Hygiene and Tropical Medicine, London, United Kingdom; 2 UCL Institute for Environmental Design and Engineering, Bartlett School of Environment, Energy and Resources, University College London, London, United Kingdom; Universidad Nacional Autonoma de Nicaragua Leon, NICARAGUA

## Abstract

Undernutrition is a major contributor to the global-burden of disease, and global-level health impact models suggest that climate change-mediated reductions in food quantity and quality will negatively affect it. These models, however, capture just some of the processes that will shape future nutrition. We adopt an alternative standpoint, developing an agent-based model in which producer-consumer smallholders practice different ‘styles of farming’ in the global food system. The model represents a hypothetical rural community in which ‘orphan’ (subsistence) farmers may develop by adopting an ‘entrepreneurial’ style (highly market-dependent) or by maintaining a ‘peasant’ style (agroecology). We take a first look at the question: how might patterns of farming styles—under various style preference, climate, policy, and price transmission scenarios—impact on hunger and health-supporting conditions (incomes, work, inequality, ‘real land productivity’) in rural areas? imulations without climate change or agricultural policy found that style preference patterns influence production, food price, and incomes, and there were trade-offs between them. For instance, entrepreneurial-oriented futures had the highest production and lowest prices but were simultaneously those in which farms tended towards crisis. Simulations with climate change and agricultural policy found that peasant-orientated agroecology futures had the highest production, prices equal to or lower than those under entrepreneurial-oriented futures, and better supported rural health. There were, however, contradictory effects on nutrition, with benefits and harms for different groups. Collectively the findings suggest that when attempting to understand how climate change may impact on future nutrition and health, patterns of farming styles—along with the fates of the households that practice them—matter. These issues, including the potential role of peasant farming, have been neglected in previous global-level climate-nutrition modelling but go to the heart of current debates on the future of farming: thus, they should be given more prominence in future work.

## Introduction

Hunger and undernutrition are major contributors to the global burden of disease and have proven difficult to eliminate despite long being the focus of global programmes [1–3]. For instance, current estimates suggest 820 million people are undernourished (insufficient calorie intake) and 149 million children aged under 5 are stunted (low height-for-age) [4]. Part of the reason for this seeming intractability is the complexity of its causation, involving factors and processes operating at the individual- and population-level in multiple spheres, ranging from infectious diseases [5], to education [6], to civil conflict [7], to foreign direct investment [8]. While climate and weather have always played a role in hunger, ongoing climate change is increasing this complexity and is likely to further impede actions to eradicate it [9].

Global-level climate-health impact models have repeatedly found that population undernourishment, child undernutrition (e.g. stunting), and dietary quality will be negatively affected by climate change-mediated changes in food production [e.g. 10,11–20]. Ultimately, whether or not an individual is poorly nourished is determined by the quantity and quality of food they can access, as well as whether they are affected by infectious diseases that compromise nutrient absorption [21,22], and this is reflected in both the method and theory underlying extant climate-nutrition models [10–20]. Methodologically (and put in general terms), climate impacts on crop production are assessed in order to estimate changes in food quantity and quality, and this is in turn used to assess expected dietary changes in consumers. Socioeconomic conditions associated with nutritional status (for example, water, sanitation, and female access to education [6]) are also typically accounted for as modifying factors, albeit usually crudely represented as exogenously specified (i.e. not modelled or affected by climate change) Gross Domestic Product per capita (GDPpc). In terms of theory (which is often implicit), these approaches tend to see the dominant cause of poor nutrition as food scarcity (in terms of quantity or quality), which may arise from an absolute lack of food or its unaffordability. This is a crucial perspective given expected population growth and the threat climate change poses to food production.

The complexity of undernutrition suggests, however, that previous climate-health impact modelling captures just some of the processes that are likely to shape future nutrition. In fact, despite the persistence of undernutrition, there is currently more than enough food produced globally to feed everyone [23]. No single model could be expected to represent all the important processes but it would be useful to develop models that adopt perspectives in addition to that of total food production. Illustrating this, recent global-level modelling found that ensuring decent incomes for farmers may be a key means of reducing future undernutrition and vulnerability to climate change, although farming households were not directly represented in the model [24].

In this paper, we develop a model that takes an alternative perspective on the climate-undernutrition relation. In doing so, we move from a view centred on food quantity and quality—or, put another way, nutrients and human physiology—to one more grounded in the ‘social determinants of health’ (e.g. the causes of a lack of food in population groups despite overall abundance) [25] and the ‘political economy of health’ (e.g. how policy choices shape social conditions and the viability of different farming options) [26]. This potentially opens multiple lines of inquiry, but we developed our particular perspective based on the following.

Firstly, ‘half the world’s undernourished people and the majority of people living in absolute poverty’ are found amongst the 2 billion producer-consumers living on smallholder farms [27]. At the same time, it has been argued this same group could hold the key to feeding populations healthily, mitigating climate change (and other environmental damages), and providing decent rural livelihoods [28–30]. Yet, producer-consumers—who comprise around one third of world population—are not explicitly included in existing global-level climate-undernutrition models which separate production and consumption by design (We refer to farmers as producer-consumers in this paper to highlight that we are re-uniting production and consumption).

Secondly, when representing production, existing global-level climate-health impact models allow for between-farm quantitative differences (e.g. farm size, input use) [e.g. 10,11–20,31] but do not qualitatively distinguish ‘farming styles’ [32]. Literature on both historical [e.g. 32,33] and future farming [e.g. 30,34], however, has highlighted non-trivial between-farm distinctions and the influence this has on food production and hunger.

Both these aspects, i.e. producer-consumer households and farm typologies, are a central focus of the long-established tradition of ‘farm household modelling’, which has specifically considered questions about food security under climate change [e.g. 33–37]. Our perspective has many similarities to this tradition but differs to previous work in three important ways.

Firstly, we focus on ‘styles of farming’. When assessing farm heterogeneity, farm household modelling has tended to use empirically-derived typologies based on site-specific structural and functional features [e.g. 34–38]. In contrast, styles of farming arise from empirically-derived theory based on observations in a range of locations and aim to identify ‘patterns of coherence underlying this heterogeneity’ [39]. Thus, farming styles are more generalizable and potentially applicable when considering multiple sites in global-level studies. Two key dimensions of styles are (i) production, particularly whether intensification is labour- or technology-driven, and (ii) reproduction, which may be largely via on-farm generated inputs or the mobilization of external resources. The actual expressions of style differ by location and over time but the basic ordering principles remain largely consistent [32,39]. The central purpose of our model is to investigate how pattens of farming styles may influence hunger and rural health under climate change.

Secondly, because we focus on styles, we implicitly adopt a perspective that is more aligned to food sovereignty (which includes aspects of how food is produced and who controls it) than to food security (which is concerned with food availability, access, utilization, and stability) [40]. Thirdly, the key purpose of our model is to draw the attention of the climate-health impact community to the health-related implications of the neglected issue of farming styles, rather than make a detailed assessment in a specific farming community using state-of-the-art methods.

In this paper, we develop an agent-based model (ABM) in which the agents are part of a community of producer-consumer smallholders practicing different styles of farming in the global food system. We use the model to take a first look at the question: *how might farm development trajectories—under various farming-style preference*, *climate*, *policy*, *and price transmission scenarios—impact on hunger and health-related conditions in rural areas*? That is, in contrast to previous climate-health impact modelling that traces a pathway from climate change to hunger (for instance, see Fig 2 in [41]), we begin by assessing how patterns of farming styles may impact on rural health (in the absence of climate change), and then assess how climate change may modify this relation. Our model is intended to be a ‘proof of concept’ model and is set in a hypothetical farming community.

This paper has three main purposes: (i) to familiarize the climate-health impact community (and other interested groups) with the concept of ‘styles of farming’, particularly in terms of inseparable ideas about who is farming (‘peasants’ vs ‘entrepreneurs’ [32,42]; definitions ahead) and how they are farming (agroecology vs reliance on purchased inputs [28,43]); (ii) to use patterns in the model outputs to draw attention to the role different farming futures may play in shaping population health via both food- and non-food-related processes, and the implications of climate change; and (iii) stimulate debate about the importance of these largely neglected (at least in climate-health impact modelling) issues and spur the development of more detailed models, including by drawing on approaches used in farm household modelling.

The next section gives an overview of the ABM. Following this, results from a set of simulation experiments conducted under various scenarios are presented and discussed. We finish with some concluding remarks on the implications for future research.

## Methods

ABMs are simulation models which represent agents, their goal-orientated decisions, the actions they take, and their interactions with other agents and the environment (understood in broad terms) [44]. They track how micro-level actions unfold over time to give rise to macro-level patterns. While ABMs have been used to study various aspects of population health [for a recent review see 45] as well as agricultural systems, climate change and food security [e.g. 33,34,46,47], to our knowledge ABM has not been previously used to assess the potential influence of styles of farming on the relation between climate change on health.

**Fig 1:**
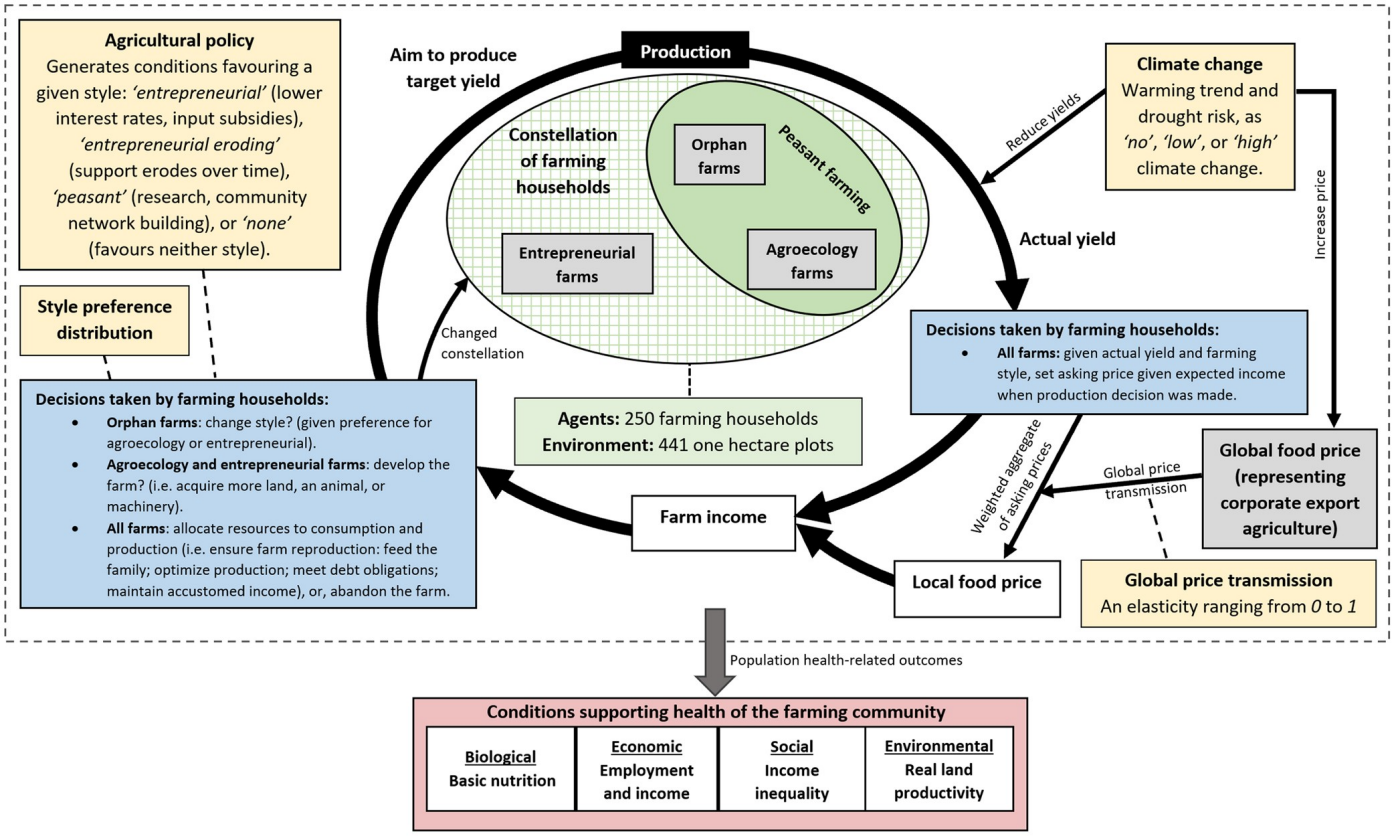
Schematic diagram of the agent-based model. The central cycle (thick black arrows) represents the farm production process, with each cycle occurring over one year (i.e. one timestep). Agents (farming households), corporate agriculture (represented as an exogenous forcing term) and the environment (1ha plots) are shown in grey and green. Agent decisions are shown in blue. Scenario options are shown in orange. Health-related outcomes are shown in red. See text for further details.

Existing global-level climate undernutrition models typically link together a chain of component models [For an example, see Fig 1 in 31]. In this approach, the health component model is generally driven by macro-to-macro statistical correlations (for instance, the correlation between ‘total quantity of food’ and ‘proportion at risk of stunting’), and the crop production component model generally assumes homogeneity of farmer goals [e.g. 10,11–17,31]. That is, health component model operates entirely at an aggregate level, where the latter (partly) arises from essentially homogenous farming-related behaviours at a lower level. We argue this is a critical limitation given both the contested nature of how farming futures could or should look [30,48] and previous findings of farm household models [e.g. 34,36,38]; what happens at the micro-level matters for population health.

ABMs overcome the above limitations. In our case, ABM allows an assessment of how changing patterns of farming styles, which manifest as heterogenous agent behaviour at the micro-level, interact to give rise to macro-level conditions (such as aggregate food price) which in turn (via feedback), along with other structural conditions (such as climate, agricultural policy), influence micro-level behaviour, giving rise to farm development trajectories and patterns of health-related conditions [cf. 42,49]. Our model, however, introduces a new set of limitations (see ‘Discussion‘). Thus, our approach should be seen as offering insights that are complimentary to those gained from previous climate-health impact modelling and farm household modelling, as well as providing guidance on the development of future models.

### Overview of the model and simulations

Our ABM represents a hypothetical world in which a population of peasant producer-consumer farming households practicing ‘orphan’ farming (i.e. subsistence farming) on one hectare plots may develop by adopting an entrepreneurial farming style which is highly dependent on purchased inputs, or, by maintaining a peasant style but adopting agroecology, which is a way of farming that is driven by enhancing and utilizing on-farm ecological processes [32,50,51] (Fig 1). This occurs under scenarios which vary by (i) the proportion of farmers preferring a given style of farming, (ii) the style favoured by agricultural policy, (iii) the degree of influence of global food prices on local prices (as an indicator of globalization of the food system), and (iv) the severity of climate change. Simulations are run in annual time steps for 50 years and, amongst other things, five health-supporting outcomes from different spheres are assessed: basic nutrition (biological); farm incomes and labour (economic); income inequality (social); and, ‘real land productivity’ (a measure of farming intensity; environmental). Fig 1 shows a schematic of the model.

We developed our agent types based on the work of van der Ploeg [32,42,48], and for theoretical consistency we drew on the same body of work to model their style-specific economic behaviours. We further describe our approach ahead (see ‘Model process’).

Tables 1 and 2 describe the key model variables and parameters. In general, we parameterized the model using approximations based on the literature. For instance, we derived approximate rates of temperature rise in our climate change scenarios based on averages in the Representative Concentration Pathways (RCPs; these are the scenarios currently used in climate change impact assessments) [52]; we estimated yield loss per degree of warming based on existing quantifications [53,54]; we used ‘rules of thumb’ for productivity and consumption in subsistence farming (Mazoyer and Roudart, 2006)); and, we estimated annual yield increments in peasant agriculture based on qualitive knowledge (van der Ploeg, 2013)). We took this approach because: (i) it allowed for simplicity and transparency; (ii) the model represents a hypothetical rural area; (iii) quantitative estimates for some parameters were not available; and, (iv) patterns in the results rather than quantifications are of central interest (We discuss this further in the ‘Model limitations’ section of the ‘Discussion‘).

**Table 1:** Key environment and agent factors, their initial values, and how they change over time^a^.

Factor (units)	Function or effect	Initial value	Change over time	Notes
**Landscape**				
‘Local area’ (ha)	Grid of 1ha plots.	441 1ha plots.	No change.	A 21 by 21 grid of arable plots.
Plot max productivity (kg/year)	Each plot has a maximum productivity under orphan agriculture (i.e. in which no non-labour farm inputs are used).	Randomly set for each plot: 1000kg/year ±20% (uniform distribution). [Based on [50]]	Slow rise on optimized peasant farms. ‘Peasant’ policy: orphan 1.5%/year, Agroecology 3% per year; other policies: Orphan 1%/year, Agroecology 1.5%/year. Max productivity = max productivity for entrepreneurial farmers. [Based on [42,55]]	‘Optimized’ in terms of production; assumed that if farmer unable to optimize, then also unable to gain production increases. Assumes no land degradation under any style.
Plot agroecology yield multiple (scalar)	Max productivity of a plot is raised by a given multiple after transitioning to agroecology.	Randomly set for each plot: mean = 4, SD = 1.5 (normal distribution, restricted to values between 2 and 7). [Based on [51,56,57]]	No change.	Productivity rises slowly during the transition phase, with the full yield multiple being achieved after the agroecology transition period.
Agroecology transition period (years)	Number of years to transition a plot to agroecology.	3 years. [Based on [51]]	No change.	Transition achieved via labour intensification.
**Agents**				
Farming households (number)	Farming households, each of four people, practicing a particular style of farming. Using manual tools, each household can farm one hectare.	250; each randomly assigned a 1ha plot; all practicing orphan agriculture; preference to develop via a particular style distributed according to scenario.	Households change to preferred style if they have access to sufficient resources, or, abandon farming if nutrition falls below 50% of a basic diet.	Initially ~40% of plots are unoccupied. Approximates conditions in lower income countries. [50,58,59]
Family basic diet (kg of cereal/year)	Quantity of cereal equivalents providing a basic diet to a family for one year.	700kg/year (equiv. to ~2200kcal/person/day). [Based on [50]]	No change. Households abandon their farm if they are unable to obtain 50% of a basic diet.	Household members do not age over time.
Labour diet (kcal/day)	Worker calorie intake/day to allow a given amount of labour power.	5100kcal/day for max production on 1ha; diminishing returns as intake increases to this level. [Based on [60,61]]	Acquiring working animals or a small tractor allows a worker to farm more than 1ha (Table 2). Labour input requirements double under agroecology.	For orphan agriculture, max production on 1ha with manual tools requires 150 ten hour labour days/year. [Based on [60]]
Agroecology labour multiple (scalar)	Increase in labour requirements for maximum production in agroecology.	2 (i.e. for max production, required labour time doubles). [Based on [51]]	No change.	‘Necessary input’ requirements rise proportionally with labour (see Table 2).
**Climate**				
Warming trend and yield losses (degrees/year, and, % loss/degree of warming)	Yields decline as warming increases, with lower losses for agroecology. (For effects on global food price, see Table 2.)	Warming = 0. Yield loss = 4%/degree of warming [Based on [53,54]]; losses reduced by 10% under agroecology. [Based on [51]]	Linear rise in warming. High CC: 2 degrees/50 years; Low CC: 1 degree/50 years; No CC: no warming. [Based on [62]]	An approximation guided by average warming under the Representative Concentration Pathways [52]. Agroecology loss reductions are an approximation.
Drought risk and yield losses (annual risk, and, % loss/event)	Proportion of yield lost if a drought occurs; lower losses under agroecology. (For effects of global food price, see Table 2.)	Drought risk = 5%/year Drought yield losses are—High CC: av. 15%, up to 30%; Low CC: av. 10%, up to 25%; No CC: av. 7.5%, up to 20%. Losses reduced by 20% under agroecology. [Based on [51]]	Linear increase in risk–High CC: doubles after 50 years; Low CC: 1.5 times after 50 years; No CC: no change. Yield losses are fixed over time.	Drought losses are contingent on multiple processes meaning no generally applicable quantification available. Plausible approximations used, including for agroecology.

av., average; CC, climate change; ha, hectare; SD, standard deviation.

^a^ Note that model parameters are approximations derived from the literature. See text and the ODD+D (S1 Appendix) for further details.

**Table 2:** Prices for key factors, their initial values, and how they change over time^a^.

Factor (units)	Function or effect	Initial value	Change over time	Notes
**Food price**				
Local food price (cents/kg)	Food price faced by farming households.	40c/kg (Given input prices (see below), this places the average farmer close to the threshold for development.)	Calculated as the production-weighted average of farmer asking prices, adjusted for global price given price transmission.	Farm gate and consumer prices assumed to be the same.
Global food price (cents/kg)	Represents price arising from global corporate agriculture: influences trend in local price via global price transmission (Fig 1).	40c/kg	General tendency to fall (most rapidly under ‘no climate change’ and most slowly under ‘high climate change’ (due to warming)) & oscillate. Drought causes price increases, with the greatest increases under ‘high climate change’ (See text for details).	The simulations aim to assess the impact of the tendency for global prices to fall and oscillate on smallholder farming. [Based on [50]]
**Inputs**				
Labour: low skilled wage ($)	Cost of a full-time farm worker (Labour time may be purchased in fractions given target yield).	Price = 180% of the cost of a basic diet for a family of four. [e.g. [63]]	Same formula (based on average local price over last 5 years), but with an additional rise of 2% per year [Based on [64]].	Peasants do not cost labour. Over time, food costs represent a smaller proportion of people’s income.
Purchased inputs: necessary inputs ($)	‘Necessary inputs’ represent expenditure required to enable production. Assumed to be scalable given target production.	Necessary inputs for max production: price/ha = 15% of a low skilled wage. [Based on [65,66]]	Under agroecology, necessary inputs for maximum production double (i.e. in proportion to increased labour requirements (Table 1)).	Necessary inputs include clothing, tool repair, building maintenance, etc [50].
Purchased inputs: fertilizer ($/kg)	Increases productivity of a plot up to 10 times [50], with diminishing returns as quantity used increases to max.	Price of 1kg = local food price/kg X 10. Max productivity at 500kg [e.g. [67–69]]. Under ‘Entre’ and ‘entre eroding’ policy: 50% subsidy.	Same formula, but price rises 1%/year. Under ‘Entre eroding’, subsidy falls by 1%/year.	‘Fertilizer’ assumed to represent all non-necessary purchased inputs (e.g. pesticides, seeds). Thus, the fertilizer:food price ratio accounts for this.
Working (i.e. draught) animals ($)	Allows one worker to farm up to 5ha (cf. manual tools, which allow 1ha to be farmed).	Price = 30 years of net income (i.e. after feeding the family) of average orphan ag farm. [Based on [50]]	Same formula, based on average local food price over the last five years.	Working animals allow workers to farm a greater area but do not increase plot productivity.
Small tractor ($)	Allows one worker to farm up to 16 hectares (cf. manual tools, which allow 1ha to be farmed).	Price = 150 years of net income (i.e. after feeding the family) of average orphan agriculture farm. [Based on [50,60]]	Same formula, based on average local food price over the last five years.	Tractors allow workers to farm a greater area but do not increase plot productivity.
Land price ($/ha)	Farmers may expand by purchasing unused adjacent plots.	Price/ha = the cost of 30 tonnes of cereal (Equivalent to the value of 30 years of average max production of orphan agriculture)	Same formula, based on average local food price over the last five years.	Price chosen as this roughly represents the gross value produced on the land over the working life of an orphan farmer.
**Credit**				
Annual interest rates (%)	Interest rates on loans for fertilizer (short-term), animals and tractors (mid-term), and land (long-term) [32].	Short-term (1 year): 20%, mid-term (3 to 6 years): 15%, long-term (8 years): 10%. Rates halved under ‘Entre’ and ‘Entre eroding’ policy.	Fixed, except under ‘Entre eroding’ policy where rates increase linearly over time, returning to their full values after 50 years.	Peasant farmers do not use credit. Rates based on [70–73].

ha, hectare.

^a^ Note that model parameters are approximations derived from the literature. See text and the ODD+D (S1 Appendix) for further details.

We ran two sets of simulation experiments and conducted a sensitivity analysis. Previous climate-health impact modelling has assessed how climate impacts on food production may in turn impact on health [for reviews, see: 9,74]; our simulations develop an alternative perspective in two stages. First, the ‘Style preference and globalization’ runs assess how different combinations of style preferences and global price transmission influence farm development trajectories—in terms of total food production, food price, and farm incomes—in the absence of climate change or specific agricultural policies. Second, the ‘Climate change and agricultural policy’ runs look at how climate change and agricultural policy may modify these development trajectories, and how these may in turn shape the conditions that support (or undermine) the health of rural communities. Following this we ran a sensitivity analysis to assess how assumptions about maximum productivity and climate-related losses of peasant-based agroecology and entrepreneurial farming influence model outputs for total food production and food price.

Below, we provide further detail on the ABM and then describe our scenarios and simulation experiments. Full details of the model are given in the accompanying ODD+D (Overview, Design Concepts and Details plus Decision-Making) [75] (S1 Appendix). The latter is a widely adopted format for giving a complete and consistently organized description of ABMs. We also provide additional details on some key assumptions in S1 Table. The model was implemented in Netlogo 6.0.1 [76].

### Model details

This section describes the following: the rural landscape, the agents, how climate change is represented, some key model processes, and the main outcomes assessed. For a brief discussion on the utility of a stylized model, as well as further details on assumptions including those around farming styles, agroecology and yields, food price, labour access, migration, and hunger and health, see S1 Table.

#### Rural landscape

The landscape is a 21 by 21 grid (441 cells) of 1ha arable plots, which represents the ‘local area’ occupied by the hypothetical rural community. Each plot is randomly assigned a maximum productive potential of between 800kg and 1200kg of cereal equivalents/year (see below for how these quantities relate to dietary intake requirements) (Based on [50]). Additionally, each plot is randomly assigned a yield multiple that may be achieved under agroecology following a transition period of three years (Table 1; for assumptions on maximum yields under agroecology, see S1 Table).

#### Agents

The agents are farming households comprised of four people (Table 1), and farms produce a generic crop measured in cereal equivalents [50] (also see S1 Table). Households may adopt peasant or entrepreneurial styles, which are represented by three ways of farming (Fig 1). ‘Orphan’ and ‘agroecology’ are sub-types of peasant style; the third way is entrepreneurial (For why a styles-approach is taken, see S1 Table, as well the Introduction and Discussion).

The distinctions between peasant and entrepreneurial farming are based on empirically-derived theoretical categories developed by van der Ploeg [32]. We note that van der Ploeg does not claim a rigid distinction between entrepreneurial and peasant farming exists in the real world. Rather, the ‘peasant condition’ is an ongoing process that develops in response to changing contextual conditions, and which may express more or less ‘peasantness’. For the purposes of the model, however, we assume entrepreneurial and peasant farming are distinct categories.

In terms of the style-related dimensions of production and reproduction, entrepreneurial farming predominately relies upon purchased farm inputs (e.g. fertilizers) and wage labour, often using credit to obtain these, and develops via capital intensification [39]. This means the logic driving production decisions is largely shaped by off-farm processes, such as price ratios (determining the margin) and technology (determining scale); thus, the market acts as an ordering principle, and the goals of entrepreneurial farming are to maximise returns-on-investment and expand (market share and/or farm size) [32].

Peasants farming differs in that a major goal is to deepen autonomy. In terms of the style-defining dimensions of production and reproduction [39] this is achieved by largely relying on on-farm produced inputs, avoiding credit, and maximising returns-to-labour, with development being via labour and knowledge intensification. Thus, farmers attempt to shape the production process such that it guarantees the next year of production without recourse to the market. In this sense, autonomy means reduced market dependence. This does not imply peasants isolate themselves from markets; rather, markets are used as an outlet for surplus production [32].

Another key difference between peasant and entrepreneurial farming is that peasants only use family labour and do not consider labour costs when optimizing production [42]; instead, they must provide a labour diet adequate for the required labour power (Table 1). In contrast, entrepreneurial farmers employ labour, paying a wage (Table 2) and costing labour in optimization decisions, including when a labourer is a family member [42] (for more on labour, see S1 Table).

The first sub-type of peasant farming is orphan agriculture (Mazoyer and Roudart [50] use “orphan” to refer to exclusion from previous development opportunities; it is not intended to suggest these farms are excluded from their communities). Following Mazoyer and Roudart [50], this is defined as farming with manual tools (e.g. a hoe) and very limited input use (e.g. fertilizers), meaning that one worker labouring at full capacity can farm 1 hectare to produce an average of 1000kg of cereal equivalents per year. Of this, 700kg provides a family of four a basic diet (~2200kcal/person/day), and full capacity labour requires an additional (i.e. additional to a basic diet) 2900kcal/labour-day, which is equivalent to ~110kg of cereal/year (Table 1). Thus, limited production potential relative to needs renders orphan livelihoods precarious.

The second sub-type of peasant farming is agroecology. In the ABM, during an agroecology transition period of three years, orphan farmers intensify the productive potential of their land (and thus deepen their autonomy) to gain an average yield multiple of four (e.g. an initial maximum yield of 1000kg/ha would be increased to 4000kg/ha) (Table 1). This is achieved by developing and modifying on-farm ecological processes, generally via labour intensification (Table 1) and learning, the latter being achieved partly during the labour process and partly through community networks. We note that these ecological processes and networks are not explicitly represented in the ABM (for more assumptions regarding agroecology, see S1 Table) [28,51].

Additionally, as peasant farming (both agroecology and orphan agriculture) is labour and knowledge intensive, slow ongoing gains in maximum productivity per hectare may also be achieved by fine-tuning farming practices (Table 1; for assumptions on incremental productivity gains, see S1 Table) [42].

For simplicity, we do not represent family reproduction, land fragmentation or migration (for assumptions related to migration, see S1 Table). Agents present at model initiation either maintain their farm, expand, or leave farming.

As well as agents representing farming households, ‘corporate agriculture’—which is large-scale agriculture with a profit-making goal [32]–is represented by an exogenous forcing term. Over recent decades, various processes—including productivity increases and subsidies—have led corporate agriculture to be associated with a general tendency for global food prices to fall, and it has been argued that this has caused poverty and untenability of livelihoods for many smallholders (i.e. both peasant and entrepreneurial farmers) [23,50,77,78]. Additionally, global prices tend to oscillate, with troughs potentially forcing the worst-off farmers permanently out of farming [50]. Given this, rather than representing corporate agriculture explicitly as farms, the model represents it implicitly as a price trend that tends to fall but oscillate (Fig 1, Table 2, S1 Table; further details ahead).

In sum, the ABM represents peasant and entrepreneurial style farming households (agents), who farm in one of three ways (orphan, agroecology, entrepreneurial), in a local area comprised of 441 one hectare plots (landscape), who collectively form a constellation of farming households that operate in a global context in which prices associated with corporate agriculture (an exogenous forcing term) tend to fall but oscillate. The context of farming is also shaped by climate change.

#### Climate change

There are multiple pathways from climate change to nutrition [41], and different agricultures in different parts of the world are expected to face varying degrees and forms of change in weather and climate [79]. In the ABM, however, as we aim to look at patterns in the results rather than quantify outcomes, we incorporate climate using a simple approach. We consider three climate change scenarios (‘no’, ‘low’, and ‘high’), with each of the warming scenarios being associated with a linear increase in temperature (equivalent to 1°C and 2°C of warming over 50 years in the low and high scenarios, respectively) and a rise in drought risk, with the same changes experienced on all plots of land (Table 1).

Climate affects farmers in the local area as well as global food price (i.e. corporate agriculture). For farmers in the local area, climate change is expressed as yield losses. As temperature rises, yields decline on all plots. If a drought occurs, yield losses vary randomly (around an average loss) by farm. Agroecology farms face lower temperature-related and drought losses as the diverse on-farm ecology confers greater resilience [51] (Table 1). For global food price, temperature rise and droughts lead to price increases (Fig 1, Table 2).

#### Model processes

Each time step represents one year during which a set of processes associated with production occur sequentially (Fig 1). At the start of each time step, farms have a potential income given what they produced in the previous time step and the local food price, and (possibly) savings. Following this orphan farmers decide whether to convert to their preferred style of farming. Those who prefer peasant style agroecology will begin conversion if their savings are sufficient to cover the additional inputs required during the labour-intensive transition period (i.e. additional labour diet and necessary inputs). The use of savings means they will not be dependent on credit. Those who prefer entrepreneurial style will convert if their income (after feeding the family) plus their savings will cover a low skilled wage, which is assumed to make them eligible for credit (e.g. to purchase fertilizer).

Following this, agroecology and entrepreneurial farmers decide whether to expand their farm, by acquiring land, working animals or small tractors (Table 2). Agroecology farmers will gradually acquire up to two lots of working animals and 10ha of land as this is manageable using family labour (equivalent to two full time workers). They will only acquire new land or animals if all their existing plots have been transitioned to agroecology (thus, the maximum rate of expansion is 1ha every 3 years) and if all costs can be met using savings. Entrepreneurial farmers will acquire land, working animals or tractors if their income and savings cover at least half the cost, using credit to cover the balance (Table 2, main text). They may acquire 1ha of land per year. When acquiring land, all farmers choose the plot with the highest productive potential that is contiguous with their farm.

Next farmers allocate resources to consumption and production via endogenous processes (for assumptions on consumptions, see S1 Table). Each farmer estimates their expected food price in the coming year, based on current price, the price change over the previous five years, style-specific considerations, and some random variation (representing unmodelled factors that may affect expectations). Farmers then find their target level of production using standard economic methods [80,81], but with the following style-specific modifications (See S1 Appendix for additional details, including Figures B and C which show decision-type trees for resource allocation; S1 Table).

Peasant farmers initially aim to maximise returns-to-labour, which is equivalent to optimizing without costing labour [42]. However, if income at this level of production would not meet their autonomy-related goal of increasing value added per labour object (i.e. increase net income per hectare), they will attempt to produce at a higher level. If necessary, households ration resources between consumption and production, and if they are unable to provide themselves with at least 50% of a basic diet they will either sell assets (if owned) or abandon the farm.

Entrepreneurial farmers first assess whether their income plus savings is sufficient to meet their current debt obligations and provide at least 50% of a basic diet for the family. If not, they sell assets (if owned) or abandon the farm. Following this, they find optimal production by maximising returns-on-investment [42]. If, however, either (i) the farm would run at a loss at this level of production, they will sell assets (if owned) and re-optimize or abandon the farm, or (ii) farm income at this level of production would not meet their expansion-related goals, they will attempt to increase production, again selling assets if necessary (Also see S1 Table for style specific differences in the use of land, labour and other resources).

All farmers then attempt to produce their target yield, with actual yield being determined by climate effects and random variation (Fig 1). The model accounts for expected annual increases in temperature and drought risk; calculates expected yield losses due to warming; and, assesses whether a local drought occurs (given drought risk) and, if so, the expected average yield losses (Table 1). Actual yield for each farming household is then calculated given climate change-associated losses and random variation (of ± 15% to account for unmodelled factors).

Given their actual yield, each farming household now calculates their asking price. In doing so, both peasant and entrepreneurial farmers seek to maintain their respective autonomy- and expansion-related goals (i.e. via endogenous processes). An initial aggregate local price is then calculated by combining the asking prices of each household to give a production-weighted average. Finally, this initial local price is adjusted for global price (see below) according to scenario-specific global price transmission (an elasticity) (Fig 1); for example, if global food price had risen by 5% and global price transmission were 0.5, then local food price would be increased by 2.5% (Also see S1 Table).

Global food price is exogenously set such that it has a tendency to fall and oscillate, but will rise in response to a drought. The average rate of price decline is determined by the climate scenario: 1.5%/year, 1.25%/year and 1%/year under ‘no’, ‘low’ and ‘high’ climate change, respectively (The actual change in each time step is randomly determined and includes the possibility of a price rise). This tendency is combined with an oscillator function has an amplitude of 1.5 cents and period of 10 years (These parameters were chosen subjectively by observing price behaviour while varying their values). Finally, the model assesses whether there is a drought that affects global prices. If a drought occurs, price is adjusted upwards by a random amount dependent on the climate change scenario (5% to 7.5%, 7.5% to 12.5%, and 10% to 17.5%, under no, low, and high climate change, respectively).

Local food price and farm production are then combined to estimate the incomes of each farming household, the next time step begins, and the model processes are repeated. Each simulation is run for 50 years (i.e. time steps).

#### Outcomes assessed

The following outcomes are tracked by the model and presented in the results. ‘Local food price’ is the price faced by farming households (farm-gate and consumer prices are assumed to be equal), calculated as described above. ‘Total food production’ is the total physical product of the entire farming community, expressed in tonnes of cereal equivalents. ‘Income slope’ is the average change in income over the previous ten years (i.e. slope as $ per year) for farmers practicing each style; that is, it indicates whether incomes are rising, stable, or falling, and the magnitude of the change. ‘Converted farms’ is the number of orphan farmers who have converted to their preferred style. ‘Abandoned farms’ is the number of households who left farming as they cannot provide themselves with 50% of a basic diet or meet their debt obligations.

Five health-related outcomes (i.e. that support the health of the farming community) are also tracked (Also see S1 Table for assumptions on both hunger and health). ‘Orphan nutrition’ is the average proportion of a basic diet (in calories; Table 1) available for remaining orphan households. ‘Labour’ is the sum of full-time equivalent workers (including both workers on peasant farms and wage earners) on farms in the community. ‘Income Gini’ is a measure of income inequality amongst farming households in the community, and ‘mean net farm income’ is the average net income across all farming households in the community.

The fifth outcome is ‘real land productivity’ which is a measure of farming intensity based on value added during the farming process; that is, it removes the contribution of inputs that were produced elsewhere (e.g. purchased fertilizers) [32,65]. The latter were produced in environmental spaces other than the farm, and during the farming process their value is—in effect—transferred into final yield (rather than created on the farm). Thus, real land productivity is more environmentally-sensitive than conventional measures of intensity.

It is represented as net income per hectare adjusted for the proportion of value that was added on the farm (‘endogeneity’), calculated [based on 65] as:

$$real\;land\;productivity\;[\$/ha]=\frac{farm\;net\;income\;[\$]}{farm\;size\;[ha]}\times endogeneity$$ (1)

$$\begin{array}{l} endogeneity=\frac{value\;added\;on\;the\;farm\;[\$]}{value\;of\;total\;farm\;production\;[\$]}\\ \;\;\;=\;\frac{value\;of\;total\;farm\;production-(purchased\;inputs\;excluding\;labour)\;[\$]}{value\;of\;total\;farm\;production\;[\$]} \end{array}$$ (2)

### Scenarios, experiments, and sensitivity analysis

#### Model set-up: Scenario settings and initialization

Prior to initialization, a scenario is chosen by the model user, which is a combination of four factors (Fig 1). First, ‘Farming style preference distribution’ is the proportion of orphan farming households who prefer peasant style and aim to develop via agroecology (rather than entrepreneurial style). Second, ‘Global price transmission’ is the degree to which global food prices influence local food prices. This is an elasticity that specifies the percent change in local price given a 1% change in global price [82]. Third, ‘Climate change’ may be set to ‘no’, ‘low’ or ‘high’ (Table 1).

Fourth, ‘Agricultural policy’ specifies which farming style is favoured and has four options (see Table 3, ahead). ‘Entrepreneurial’ policy favours entrepreneurial farming by lowering interest rates and fertilizer prices (Table 2). ‘Entrepreneurial eroding’ is initialized in the same way but interest rates and fertilizer prices rise linearly to their unsubsidized levels after 50 years (Table 2). ‘Peasant’ policy favours orphan agriculture and agroecology by supporting research and fostering community networks, which is represented in the model by a rise in the rate of annual maximum yield increase (Table 1). ‘None’ means policy does not favour any style.

**Table 3:** Agricultural policy scenarios and associated settings for proportion preferring agroecology and global price transmission.

Agricultural policy		Prop preferring agroecology	Global price transmission
Policy name	Policy actions		
**Entrepreneurial**	Lower interest rates and fertilizer subsidies (see Table 2).	0.25	0.75
**Entrepreneurial eroding**	As for entrepreneurial except interest rates and subsidies linearly increase and return to baseline level after 50 years (see Table 2)	0.25	0.75
**Peasant**	Support for research as well as development of community networks, represented by increased rate of yield increases (see Table 1).	0.75	0.25
**None**	No actions supporting any farming style.	0.5	0.5

The model is initialized by placing each of 250 peasant households practicing orphan agriculture on randomly selected 1ha plots in the local area of 441 plots (Table 1). At initialization, it is assumed that all households have achieved their maximum yield and have no savings. Each household is randomly assigned a (fixed) preference for whether they will aim to develop by remaining peasants and adopting agroecology style, or, by adopting (non-peasant) entrepreneurial style, with the preference distribution being user-selected (Fig 1). Additionally, households are randomly assigned preferences for how they save money and whether they favour production or family nutrition when rationing.

Local and global food prices are set, then prices for productive commodities (e.g. labour, fertilizer, land) are set based on food price (Table 2). That is, productive commodity prices are linked to food price, but many of these links change over time (Table 2) as, for example, it is assumed that food prices represent a decreasing share of wages. Of note, initial prices are intentionally set at levels such that the average orphan farmer is close to an income that would allow them to develop their farm. The temperature anomaly (i.e. warming) is set to 0 and drought risk is set at 5% per year for both the local area and corporate agriculture (i.e. global food price) (Table 1).

#### Simulation experiments

We conducted two sets of simulation experiments. The ‘Style preference and globalization’ simulations were run without climate change (i.e. ‘no’ climate change) or specific agriculture policies (i.e. ‘none’ policy) for various combinations of proportion preferring agroecology and global price transmission. The purpose was to assess how the latter two factors influence farm development trajectories in terms of local food price, total food production, and the income trajectories of farmers practicing each style.

The second set—the ‘Climate change and agricultural policy’ runs—then look at how climate change and four agricultural policy scenarios (Table 3) modify farm development trajectories, and how this may in turn shape both patterns of hunger and conditions that support the health of the farming community.

The four agricultural policy scenarios are intended to approximate the following: (i) ‘entrepreneurial’ represents worlds favouring capital intensive farming that is highly dependent on and integrated into globalized markets (e.g. some farms practicing ‘sustainable intensification’ have these characteristics [30]); (ii) ‘entrepreneurial eroding’ recognises that the development trajectory of entrepreneurial farming is at least partly dependent on conditions external to farms and assesses the consequences if these conditions are not maintained over the long term [cf. 32]; (iii) ‘peasant’ represents worlds in which on-farm ecological processes are enhanced via labour intensification in order to develop both production and farmer autonomy, with agroecology being a key farming practice for achieving this [51]; and, (iv) ‘none’ represents a world where entrepreneurial and peasant farming co-existence but there is no explicit policy support for either.

For both sets of simulation experiments the ABM was run 250 times (which was judged—based on observation of outputs and across-run standard deviations—to be sufficient to capture typical model behaviour) for each combination of factors and the results for each output are shown as their across-run mean values.

#### Sensitivity analysis

In the ‘Climate change and agricultural policy’ simulations, the differences in the outcomes for peasant- and entrepreneurial-centred futures are of key interest. The model has many parameters, and naturally we cannot evaluate the sensitivity of the model outputs to all of them. However, two aspects of the model parameterization may have a strong influence on these results.

Firstly, agroecology to entrepreneurial yield ratios. In the simulation experiments, it is assumed that (i) transitioned agroecology farms may initially produce up to an average of 4 tonnes per ha (SD = 1.5) and that this may slowly increase up to a maximum 10 tonnes per ha, and, (ii) entrepreneurial farming may produce up to an average of 10 tonnes per ha (Table 1). That is, the average agroecology to entrepreneurial yield ratio is initially 2:5 and may increase over time to 1:1.

Secondly, it is assumed that climate change-related losses for agroecology are lower than entrepreneurial losses: 10% lower for warming-related losses and 20% lower for drought-related losses (Table 1).

We conducted a sensitivity analysis to assess the influence of these assumptions on two key outcomes: total food production and local food price. Under the ‘entrepreneurial’ and ‘peasant’ policy scenarios (Table 3), we re-ran the model under the following conditions: (i) fixed agroecology to entrepreneurial yield ratios of 1:4, 1:2, 3:4, and 1:1, with no increases in agroecology yield over time, and (ii) warming- and drought-related yield losses for agroecology compared to entrepreneurial of 10% lower, equal, and 10% higher.

## Results

### Style preference and globalization runs

**Fig 2:**
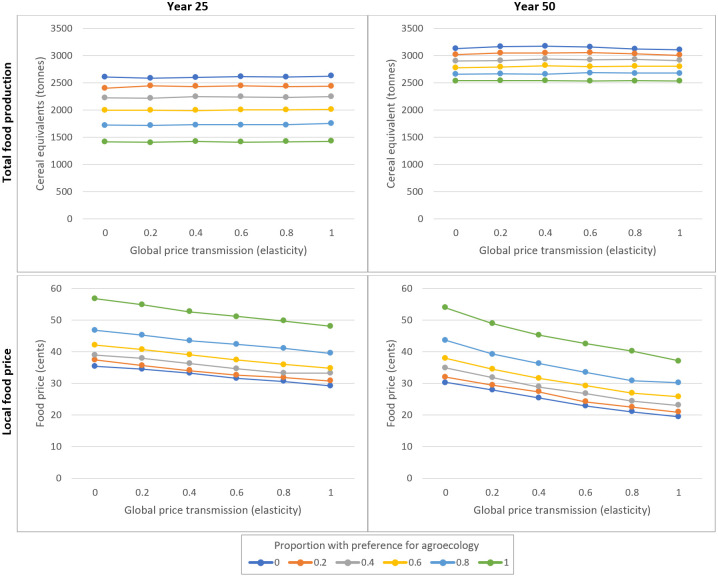
Total food production and local food price in the absence of climate change and agricultural policies. The plots show total food production (top two plots) and local food price (bottom two plots) (y-axes) under combinations of global price transmission (x-axes) and proportion preferring agroecology (line colour) after 25 (left plots) and 50 years (right plots), in the absence of climate change or specific agricultural policies. For the y-axes, total food production and local food price are shown as the mean result across 250 runs under each combination of factors. For the x-axes, global price transmission is an elasticity such that a value of 0.6, for example, means that a 1% rise in global food price would cause a 0.6% rise in local food price. For the line colours, a value of 0.2, for example, means that 20% of orphan farmers prefer to develop via agroecology and 80% via entrepreneurial farming.

These simulations assess how patterns of farming styles influence food production. More specifically, they assess how farm development trajectories—in terms of production, price, and incomes—are influenced by patterns of farming style preference and global price transmission, in the absence of climate change and particular agricultural policies. Fig 2 shows total food production and local food price (y-axes) under various combinations of global price transmission (x-axes) and proportion preferring agroecology (line colour) at 25 and 50 years.

The proportion preferring agroecology had a strong effect on total production (i.e. production summed across all farms) at 25 years (Fig 2, upper left panel), with production in runs where 80% preferred agroecology being 25% lower than when 80% preferred an entrepreneurial style. This gap declined by year 50, with production in the former being 10% lower than in latter (Fig 2, upper right panel). Global price transmission tended to have little effect on total food production.

Local prices tended to be lower at 50 years compared to 25 years (Fig 2, bottom row). Prices were lower when global price transmission increased, with the latter effect being stronger at 50 years compared to 25 years. An increase in the proportion preferring agroecology increased prices at both 25 and 50 years. Compared to the start price (i.e. at year 0) of 40c per kg, prices tended to be lower at both 25 and 50 years under most sets of conditions, except when a very high proportion preferred agroecology and/or when price transmission was very low.

**Fig 3:**
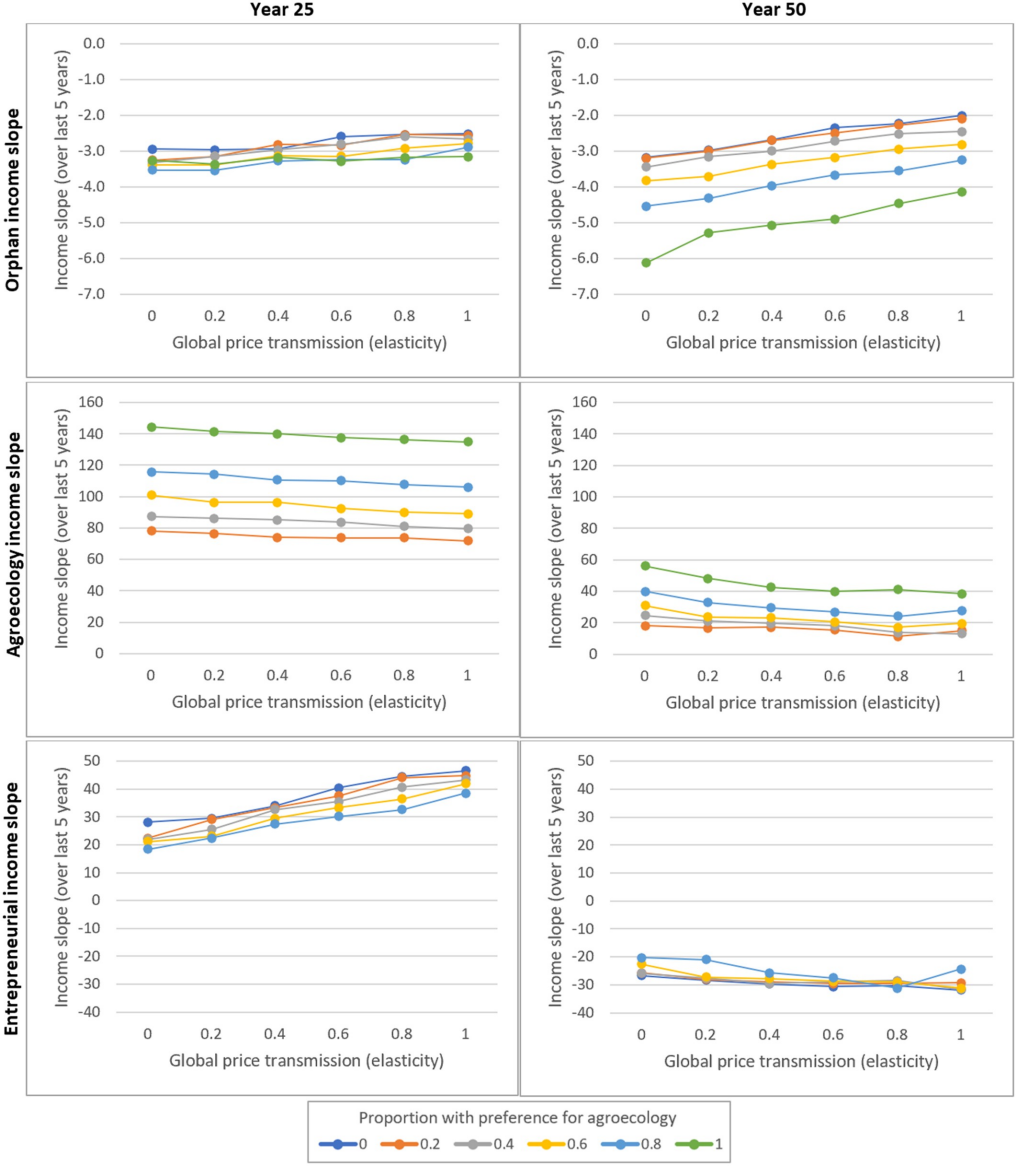
Income slopes by farming style in the absence of climate change and agricultural policies. The plots show income slopes for orphan, agroecology, and entrepreneurial farms (top to bottom plots, respectively) (y-axes; scale differs for each style) under combinations of global price transmission (x-axes) and proportion preferring agroecology (line colour) after 25 (left plots) and 50 years (right plots), in the absence of climate change or specific agricultural policies. For the y-axes, the income slopes are the gradient (units = $ per year) of mean farm net income by farming style over the previous ten years, shown as the mean result across 250 runs under each combination of factors. For the x-axes, global price transmission is an elasticity such that a value of 0.6, for example, means that a 1% rise in global food price would cause a 0.6% rise in local food price. For the line colours, a value of 0.2, for example, means that 20% of orphan farmers prefer to develop via agroecology and 80% via entrepreneurial farming.

Fig 3 shows the rate of change of farm net incomes (averaged over the previous 10 years) in $ per year, by farming style (i.e. as average change across all farmers practicing a given style) (y-axes), under various combinations of global price transmission (x-axes) and proportion preferring agroecology (line colour), at 25 and 50 years. For reference, the average orphan household would have a net income of about $80 per year after providing a basic family diet plus a labour diet if local food price were 40c per kg (i.e. the food price at year 0).

Net incomes for orphan agriculture tended to be falling slowly at both 25 and 50 years (Fig 3, top row). At 25 years, the fall in income tended to increase slightly as the proportion preferring agroecology increased, and to decrease slightly as global price transmission increased. This pattern strengthened at 50 years. For farmers practicing agroecology, net incomes tended to be increasing rapidly at 25 years, with this increase slowing at 50 years (Fig 3, middle row). Increasing price transmission tended to slow growth slightly, and increasing the proportion preferring agroecology tend to increase growth, with the latter effect being stronger at 25 years than at 50 years.

For entrepreneurial agriculture, incomes tended to be rising at 25 years, although at a slower rate than for agroecology farmers (Fig 3, lower left panel). Increasing transmission increased growth, and increasing the proportion preferring agroecology decreased it, albeit both effects were reasonably small. At 50 years, these tendencies had reversed: incomes were falling; transmission tended to steepen the fall; and, an increasing in the proportion preferring agroecology slowed the fall (Fig 3, lower right panel). These latter two effects, however, were very small.

### Climate change and agricultural policy runs

The second set of simulations has two parts. Firstly, we assessed how farm development trajectories are modified by climate change and agricultural policy scenarios, where the latter are a combination of an agricultural policy plus related settings for the proportion preferring agroecology and global price transmission (Table 3). Secondly, we assessed how these farm development trajectories impact on hunger and a set of conditions that support health in the rural community.

**Fig 4:**
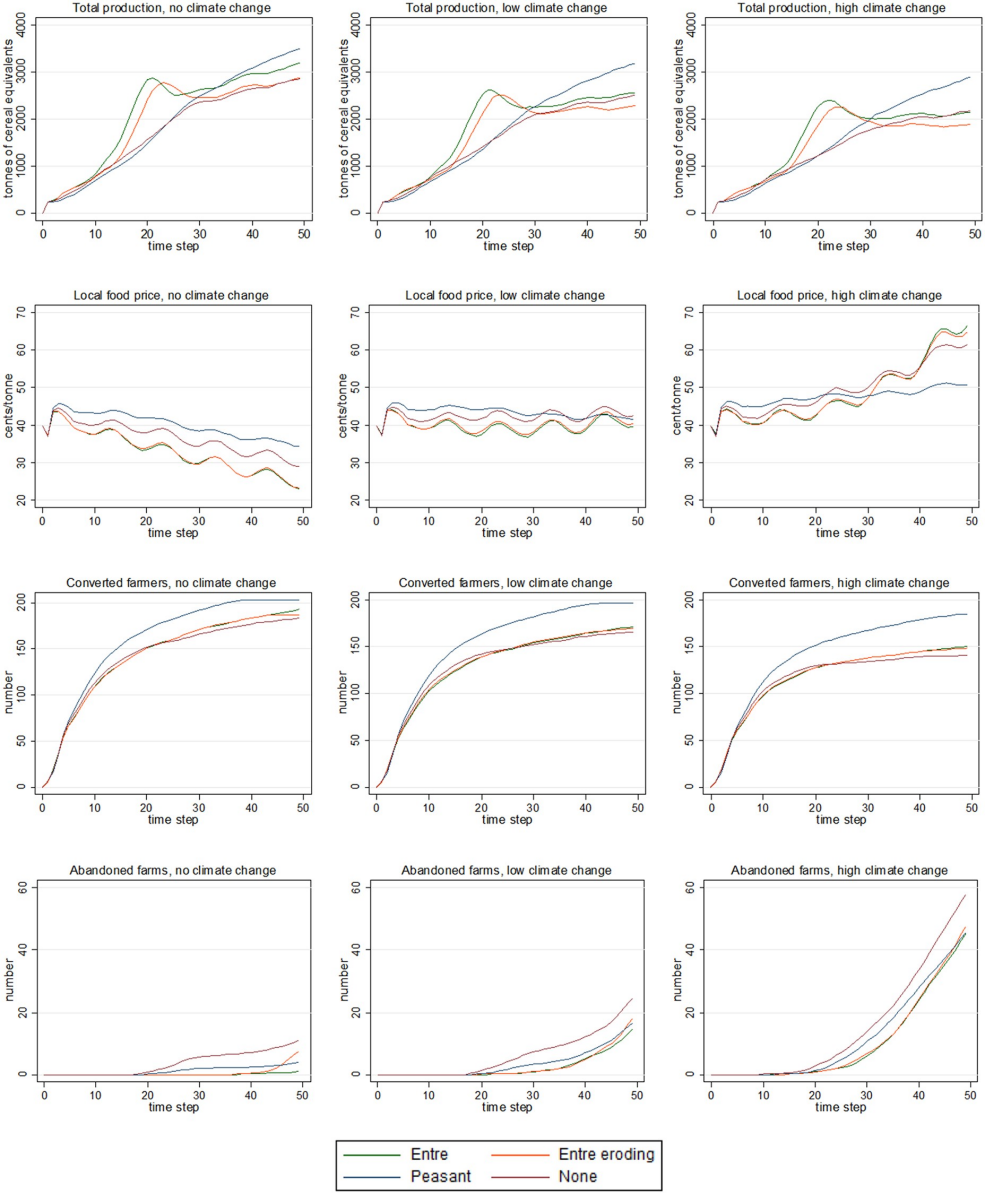
Total food production, local food price, and converted and abandoned farms, under the agricultural policy and climate scenarios. The plots show time-series for total food production, local food price, and the number of converted and abandoned farms (top to bottom plots, respectively) under the four agricultural policy scenarios, for no, low, and high climate change (left to right plots, respectively). For the y-axes, all results are shown as the mean value across 250 runs. For the coloured lines, the four scenarios are: (i) ‘Entre’ in which agricultural policy favour entrepreneurial farming, 25% of farmers prefer agroecology, and global price transmission is 0.75; (ii) ‘Entre eroding’ is as for ‘Entre’ except policy support erodes over time; (iii) ‘Peasant’ in which policy favours peasant farming, 75% of farmers prefer agroecology, and global price transmission is 0.25; and, (iv) ‘None’ in which policy favours neither farming style, 50% prefer agroecology, and global price transmission is 0.5 (Table 3).

Fig 4 shows time-series plots (covering 50 years; x-axes) for farm development trajectories, as total food production (i.e. summed across all farms), local food price, the number of farmers who have converted to their preferred style, and the number of abandoned farms. Results are shown as the mean result over 250 runs (y-axes) for each policy scenario (coloured lines).

In futures without climate change, total production (Fig 4, top row) rises rapidly for the first 20 years under both entrepreneurial scenarios. It then falls for a short period before again beginning to slowly rise (with production gains slower under ‘entrepreneurial eroding’ than ‘entrepreneurial’). In contrast, production rises slowly but steadily under the ‘peasant’ scenario, with production beginning to exceed that under ‘entrepreneurial’ after about 35 years. This pattern is similar under low and high climate change, but the final gap between ‘peasant’ and other scenarios increases as climate change worsens.

Local food price (Fig 4, second row) is falling under all policy scenarios in worlds without climate change, and is highest under the ‘peasant’ scenario. Under low climate change, prices tend to be fairly stable over time, and are similar under all policy scenarios after 50 years. Under high climate change, prices initially rise slowly and then begin to rise rapidly under all but the ‘peasant’ scenario after about 30 years.

The number of converted farmers (i.e. households who have been able to move from orphan farming to their preferred style) (Fig 4, third row) grows fastest under the ‘peasant’ scenario, with the gap between the latter and other scenarios growing across the no, low, and high climate scenarios, respectively. The number of abandoned farms (i.e. households who were unable to provide themselves with at least 50% of a basic diet or meet their debt obligations) (Fig 4, bottom row) increases with climate change (i.e. across the no, low, and high scenarios, respectively). Numbers are highest under the ‘none’ policy scenario, followed by the ‘peasant’ scenario (although by 50 years the numbers under ‘entrepreneurial eroding’ have exceeded those under the ‘peasant’ scenario).

**Fig 5:**
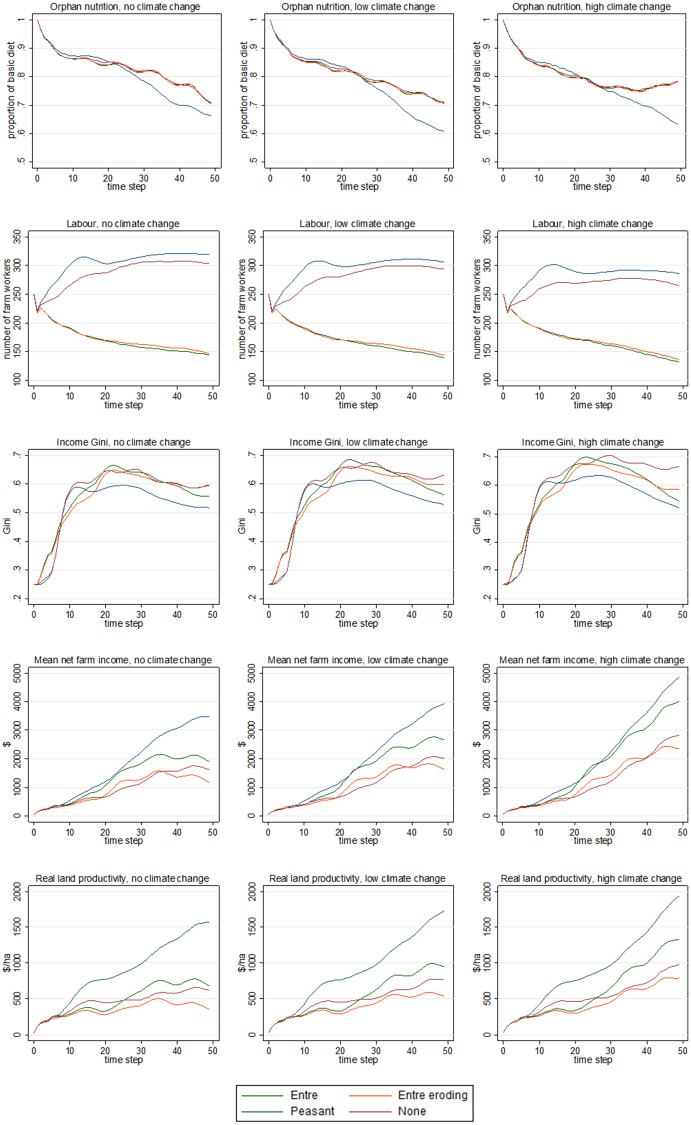
Nutrition, labour, income inequality, net farm income, and real land productivity under the agricultural policy and climate scenarios. The plots show time-series for nutrition in orphan farming households, labour, income inequality, net farm income, and real land productivity (top to bottom plots, respectively) under the four policy scenarios, for no, low and high climate change (left to right plots, respectively). For the y-axes, all results are shown as the mean value across 250 runs. For the coloured lines, the four scenarios are: (i) ‘Entre’ in which agricultural policy favour entrepreneurial farming, 25% of farmers prefer agroecology, and global price transmission is 0.75; (ii) ‘Entre eroding’ is as for ‘Entre’ except policy support erodes over time; (iii) ‘Peasant’ in which policy favours peasant farming, 75% of farmers prefer agroecology, and global price transmission is 0.25; and, (iv) ‘None’ in which policy favours neither farming style, 50% prefer agroecology, and global price transmission is 0.5 (Table 3). The outcomes are as follows—‘Orphan nutrition’: the average proportion of a basic diet being consumed in orphan farming households; ‘Labour’: the total number of full-time equivalent workers across all farming households; ‘Income Gini’: income inequality, where a higher value means greater inequality; ‘Mean net farm income’: average net farm income across all households over the previous five years;. ‘Real land productivity’: an indicator of farming intensity after removing the contribution of purchased inputs to Gross Value Product.

Fig 5 shows time-series plots (covering 50 years; x-axes) for five health-related outcomes that arise from the farm development trajectories: nutrition in orphan households, labour, income inequality, net farm income, and ‘real land productivity’, as the mean result over 250 runs (y-axes) for each policy scenario (coloured lines).

Orphan nutrition, which is the mean proportion of a basic diet consumed in the remaining orphan households (Fig 5, top row), is consistently lowest in the ‘peasant’ policy scenario, with the gap between the latter and the other policy scenarios widening across the no, low, and high climate change scenarios, respectively. Households that remain in orphan agriculture have had their development blocked (i.e. they are unable to convert to their preferred style); how this and other farm development processes (based on the results in Fig 4) impact on nutrition after 25 and 50 years is shown in Table 4.

**Table 4:** Farm development processes and their implications for nutrition, under the agricultural policy and climate scenarios at 25 and 50 years.

		Farm development process and indicator of nutrition							
		Blocked development:		Abandonment:		Realised development:		Raised total production:	
		Mean proportion of a basic diet^a^ consumed in orphan households (% of initial households in brackets)^b^		% of initial households abandoning as unable to provide ≥50% of a basic diet^a^^,^^c^		% of initial households with at least a basic diet^a^^,^^d^		Total number of households potentially fed a basic diet^a^^,^^e^	
Policy scenario	Climate scenario	25 years	50 years	25 years	50 years	25 years	50 years	25 years	50 years
**Entre**	**No**	0.83 (36%)	0.70 (22%)	0%	0%	65%	77%	3,606	4,559
	**Low**	0.80 (41%)	0.71 (26%)	0%	6%	59%	68%	3,350	3,659
	**High**	0.78 (46%)	0.78 (22%)	1%	18%	53%	60%	3,173	3,059
**Entre eroding**	**No**	0.84 (36%)	0.71 (22%)	0%	3%	64%	75%	3,829	4,111
	**Low**	0.80 (41%)	0.71 (25%)	0%	7%	59%	68%	3,530	3,260
	**High**	0.78 (46%)	0.78 (22%)	1%	19%	54%	60%	3,213	2,694
**Peasant**	**No**	0.82 (26%)	0.66 (17%)	0%	2%	73%	81%	2,914	5000
	**Low**	0.80 (30%)	0.61 (14%)	1%	6%	70%	78%	2,664	4,553
	**High**	0.78 (34%)	0.63 (8%)	2%	18%	64%	74%	2,333	4,124
**None**	**No**	0.84 (35%)	0.71 (22%)	1%	4%	64%	74%	2,891	4,087
	**Low**	0.81 (39%)	0.72 (24%)	2%	10%	59%	66%	2,559	3,586
	**High**	0.78 (44%)	0.78 (21%)	3%	23%	53%	56%	2,181	3,106

^a^ A basic diet for the farming household (assumed to be comprised of four people) requires 700kg of cereal equivalents; 200kg of cereal equivalents provides 2200 kcal/day for a year [50].

^b^ The numbers show the mean proportion of a basic diet consumed across all remaining orphan households (For the corresponding time-series, see top row in Fig 5); the numbers in brackets are the percent of initial households that remain in orphan agriculture.

^c^ These results are based on the number of abandoned farms (see bottom row in Fig 4 for the corresponding time-series) as part of the criteria for abandonment is the inability to provide the family with at least 50% of a basic diet (Table 1).

^d^ These results are based on the number of farmers who have converted to their preferred style (agroecology or entrepreneurial) (see third row in Fig 4 for the corresponding time-series) as the results indicate that all these households are able to provide a basic family diet (results not shown).

^e^ These results are based on total production (i.e. across all farms; see top row in Fig 4 for the corresponding time-series), calculated as total production divided by 700kg.

The ‘blocked development’ columns are based on the orphan nutrition results reported in Fig 5 (top row) but also show the percent of initial farmers who are still practicing orphan agriculture. The results show the average fraction of a basic diet being consumed by orphan (i.e. subsistence) farmers is lowest under ‘peasant’ policy; however, the percent of farmers remaining in orphan agriculture is also lowest under this policy. The ‘abandonment’ column shows the percent of farmers who have abandoned their land as they have access to <50% of a basic diet: that is, this group have left farming as they were faced with starvation. Abandonment due to starvation rises when moving from no to low to high climate change, and is highest under the ‘none’ policy.

The ‘realised development’ columns show the percent of initial farmers who have been able to convert to their preferred style (agroecology or entrepreneurial). The model results indicate that these households are consistently able to meet basic dietary requirements (results not shown); thus, these numbers show the percent of initial households with at least basic nutrition. The numbers are highest under the ‘peasant’ policy, and decline when moving from no to low to high climate change. Finally, the ‘raised total production’ columns show how many households could be fed a basic diet given the total production across all farms. Numbers are highest under the entrepreneurial scenarios at 25 years, but at 50 years are highest under the ‘peasant’ scenario and—for low and high climate change—lowest under ‘entrepreneurial eroding’.

These same farm development processes also generate a wider set of conditions that support (or undermine) the health of the rural community. Labour, as the number of full-time equivalent farm workers across all farms (Fig 5, second row), rises rapidly under the ‘peasant’ scenario before plateauing, with a similar trajectory under the ‘none’ policy scenario. In contrast, labour continually falls under the two entrepreneurial scenarios. Climate change reduces labour in all policy scenarios. Income inequality, as the Gini coefficient (Fig 5, third row), initially rises rapidly then slowly declines under all policy and climate scenarios. Inequality tends to be the highest in the ‘none’ policy, under which it increases when moving from no to low to high climate change. In the ‘peasant’ scenario, both peak inequality and inequality at 50 years are the lowest (compared to other policy scenarios).

Average net farm incomes (Fig 5, fourth row) rise steadily under all policy scenarios under no climate change. After about 25 years, incomes under the ‘peasant’ scenario begin rising faster than those under the other policy scenarios, and are the highest at 50 years (at this time they are lowest under ‘entrepreneurial eroding’). Similar patterns are seen under low and high climate change, but incomes are higher (as prices as higher; Fig 4, second row). Patterns for ‘real land productivity’ (Fig 5, bottom row) are similar to those for net farm incomes, but gaps between the ‘peasant’ scenario and the other policy scenarios are wider.

### Sensitivity analysis

**Fig 6:**
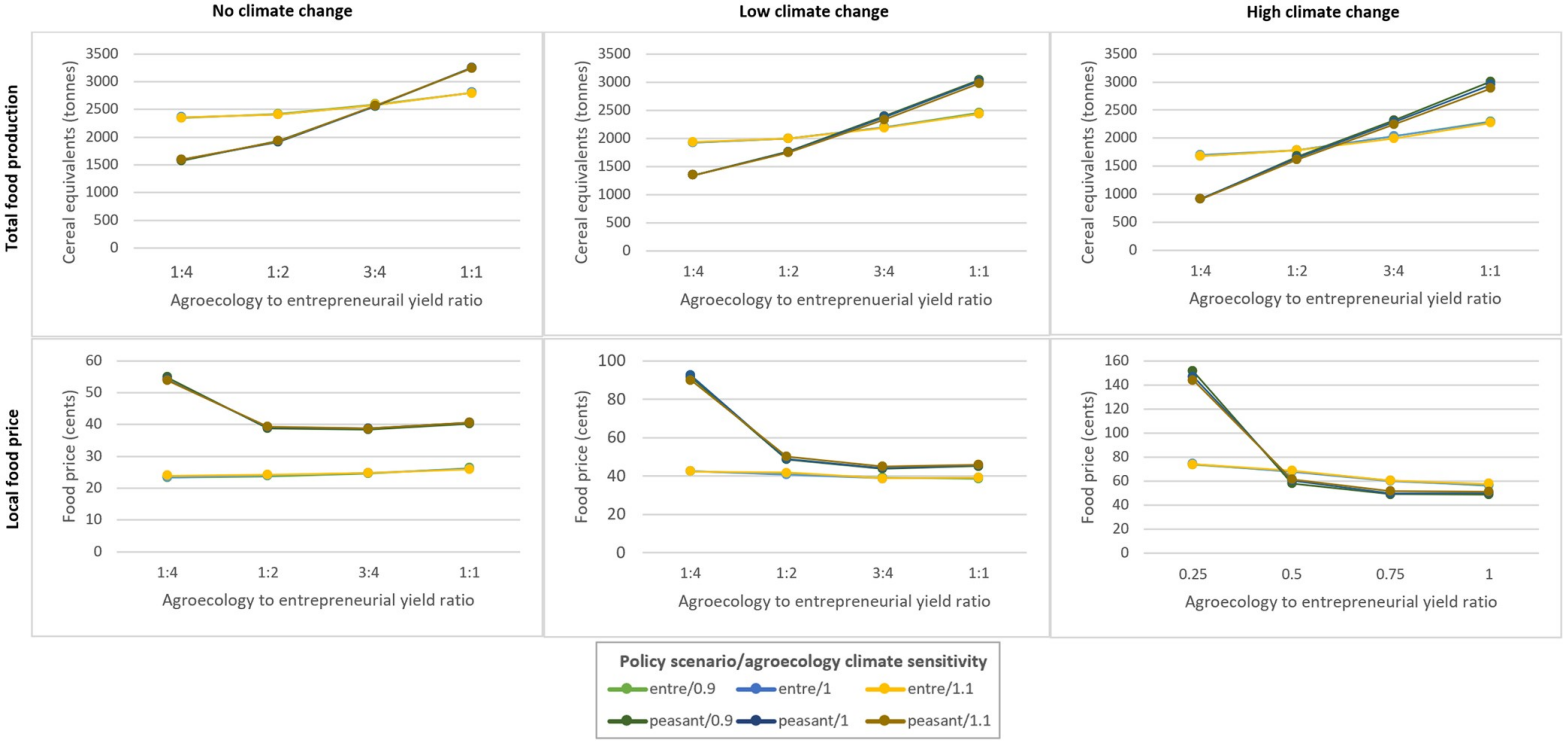
Total food production and local food price at year 50: Sensitivity analysis. The plots show total food production (top row) and local food price (bottom row) (y-axes) after 50 years, under no, low, and high climate change (left to right plots, respectively), under different yield ratio assumptions (x-axes), for the peasant and entrepreneurial policy scenarios with different climate sensitivity assumptions (line colour). For the y-axes, values are shown as the mean result across 250 runs under each combination of factors (Note that the y-axis scale for local food price differs in each plot). For the x-axes, the numbers show the ratio of agroecology to entrepreneurial maximum production (e.g. 1:4 means maximum production for agroecology is 25% that of entrepreneurial farming). For the coloured lines, ‘entre’ and ‘peasant’ refer to the entrepreneurial and peasant scenarios, respectively, and, the numbers refer to agroecology climate sensitivity relative to entrepreneurial farming, where: 0.9 means agroecology losses are 10% lower, 1 means losses are equal, and 1.1 means agroecology losses are 10% higher.

We tested how changing the assumptions about the maximum production and climate sensitivity of agroecology and entrepreneurial farming influenced total food production and local food price under the ‘entrepreneurial’ and ‘peasant’ policy scenarios (Fig 6).

After 50 years in futures without climate change, food production is 50% higher in the ‘entrepreneurial’ compared to the ‘peasant’ scenario when the agroecology to entrepreneurial yield ratio is 1:4 (Fig 6, upper left panel). The gap closes to 25% when the yield is ratio of 1:2, and production is equal when the ratio is 3:4. At a ratio of 1:1, total production in the peasant scenario is 15% higher than in the entrepreneurial scenario.

In futures with climate change, food production under the peasant scenario rises relative to that under the entrepreneurial scenario, with the climate sensitivity assumptions having only a small effect (Fig 6, coloured lines). When the yield ratio is 1:2, production under the peasant scenario is 14% and 8% lower than in the entrepreneurial scenario under low and high climate change, respectively (Fig 6, upper middle and right panels, respectively). When the yield ratio is 3:4, production in the peasant scenario is 7% and 11% higher than in the entrepreneurial scenario under low and high climate change, respectively. The latter figures rise to 20% and 22%, respectively, when the yield ratio is 1:1.

For local food price, in futures without climate change, prices are 35% higher under the peasant scenario relative to the entrepreneurial scenario for all yield ratios except 1:4, where prices are 55% higher (Fig 6, lower left panel). In futures with climate change, prices under the peasant scenario remain considerably higher than in the entrepreneurial scenario when the yield ratio is 1:4. For the remaining yield ratios, peasant scenario prices are 15% to 20% higher than entrepreneurial prices under low climate change. Under high climate change, peasant scenario prices are 10% to 20% lower than those in the entrepreneurial scenario.

## Discussion

In this paper we have presented the first (at least to our knowledge) ABM that assess how patterns of farming styles may influence the relation between climate change, hunger, and rural health. This standpoint has offered a number of insights that are complementary to those gained in previous climate-health impact and farm household modelling (For the utility of a stylized model, see S1 Table).

**Fig 7:**
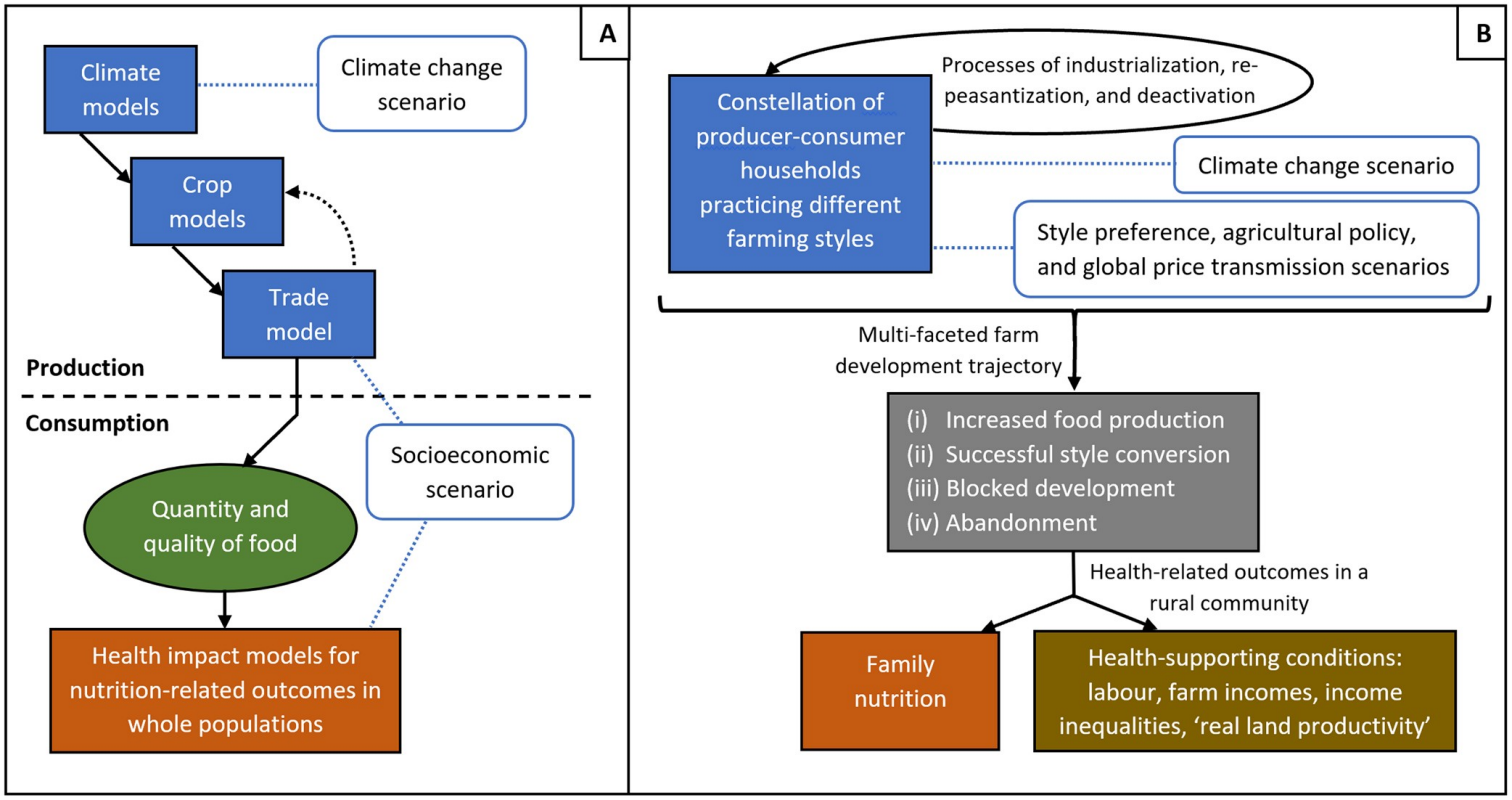
Alternative climate-undernutrition model structures based on different standpoints. Panel A shows the general structure underlying previous global-level climate-undernutrition models, which link together a series of component models. A pathway is traced from climate, to crops, to trade, to nutrition-related health outcomes. Production and consumption are separated, with the upstream component models calculating food availability (i.e. production). The health-impact model then combines the latter with socioeconomic variables to estimate consumption-related outcomes in entire populations. Panel B shows the model structure adopted in this paper. Constellations of producer-consumer farmers practicing different styles of farming develop over time under given climate, policy, style preference, and price transmission scenarios. Different facets of the farm development process give rise to patterns of hunger and other health-supporting conditions in the rural community.

Previous climate-health impact modelling essentially traces a pathway from climate change to nutrition amongst consumers, via changes in quantity and quality of food produced, where socioeconomic factors are seen as modifiers of these linkages [e.g. 12,14,17] (Fig 7, Panel A). Our model adopts an alternative standpoint (which is more in line with farm household modelling), beginning with processes that shape both wealth and poverty—as well as both good nutrition and hunger—amongst subsistence farmers, and then assessing how climate change may influence these. Farm development trajectories are at the centre of the model, and these arise from the confluence of three underlying processes: ‘industrialisation’, in which farming increasingly depends on purchased inputs (i.e. entrepreneurial farming increases); ‘re-peasantization’, in which peasant farming is strengthened via, for example, greater autonomy (i.e. agroecology farming increases); and ‘deactivation’, in which land is taken out of production (i.e. farms are abandoned) [32]. The resulting farm development trajectories manifest as changing constellations of households practicing different styles of farming, and these in turn give rise not only to patterns of nutrition but to a set of conditions that support the health of the rural community as well as vulnerability to climate change (Fig 7, Panel B). In sum, we aimed to gain new insights by shifting the standpoint of the model from that of the pathway between climate change and hunger to one based on farming styles and rural health.

Previous farm household modelling has utilised sophisticated methods to represent real producer-consumer communities, including the consideration of empirically-derived farm typologies based on statistical methods such as principle component and archetypal analysis [e.g. 34,36,37,38]. Our style-based approach differs in that it considers theoretically-derived generalizations (based on empirical research) that are potentially applicable across countries regardless of their income level [32,48] (cf. van Wijk et al’s suggestion that farm household modelling ‘should avoid the trap of developing complicated models for site-specific analyses that [are] difficult to apply to other sites because of data demands’ [33]). Arguably, a styles-based approach may be better suited to (or at least offer a useful perspective when) assessing questions around the transformation to a ‘sustainable the food system’ (what the latter would entail has different meaning to different groups [3,30]); e.g. what might be the implications of a global trend towards peasant agriculture? At the same time, however, we intentionally take a relatively simple approach in many aspects of our model; future work should draw on the well developed methods (including for decision making and risk analysis) currently used in farm household modelling.

While we have used ABM as the modelling method to illustrate the relevance of farming styles to future hunger and health, we do not suggest it is necessary to adopt ABM in future work. Rather, we suggest that key ideas raised in this work should be considered when developing future global-level models, whatever the modelling method adopted. Key among these is that while a person’s dietary intake influences their individual risk of disease, farm development trajectories—as manifest in the patterns and viability of different farming styles—shape the distribution of the risk of poor health in populations (cf. [83]). The latter approach casts people in groups that share particular risks—i.e. as sub-groups of producer-consumers—rather than as (essentially) homogenous consumers. Any modelling method able to capture these aspects could be adopted.

### Main findings

The ‘Style preference and globalization’ model runs assessed how patterns of farming styles influence production, price and farm incomes and were conducted in the absence of climate change and specific agricultural policies. That is, in contrast to previous climate-health impact modelling which assesses how production and price impact on hunger, we initially take a step back to assess how patterns of farming styles impact on production and price.

Four key patterns were seen, relating to: (i) the influence of farming style preference patterns of food production and price; (ii) the effects of global price transmission on food prices; (iii) differences in farm incomes by style and over time; and (iv) the mixed fates of the worst-off households depending on the style preference pattern. Table 5 describes the relevant patterns, their implications, and provides comments on the underlying mechanisms.

**Table 5:** The four key patterns seen in the results of the ‘Style preference and globalization’ model runs, their implications, and comments on the underlying mechanisms.

Pattern in the results	Implications	Comments on mechanisms^a^
***(i) Food production and food price differ by style preference pattern***		
As the proportion preferring agroecology increased, total food production decreased and local food price increased; this effect on production and price decreased over time (Fig 2, top and bottom rows, respectively).	Compared to agroecology futures, entrepreneurial futures provided both more and cheaper food, especially in the near term.	Agroecology is slower to develop than entrepreneurial farming, requiring an initial transition period and with potential yield gains accumulating gradually over time. Each style sets their optimal production and asking prices via different processes.
***(ii) Global price transmission influences price but not production***		
As global price transmission increased, local food prices fell, with the difference between prices under lower and higher transmission increasing over time (Fig 2, bottom row). Transmission did not influence total food production (Fig 2, top row).	Farm gross income declined as price transmission (i.e. globalization) increased (because price fell but production was unchanged), and this had a cumulative effect over time.	The model assumes global food prices are (on average) falling and oscillating over time. This means that as price transmission increases there is greater downward pressure on local prices. This effect interacts with the aggregate asking price of local farmers.
***(iii) Farm income trajectories differ by farming style and over time***		
Patterns of farm development—using income slopes over the previous 10 years as an indicator—were uneven (Fig 3). Orphan farmers faced slow income decline at both 25 and 50 years (Fig 3, top row). Agroecology farmers had increasing incomes: rapidly at 25 years and more slowly at 50 years (Fig 3, middle row). Entrepreneurial farmers had increasing incomes at 25 years but falling incomes at 50 years (Fig 3, bottom row).	The results suggest that: in entrepreneurial futures, there would be initial progress amongst most converted farms, but the beginnings of a farming crisis would evident at 50 years; in agroecology futures, there would be rapid progress initially, with progress slowing after 50 years.	All orphan farmers will convert to their preferred style if their resources allow it under current price conditions. Thus, remaining orphan farmers have had their development blocked (at least temporarily). Agroecology incomes rise rapidly from a baseline of precarious subsistence. This slows over time as potential production rises and the style is established. For entrepreneurial farmers, their margin is dependent on input:output price ratios (i.e. off-farm conditions). Over time they face an increasing “squeeze” (falling food prices, rising input costs) ([e.g. as described in 32]).
***(iv) Fates of the worst-off households are mixed*, *with the pattern differing by style preference pattern***		
As the proportion preferring agroecology increased, the decline in the incomes of orphan farmers increased, with a greater effect at 50 years compared to 25 years (Fig 3, top row).	Some orphan farmers had their development blocked to a greater degree in agroecology futures compared to entrepreneurial futures. However, more orphan farmers were able to convert to their preferred style in agroecology futures (result not shown). That is, some orphan farmers were harmed while others benefited.	Opposing effects are operating. Agroecology benefits some orphan farmers, allowing them to convert as it has lower entry barriers (i.e. costs) than entrepreneurial farming, and it may generate conditions (i.e. higher prices) that allow others to convert. However, at the same time, the higher food prices also tended to trap the very worst-off farmers as they sell little food on the market but face rising input prices (which are linked to food prices).

^a^ This column discusses key mechanisms that shaped the patterns of interest but other processes and between-process interactions are also likely to have contributed.

Collectively, the findings in Table 5 suggest that farming futures must consider trade-offs between the quantity of food produced, its price, the development of farming communities, and the fate of the most precariously placed households. For instance, if actions were to be guided by a theory of undernutrition that suggested abundant (i.e. addressing availability), low priced (i.e. addressing access) food was the solution to hunger, then the results would suggest that futures in which a high proportion of households adopt entrepreneurial farming in a highly globalized market would be the preferred future (Fig 2). This suggestion is complicated, though, when the implications for rural communities are considered (Fig 3). Futures that may appear to offer the greatest food security are precisely futures in which the model suggests farming would be in crisis (i.e. incomes tend to be falling) after 50 years (Fig 3, bottom right panel). In contrast, agroecology-orientated futures appear to mitigate these negative effects, potentially sustaining rural communities, but production increases are slower and food prices higher, and the worst-off households may have their development blocked (Figs 2 and 3).

In other words, different farming futures—that is, different constellations of farming styles and their development trajectories—appear to have very different impacts on food production, price, and the fates of farms. This may in turn be expected to have significantly different impacts on the conditions supporting the health of farming households. Further, these impacts would be expected to be modified by both climate change and agricultural policy. These expectations were explored in the second set of simulations.

The ‘Climate change and agricultural policy’ runs showed four key patterns: (i) between-policy differences in production and price reversed over time; (ii) the ‘peasant’ policy had mixed effects on farm conversion and abandonment rates; (iii) different facets of the farm development process had different implications for nutrition; and, (iv) the farm development process shaped a range of conditions that may support or undermine rural health. Table 6 describes the relevant patterns, their implications, and provides comments on the underlying mechanisms.

**Table 6:** The four key patterns seen in the results of the ‘Climate change and agricultural policy’ model runs, their implications, and comments on the underlying mechanisms.

Pattern in the results	Implications	Comments on mechanisms^a^
***(i) Under climate change*, *between-policy differences in production and price reversed over time***.		
After about 30 years, production was highest under ‘peasant’ policy, with the gap between the latter and other policies increasing as climate change worsened (although total production simultaneously fell) (Fig 4, top row). In the absence of climate change and after 50 years, local food price was highest under ‘peasant’ policy, with prices tending to converge across all policies under low climate change and being lowest in the ‘peasant’ policy under high climate change. Prices were more stable under the ‘peasant’ policy.	‘Entrepreneurial’ policy initially provided the most food at the lowest prices. However, after an initial development period, food availability was highest under ‘peasant’ policy at prices that were similar to other policies under low climate change, and lower than under other policies under high climate change. Additionally, the tendency for more stable prices in ‘peasant’ futures may have reduced the risk entailed in agricultural livelihoods.	As described in Table 5, agroecology is slower to develop than entrepreneurial farming and each style sets its production and asking price differently. Additionally, in these runs, the introduction of supportive policies leads to faster rates of agroecology yield increments, which appears to allow farmers to sustain their livelihoods at lower asking prices than under the ‘entrepreneurial’ policy. Prices are more stable under ‘peasant’ policy as agroecology is relatively insulated from markets.
***(ii) For farm conversion and abandonment*, *mixed benefits and harms were evident under ‘peasant’ policy***		
The greatest number of farms were able to convert to their preferred style under ‘peasant’ policy (Fig 4, third row). However, more farmers abandoned farming under ‘peasant’ compared to ‘entrepreneurial’ policy (Fig 4, bottom row).	As described in Table 5, ‘peasant’ policy had opposing effects on orphan farmers, both facilitating conversion for some and blocking the development of the worst-off.	Lower transition costs allow more farmers to convert to agroecology earlier. The resulting higher food prices as agroecology proliferated, however, leads to prices of necessary inputs (which are linked to food price) that may be too high for farms with the lowest productive potential.
***(iii) Different facets of the farm development process had different implications for nutrition***		
Patterns of nutrition were influenced by total food production, blocked development, farm abandonment, and successful farm conversion (Table 4). Nutrition related to total production and conversion was highest under ‘peasant’ policy for all climate scenarios. However, under the same policy, nutrition related to blocked development was at its worst and nutrition related to abandonment was at similar levels to that seen under other policies.	These findings—particularly that under a given policy/climate combination different facets of the farm development process may either benefit or harm nutrition—underscore the need to look beyond food quantity and quality, and to specifically consider producer-consumers, in future climate-nutrition modelling.	On average, orphan farmers have limited production potential relative to reproduction requirements (i.e. production and consumption). Thus, if their development is blocked, many will have poor nutrition. Farmers decide to abandon their farms if they cannot provide at least half a basic diet to the family. For households that have been able to convert to their preferred style, their level of production far exceeds basic dietary requirements.
***(iv) The farm development process shaped conditions that may support or undermine rural health***		
Farm development trajectories shaped patterns of labour, farm income, income inequalities, and ‘real land productivity’ (Fig 5), each of which would be expected to influence community health in rural areas. All of these conditions were most supportive of rural health under ‘peasant’ policy under all the climate scenarios.	These findings suggest that future climate-nutrition models should consider not only how farm development trajectories impact on nutrition, but also how they may shape rural health more generally via impacts on conditions that are supportive of (or harmful to) community health.	On labour: agroecology develops via labour-intensification while entrepreneurial farming develops via capital intensification. On income: low input costs and the avoidance of debt contribute to relatively higher net incomes in agroecology. On income inequalities: more and smaller farms under ‘peasant’ relative to ‘entrepreneurial’ policy results in lower inequalities. On ‘real land productivity’: the use of off-farm produced inputs on entrepreneurial farms means proportionally less new value is generated on the farm than on agroecology farms.

^a^ This column discusses key mechanisms that shaped the pattern of interest but other processes and between-process interactions are also likely to have contributed.

In additional to Table 6, a number of further discussion points arise. The first relates to food production (Table 6, pattern (i)). Under the entrepreneurial policy, production initially rose rapidly but then began to decline after about 20 years before again rising, albeit slowly (Fig 4, top row). The decline is not explained by trends in farm conversions: the rate of conversion was slowing (Fig 4, third row) and this may have slowed production growth but it would not directly cause a decline. Nor is it explained by farm abandonment (Fig 4, bottom row). The farm income curves (Fig 5, fourth row), however, show the decline in production was a rational action aimed at maximising incomes. Immediately before the drop in production, income growth was slowing; during the subsequent period of falling production, however, income grew rapidly. That is, for entrepreneurial farming, the interactions of farmer goals, on-farm conditions (e.g. assets), and off-farm conditions (e.g. price ratios) may at times drive production downwards while simultaneously increasing net incomes.

A similar production pattern was not evident in the ‘peasant’ scenario (Fig 4, top row). Peasant farmers aim to increase returns per labour object (e.g. returns per hectare) and attempt to render themselves less sensitive to off-farm conditions. As a result, total production rose continuously over the model runs. Of further note, production at 50 years was lowest under ‘entrepreneurial eroding’ policy (Fig 4, top row): this highlights the risks faced by styles that are highly dependent on changeable off-farm conditions that are beyond their control.

The second point relates to the opposing effects of peasant-based agroecology on conversion and abandonment rates (Table 6, pattern (ii); see also Table 5, pattern (iv))). The potential for negative impacts on the worst-off households and the means of addressing them should perhaps be explored in future empirical work and incorporated into model. For instance, programmes that ensure the most precariously placed households are included in community knowledge networks which aim to strengthen peasant farming may be developed [51,65]. Of additional note, under the ‘none’ policy, in which entrepreneurial and agroecology styles coexist, conversions were slower and lower than under ‘peasant’ policy and there were the highest levels of abandonment. This suggests that the viability of a co-existence strategy, that may appear robust due to a mixture of both peasant and non-peasant farming, may actually be harmful; this should be investigated in future work.

The third point relates to the impacts on the health-supporting conditions (Table 6, pattern (iv)). We have suggested that the higher levels of farm labour under the ‘peasant’ policy compared to the ‘entrepreneurial’ policy are beneficial for health (Fig 5, second row). This, however, is contentious. Some argue that reduced farm labour releases people from undesirable toil to work in other sectors; others argue that agroecology generates rewarding work [84]. While this issue, along with its health-related implications, is likely to remain subject to dispute, two relevant considerations are: (i) the nature of work differs by farming style, meaning both positions may be correct: labour on entrepreneurial farms may entail drudgery while labour on peasant farms may be more rewarding [32,84]; and (ii) for people no longer working on farms, decent alternative employment in cities may not be available [85].

The results also show that income inequalities initially rose rapidly in all scenarios (Fig 5, third row). It may be speculated that, during this initial transition period, hardship for the many in the context of rising prosperity for a few may harm community health, and may have unexpected (and unmodelled in the ABM) influences on the longer-term development trajectories (e.g. via high levels of competition and rapid accumulation of land by the first to develop [39]).

Of final note on the results for the health-supporting conditions, both farm income and real land productivity are lowest under ‘entrepreneurial eroding’ policy (Fig 5, bottom two rows). This again shows the potential for farming styles that are heavily dependent on external conditions to place farming livelihoods in jeopardy, as well to farm less intensively (in the environmentally-sensitive sense of real land productivity), if these external supporting conditions are not maintained.

### Sensitivity analysis

The sensitivity analysis assessed the impacts on total production and price when the assumptions about maximum yields and climate sensitivities of agroecology and entrepreneurial farming were varied. There were three key findings.

Firstly, farmers tended to produce at a level closer to their maximum yield under the ‘peasant’ policy than under the ‘entrepreneurial’ policy (Fig 6, top row). For instance, in futures without climate change, when maximum production for agroecology was 50% lower that for entrepreneurial farming (yield ratio 1:2), total production under ‘peasant’ policy was just 25% lower than under ‘entrepreneurial’ policy.

Secondly, the between-style gap in actual compared to maximum production widened when climate change was introduced (Fig 6, top row, middle and right panels). This was seen regardless of whether it was assumed agroecology was more or less sensitive to yields losses due to climate change than entrepreneurial farming. For instance, when agroecology maximum production was 50% of that of entrepreneurial farming (yield ratio 1:2), total production for the ‘peasant’ policy was just 14% lower than for ‘entrepreneurial’ under low climate change; this gap closed to 8% under high climate change. For yields ratios ≥ 3:4, production in the ‘peasant’ scenario exceeded that in the ‘entrepreneurial’ scenario.

Together, these first two patterns show that the main results are not dependent on either agroecology having equal (or indeed, higher) productive potential to entrepreneurial farming or being less sensitive to climate change. Rather, it suggests that the between-style differences in the way production and consumption decisions are made—which rest on differences in underlying goals—play a key role in shaping the results. These between-style differences are not accounted for in previous climate-undernutrition modelling [e.g. 10,11–17,24,31].

The third finding relates to food price. Here, patterns by climate change scenario at 50 years (Fig 6, bottom row) are broadly similar to those seen in the main results (Fig 4, second row). In futures without climate change, food prices were considerably higher under the ‘peasant’ scenario compared to the ‘entrepreneurial’ scenario (Fig 6, bottom left panel). Of note, the influence of the agroecology to entrepreneurial yield ratio on the between-style price difference is minimal for ratios ≥ 1:2.

Under low climate change, prices tend to be 15% to 20% higher for ‘peasant’ compared to ‘entrepreneurial’ policy (Fig 6, bottom row, middle panel); in the main results, this price gap was smaller (Fig 4, second row, middle panel). Under high climate, ‘peasant’ policy prices were 10% to 20% lower than those for ‘entrepreneurial’ policy (Fig 6, bottom row, right panel); in the main results, this price gap was larger (Fig 4, second row, right panel). Once again, the yield ratio assumptions had little influence on the between-style price difference for ratios ≥1:2. Further, assumptions about between-style differences in sensitivity to climate change had only a small influence on the results.

When the production and price results are considered together, similar patterns are seen to those in the main results when the yield ratio is ≥3:4. In futures with climate change and a yield ratio of 1:2, production is slightly lower under ‘peasant’ policy compared to the ‘entrepreneurial’ policy, with slightly higher prices under low climate change but slightly lower prices under high climate change. That is, when agroecology is assumed to have 50% of the productive potential of entrepreneurial farming, future production and price are reasonably similar. When the yield ratio is 1:4, the outcomes differ significantly to those in the main results, but the available evidence suggests agroecology yields currently exceed this level [e.g. 51,56,57]. Additionally, it has been argued that ongoing research and on-farm knowledge generation has the potential to further increase agroecology yields over time [42] (We note that the sensitivity analysis assumes there are no yield increases over time for agroecology).

In sum, while the sensitivity analysis shows the yield- and climate change-related assumptions influence the results (as would be expected), they do not significantly alter the general patterns when held within plausible bounds. While we consider the tested parameters to be the most important, we recognise the model utilises many other parameters (Tables 1 and 2). It is possible that model output may be sensitive to one or more of these. Future work should further refine the parameters and assess model sensitivity to those that may have a strong influence on model output.

### Implications

Considered together, the upshot of the model results is that when attempting to understand how climate change may impact on future nutrition and health, patterns of farming styles—along with the fates of the households that practice them—matter. We stress that our model is not intended to directly represent the real world and we do not claim that the findings demonstrate that peasant farming and agroecology are the optimal ways forward. Rather, the model demonstrates that this may be a viable way forward, yet—despite being a future that is desired by many farmers [29]–it has been neglected in previous health impact modelling; thus, it warrants more attention.

Crucially, this line of inquiry is not just of academic interest: firstly, the contributions and vulnerabilities of peasants have been formally recognised by the United Nations with the adoption of the Declaration of the Rights of Peasants and Other Working People in Rural Areas (UNDROP) [86]; secondly, it goes to the heart of a current debate on the future farming. A recent report by The High Level Panel of Experts on Food Security and Nutrition [30] makes the distinction between ‘sustainable intensification and related approaches’ (which includes, for example, ‘climate smart agriculture’), and, ‘agroecological and related approaches’. In terms of our representations, the former is analogous to ‘entrepreneurial’ and the latter to ‘agroecology’. The report highlights, for instance, that sustainable intensification starts from the premise that ‘… productivity per land area needs to increase in a sustainable manner …’, while agroecological approaches emphasise ‘… reducing inputs and fostering diversity alongside social and political transformation focussed on improving ecological and human health …’, and that these two approaches ‘… are thus grounded in very different visions of the future of food systems’ [30].

Two distinct strands underlie this debate. The first is the empirical question of which futures are viable and would, for instance, be able to feed growing populations sustainably. The second is value-based: of these viable futures, which should we choose? [cf. 87] Shifting from a health impact model with a central focus on quantity and/or quality of food produced (i.e. where food is essentially considered to be ‘a thing’ that is separate from the processes that produced it) to one which explicitly considers farming styles (i.e. where food, how it is produced, and the social and environmental implications of this are considered together) simultaneously shifts from an approach that largely focusses on the empirical strand to one that includes aspects of the value-based strand. Both these strands are important for future population health, which include issues around who should choose the future we pursue as well as the distribution of benefits and harms.

### Model limitations

Our model has a number of limitations. The first relates to the representation of different farming styles. We drew on existing style-based categories [32,42,50] but simplified them to define agents that were rigidly distinct from one another. We accounted for differences in relations with the market, the type of farm inputs used, and goals, as these influence farmer decisions and behaviours. In the real world, however, there are additional differences and between-style distinctions are less rigid. Given this, it would be useful to develop more subtle representations in future work by: drawing on both theory and empirical analysis; combining insights from population health, rural sociology and farm household analysis; and, recognising the contested nature of how the food system should develop.

Two additional issues related to farming styles are: (i) we only allowed conversions from orphan to either entrepreneurial or agroecology farming; future models should allow for other between-style conversion (e.g. from entrepreneurial to agroecology); and (ii) the ABM does not represent the environmental impacts of farming (such as soil degradation and greenhouse gas emissions), which would be expected to differ by style.

A second limitation is model parameterization (Tables 1 and 2). We used approximations based on quantifications (e.g. yield loss per degree of warming [53,54]), ‘rules of thumb’ (e.g. production and consumption in orphan agriculture [50]), and qualitative knowledge (e.g. annual yield increments for peasant agriculture [42]). We argue, however, that given the nature of our model (a proof of concept model focussed on a hypothetical rural area) and its purposes (to assess patterns of outcomes and draw attention to previously neglected processes) our parameterization is a reasonable first-order approximation and is adequate to illustrate fundamental patterns. Future modelling should attempt to refine these parameters, partly using empirical research but also drawing on expert knowledge and opinion where gaps exist.

A key aspect of this is agroecology-related knowledge gaps. For Europe, modelling of an agroecology future found that while production would decline by 35% in 2050 compared to 2010 (from a starting point of highly productive agriculture), healthy food would still be available for all Europeans, export capacity would be maintained, and agricultural greenhouse gases would decline by 40% [88]. For regions with lower incomes, empirical work has shown considerable yields gains from agroecology and similar farming styles [e.g. 56,57,89]. However, this is an under-researched area, and some existing research conflates agroecology with other forms of sustainable intensification thus neglecting key aspects of agroecology such as greater farmer autonomy [51,65].

A third limitation is that the model represents only some aspects of the global food system. For instance, the model does not include a ‘demand-side’ (other than the demand of farming households) that influences production and prices. Instead, we assume prices are set by the supply-side and that all food for sale will be purchased. This was partly intentional because, as Gliessman [28] argues, conventional supply-demand models essentially see agriculture as ‘one giant farm’ and group all people together as homogenous ‘consumers’. Such a representation excludes factors that would be expected to impact on population health. Additionally, the ABM doesn’t consider, for example, value-chains and their effects on nutrition [e.g. 90], or dietary diversity and the environmental consequences of dietary patterns [e.g. 91]. We argue, however, that these limitations are justified as they are both necessary—no model can represent the entirety of a complex reality—and advantageous: they allow the exploration of a part of reality that has not only been neglected but may provide key insights to achieving healthy, sustainable futures.

A fourth limitation is that the climate (Table 1) and agricultural policy (Table 3) scenarios were represented simply. This was intentional as it renders our assumptions transparent, but it would be possible to, for example, use more detailed climate scenario data in future ABMs. Under our representation (Table 1), the results showed average yield losses under low and high climate change after 50 years (relative to no climate change) of 9% and 18% under ‘peasant’, and 20% and 32% under ‘entrepreneurial’ policy, respectively. Losses of this magnitude are at the upper end of warming-related yield declines found across crop models [92]. However, we argue that this is partly justified because our model is intended to represent populations who live in regions that are expected to be most impacted (i.e. tropical regions), and, our model attempts to account for the effects of droughts as well as warming trends. For agricultural policies (Table 3), additional entrepreneurial- and peasant-favouring measures and their expected benefit could be explored and introduced.

## Conclusions

By developing a model that views the climate-nutrition relation from the standpoint of farming styles, we have gained new insights. Firstly, along with food quantity and quality, patterns of farming styles are likely to have a strong influence on future nutrition. Secondly, farm development trajectories may have contradictory effects at a given time point (e.g. potentially benefiting some subsistence farmers but harming others) and on the same group at different times (e.g. rapidly rising production and falling prices may initially lead to rising incomes for some farmers but eventually to falling incomes). Finally, patterns of farming styles and their associated development trajectories will influence not only nutrition but also conditions that support rural health (e.g. labour, inequalities). We argue that each of these issues should be given greater prominence in debates amongst health-focussed researchers and in future modelling exercises. Specifically, we suggest a key strategy (in addition to, not in place of, existing strategies) for understanding the climate-nutrition relation is to move from a tendency seen in climate-health impact modelling to centre thinking around pathways traced from climate change to hunger, to instead focus on the development trajectories of farming styles and their implications for rural health (as well as population health more generally), and then ask how climate change may affect this.

In purely pragmatic terms, the question of how healthy, diverse diets could be provided for all people while living within planetary boundaries could be answered in multiple ways and achieved by various approaches to farming. Essentially, this is an empirical question. The question is complicated, however, by introducing issues such as democracy, justice, and equity, as these are normative issues that are contested [30], including in terms of what each of these actually entails. These latter issues are included as explicit goals of some styles of farming (e.g. agroecology [51]), and, different constellations of styles of farming are like to influence them in different ways. How this plays out in practice is of direct relevance to the global development agenda; for instance, agroecological practices “potentially contribute to 10 of 17 SDGs [Sustainable Development Goals] … and help address poverty and hunger, education, gender equality, decent work and economic growth, reduce inequalities, responsible consumption and production, climate action, life on land, and peace and justice.” [30].

Previous climate-nutrition modelling has tended to focus on the empirical aspects using quantitative modelling. We argue that in addition to the empirical aspects, the normative aspects, as well as their connections to the SDGs, should also be considered, including through building models with an explanatory focus (such as Agent-Based Modelling), as it is their combined effects that will ultimately shape patterns of health. With our model, we have attempted to take a first step in this direction; we suggest that future work should continue on this path.

## Supporting information

S1 Table Key assumptions and comments. (DOCX)

S1 Appendix ODD+D. (DOCX)
