# Supplementary material for: Climate change, hunger and rural health through the lens of farming styles: An agent-based model to assess the potential role of peasant farming
Source: PLoS One. 2021 Feb 11;16(2):e0246788. doi: 10.1371/journal.pone.0246788 (PMC7877765; doi:10.1371/journal.pone.0246788)
Supplement: S1 Table — (DOCX) [file pone.0246788.s001.docx]

**S1 Table. Key assumptions and comments.** This table outlines some key assumptions in regards to various aspects of the model and is intended as a complement to details given in the main paper and the accompanying ODD+D.

| **Aspect** | **Assumption and comments** |
| --- | --- |
| Why use **farming styles** instead of farm typologies? | We made the assumption that, given the purposes of the model, ‘farming styles’ provide a meaningful, albeit idealised, representation of real-world farmers. Farming styles are theoretically-derived empirically-based generalizations, derived from the extensive work of van der Ploeg and others in a many low-, middle-, and high-income countries [[1](#_ENREF_1)].  By their nature (i.e. as generalizations), representations of farming styles are not obtained by empirical investigation at a single, particular site. This latter approach is adopted when developing ‘farm typologies’ for models that attempt to represent particular real-world settings, and this is a useful approach for some purposes.  We, however, intentionally used farming styles approach precisely because of their potential applicability to many locations. In this regard, our approach is in line with comments made in a recent high quality review of farm household modelling: models “… should avoid the trap of developing complicated models for site-specific analysis that are difficult to apply to other sites because of data demands”) [[2](#_ENREF_2)]. (See the Introduction and Discussion sections of the main paper for further details). |
| What are the **style-specific differences** in relation to land, labour, and other resources? | We assumed that faming styles differ in relation to how they use land, labour, and other resources.  For labour: peasants use family labour and do not cost it when making production decisions; entrepreneurial farmers use wage labour which they may partly pay using credit). For land: a key goal of peasant farmers is to maximize returns to labour object (that is, per hectare) and will not expand until their most recently acquired plot has been successfully concerted to agroecology; entrepreneurial farmers do not focus on returns from a given hectare but rather on their overall margin). For other resources: peasants acquire few farm inputs on the market; entrepreneurial farmers acquire all their inputs from the market; and, peasants use their savings to make purchases whereas entrepreneurial farmers use credit. [More details are given in the main text, particularly in the ‘Agent’ section of ‘Model details’.] |
| In the model, what is **corporate agriculture**? | Corporate agriculture plays an active role in farm development through various mechanisms including land grabbing [[3](#_ENREF_3)]. In this model, however, we assume corporate agriculture is an external force (exogenously driven) that tends to put downward pressure on prices (although we assume global price oscillates and rises in response to warming and drought).  We made this assumption in order to focus on entrepreneurial and peasant farming as they represent two general routes via which farming may develop. This was because, firstly, either style may be able to produce sufficient food under climate change, but each style has potentially different implications for justice and equity. And, secondly, there are potentially harmful relations between entrepreneurial and peasant farming [[4](#_ENREF_4)]. Representing corporate agriculture in more detail would have made it difficult to focus on these aspects. |

**Table S1, continued**

| **Aspect** | **Assumption and comments** |
| --- | --- |
| In the model, what is **agroecology**? | In the real world, agroecology entails a varying range of on-farm practices and produces a diverse outputs on individual farms [[5](#_ENREF_5)]. In the model, however, we represent agroecology in a particular and simplified way: primarily as a means of enabling a peasant-style of farming to pursued and developed.  That is, in the model, agroecology is represented as a means of increasing yields while maintaining key aspects of the peasant-style; in contrast, market-orientated entrepreneurial style is not compatible with peasant goals. For this reason, we ‘black box’ actual on-farm practices in our model, but include many between-style differences in decision making and goals.  This approach is partly justified by views expressed by the High Level Panel of Experts on Food Security and Nutrition [[6](#_ENREF_6)]. ‘Agroecology’ is not viewed as simply another mode of on-farm practice; it also aims to foster social and political transformation: in relation to the model, this is represented by the peasant goal of ‘increased autonomy’ (actualized by reduced market dependence and avoidance of credit). In contrast, ‘sustainable intensification’ and ‘climate smart agriculture’ primarily aim to increase/maintain productivity per land area in a sustainable manner; this is more in line with the goals of entrepreneurial farming. Thus, in this formulation, as well as in the model, ‘agroecology’ is not compatible with entrepreneurial farming. (And in the real world, this reflects important distinctions between climate smart agriculture and agroecology; e.g. [[7](#_ENREF_7)]). |
| How were **maximum yields under agroecology** quantified? | For assumptions regarding average maximum yield potentials, as our model is stylized, we took a general approach. For orphan and entrepreneurial agriculture, we drew on Mazoyer & Roudart [[8](#_ENREF_8)], using averages of 1 tonne per ha and 10 tonnes per ha, respectively.  Determining an intermediate level of productivity for agroecology was more difficult. One reason is that, relative to conventional agriculture, or some other forms of sustainable intensification, agroecology is under researched. Relatedly, most review studies of yield improvements consider changes in production of single crops, whereas agroecology generally produces multiple crops. If production in terms of *total food* were considered, it has been argued that agroecology would be shown to be more productive than it appears in crop-by-crop comparisons. This may be done, for example, using the “land equivalent ratio” (LER) (which accounts for diversity): as an example, in Mexico, it was shown that 1.73 ha of *maize monoculture* had to be planted to produce as much *food* as 1 ha of *“milpa” (a combination of maize, beans, and squash)* [[9](#_ENREF_9)].  Additionally, review studies of yield improvements either group together a mix of approaches or look at single strategies for yield improvements. Thus, potential improvements of combined approaches may be underestimated. On the other hand, reviews that compare ‘organic’ agriculture (again, a broader category than agroecology) to conventional farming find yield differences are smaller: e.g. Seufert et al (2012) find that yield differences are highly contextual but range from around 5% to 34% lower for organic systems [[10](#_ENREF_10)].  Given this, we aimed to use an intermediate figure for the initial food productivity of agroecology relative to both orphan farming and entrepreneurial farming. We assumed that agroecology had, on average, 4/10 of the productive potential of entrepreneurial farming. On the one hand, this may appear optimistic in relation to review studies [[11](#_ENREF_11)], but on the other hand it may appear pessimistic in relation to studies comparing organic to conventional farming [[10](#_ENREF_10)]. Given this, we suggest our assumption is reasonable. Further, we have tested the implications of relative yield assumptions in the sensitivity analysis. |

**Table S1, continued**

| **Aspect** | **Assumption and comments** |
| --- | --- |
| Where did quantifications of **incremental yield gains in labour-intensive agriculture** come from? | We assume that in labour-intensive - i.e. peasant-based – farming, incremental yield gains may be made on (and only on) farms that are able to ‘optimize’ production. This is because via labour-intensive work, farmers may continually gain insights into which growth factors are the most limiting, and this enables them to gradually increasing yields [[12](#_ENREF_12)]. The available literature, however, made quantify these gains difficult.  Under scenarios *other than* ‘peasant policy’, it is assumed that optimized orphan farming results in yield increases of 1% per year, and optimized agroecology of 1.5% per year. ‘Optimize’ effectively means that farmers have sufficient income to purchase all the necessary inputs they require and are able to provide a sufficient labour diet. That is, no yield gains are achieved if the farm is struggling to survive as this suggests that little may be learned during the labour process. Additionally, maximum peasant yields are restricted such that they may not exceed maximum yields of entrepreneurial farming.  The above conditional gains are roughly in line with the empirically-derived global averages of 0.9% to 1.6% per year for the major crops as specified in a review paper [[13](#_ENREF_13)].  Under ‘peasant policy’ scenarios (in which it is assumed that support is directed towards peasant farming) it is assumed that yield gains on optimized orphan farms increase to 1.5% per year and on agroecology farms increase to 3% per year (Again, maximum yield may not exceed maximum yields of entrepreneurial farming.). While this is speculative, we suggest that our assumptions are reasonable given that above review paper estimates [[13](#_ENREF_13)] include all types of farming, many of which may undermine their own resource base and few are ecologically-based, and given that relatively few resources are currently used to research and support peasant agriculture compared to entrepreneurial style.  We assess the implications of our assumptions in the sensitivity analysis, where we test a range of different peasant:entrepreneurial yield ratios, and when doing so we assume there are *no incremental gains* (i.e. 0%/year) in peasant farming. In general, our conclusions remain qualitatively similar.  In sum, our yield gain assumptions are reasonably similar to averages reported in empirical reviews (e.g. Ray et al 2013), they only apply when farms aren’t struggling and it is plausible that farmers are learning during the labour process, and our results are generally qualitatively similar when we set yields gains = 0%. We believe this justifies our modelling choices. We would be able to add supplementary text to the manuscript outlining this. |

**S1 Table, continued**

| **Aspect** | **Assumption and comments** |
| --- | --- |
| How is **food price** modelled? | Local price is set endogenously based on the yield-weighted aggregate derived from the asking prices of individual households, albeit adjusted for exogenously set global prices (the influence of which varies by “global price transmission” scenario). The asking prices differ by household given each agent’s expected price (which is derived differently for each farming style and includes a random element to represent unmodelled processes) as well as style-specific goals (peasants aim to increase returns per labour object (i.e. per hectare), while entrepreneurial farmers aim to increase returns-on-investment and meet debt obligations. Given these style-specific differences, overall price trends are influenced by the proportion of farmers practicing each style.  Global price is set exogenously and influences local price via a scenario-dependent price transmission elasticity. On average, global price tends to fall and oscillate but rises in response to average warming trends and drought events (see also “In the model, what is corporate agriculture?” in this table). |
| How are **target yields** set? | In each year, target yields for each household have a maximum given their resources (farm size, technology, labour) but actual target yield is based on their style-specific goals and expected prices (see also “How is food price modelled” in this table). |
| Why use “**cereal equivalents**”? | We assume that farms produce homogenous ‘cereal equivalents’. In doing this, we do not intend to suggest all farms are actually producing cereals or monocultures. Rather, we use concept as a means of roughly representing total food production rather than tonnes of grain. That is, diversity of production, such as in real-world agroecological farms, is implicit. (Also see, “How were maximum yields under agroecology quantified?” in this table). |
| How do farms access **labour**? | There is no labour market in the model, nor do households arrange labour collectively. We make the simplifying assumptions that (i) for entrepreneurial farming labour is available if it is desired and can be afforded, and (ii) for agroecology that labour is limited to a maximum of two full time workers whose maximum work potential is a function of their nutritional status.  We base our labour modelling on stylized descriptions given by van der Ploeg [[1](#_ENREF_1),[4](#_ENREF_4),[12](#_ENREF_12)]. Peasant households are assumed to use only family labour and do not cost labour in their production decisions. However, labour capacity is dependent on calorie intake: that is, if the household cannot provide those doing the work with a full diet, the worker cannot work at full capacity. Entrepreneurial famers pay for all labour – even if it is family labour – and include labour costs in production decisions. |

**S1 Table, continued**

| **Aspect** | **Assumption and comments** |
| --- | --- |
| How is **consumption** (of food) represented? | Consumption is an essential part of the model: for quantifying hunger, for being to labour for a given amount of time, and for determining whether a farm is viable.  Households consume what they produce or what they obtain on the market by selling their produce. They consume in an attempt to meet basic calorie requirements, and peasants additionally consume to provide a labour diet adequate for labour power requirements. If households are not able to meet 50% of a basic diet (that is, they are starving) or meet a minimum labour diet, they abandon the farm.  For simplicity, we do not represent a market for food consumption. We obviate the need for this by assuming that farm gate and consumer prices are equal: thus, in effect, it makes no difference to the household budget whether consumption is of produced or purchased food. This is also made possible as we only focus on the consumption of calories (see “How is hunger represented” in this table).  In taking this approach, we have excluded dietary diversity and dietary change. These are complex issues: for instance, we argue that it is not adequate to simply assume that as a country becomes richer, people will “automatically” eat more meat etc (as is assumed in some models) as diet is determined by a wide range of factors. Of particular relevance in the farming styles approach is that a shift towards farms that produce a range of foods based on local cultures and preferences (this becomes more feasible when farms achieve more autonomy and less market dependence) may have a strong positive effect on the diets of surrounding communities: that is, dietary diversity and change would need to be endogenous to the model (not exogenously forced as, for example, GDP per capita is assumed to rise). We have not attempted to represent these complexities in our model. |
| Why isn’t **migration** included? | Our model does not account for migration. It is a complicated and potentially contentious issue, and we backgrounded it in order to allow an initial focus on farming styles.  If migration were to be included in the model, a key issue would be that patterns of farming styles would be expected to influence migration, perhaps strongly. For instance, if the uptake agroecology increased the viability of small farms, rural-urban migration may decrease: that is, at the same time as requiring more labour per hectare, agroecology may increase labour availability as rural livelihoods may become increasingly viable (for example, due to decent farm incomes) and attractive. This issue is linked to the discussions around the different nature of labour on peasant vs entrepreneurial farms (see Discussion in main paper), an aspect that to our knowledge is not considered in labour market models (i.e. in the latter, all farm labour is considered to be qualitatively the same).  Given this, it would not be appropriate to include exogenously forced migration dynamics in the model, but adding endogenously modified dynamics would be complex: thus, we have not included it in our model.  In the model, we assume that, in conditions under which peasant-based agroecology is able to provide a viable and relatively autonomous livelihood, that remaining in rural communities to farm is a reasonably attractive option relative to migrating to cities (which, at least at present, often offer only precarious livelihoods). In more entrepreneurial-orientated futures, capital intensive development means less labour is required, which partly off-sets the potential for shortages due to rural-urban migration and the arguably lower attractiveness of wage labour.  This assumption adds a caveat to our findings, but we argue that is does not detract from the essential insights they provide. |

**S1 Table, continued**

| **Aspect** | **Assumption and comments** |
| --- | --- |
| How is **hunger** represented? | ‘Hunger’ is ultimately a very complex outcome and it can be represented in various ways. We made assumptions in regards to two issues: what is hunger?, and, what causes hunger?  On ‘what is hunger?’, we make the simple assumption that it is a lack of calories (i.e. energy intake). Arguably the most commonly used metric when discussing hunger is the ‘proportion (or number) at risk of hunger’, which was developed by the Food and Agricultural Organization (FAO) [[14](#_ENREF_14),[15](#_ENREF_15)]. In nutritional-terms, this measure is entirely based on calories [[16](#_ENREF_16)]. Further, global-level climate-health models are typically driven by the modelled impacts of climate change on the major crops (e.g. rice, wheat, maize, soy, groundnut) and their associated calorie production [[17-19](#_ENREF_17)], but in some models this is then extrapolated to other food and nutrients [[20](#_ENREF_20)].  While our assumption misses important aspects of hunger, we suggest it is reasonable given that it is in line with much of the previous climate-nutrition literature; it allows us to include in the model the need for a labour-diet (in terms of energy requirements) to allow famers to work; and, it allows us to shift the *main cause of hunger* being investigated away from food quantity and quality to patterns of farming styles.  More specifically, and on ‘what cause hunger?’, a central issue in our model is that – as has been long recognised – hunger has many ‘upstream’ (i.e. non-nutritional) causes. Previous climate-health impact models essentially focus on changes in food quantity and quality in consumers. In contrast, the main purpose of our model is to show that upstream processes associated with farm development trajectories and patterns of farming styles (such as industrialization, de-/re-peasantization, and deactivation) are important but neglected ‘causes’ of hunger in previous health impact assessments.  Taken together, we have used a simple representation of hunger in order to better focus on previously unexplored causes of hunger. (Also see “How is consumption (of food) represent?” in this table). |
| What is **health**? | ‘Health’ is a deceptively difficult and contested concept. Here we specifically consider the assumptions underlying how we represent health in the model; that is, as five health-supporting conditions (basic nutrition, farm incomes, labour, income inequality, and real land productivity.  Krieger [[21](#_ENREF_21)] makes the distinction between *processes associated with disease mechanisms*, and *processes that shape disease distribution*, with the latter being (what should be) the main concern of population (as compared to individual) health. In our model, basic nutrition is associated with disease mechanisms, and the rest of the health-supporting conditions are associated with disease distribution. That is, together they give a more complete indication of population health than nutrition alone; in terms of the model, they give an indication of the viability of the rural community. Further, all of these outcomes are endogenously generated by model processes.  This may be contrasted with previous climate-nutrition modelling which have solely focussed on nutritional outcomes, and in which socioeconomic conditions are exogenously specified by a scenario. |

**S1 Table, continued**

| **Aspect** | **Assumption and comments** |
| --- | --- |
| What is the **utility of a stylized model**? | The question of whether a stylized, theoretical model is useful for telling us something useful about the real world depends on (at least) two things (i) the current state of knowledge, and (ii) the goals of the model.  On the current state of knowledge, which is discussed in more detail in the introduction and discussion sections of the main paper, previous climate-nutrition models have tended to focus on changes in food quantity and quality accessible to consumers [e.g. [17](#_ENREF_17),[18](#_ENREF_18),[20](#_ENREF_20)]. By design, this omits key qualities of producer-consumer farmers, who comprise a large proportion of the global population (~2 billion people [[22](#_ENREF_22)]), are at high risk of poverty and poor nutrition, but may also have the potential to play a key role in ensuring a healthy, sustainable future. This latter group has been a core focus within the farm household modelling tradition; this work has tended to use empirically-derived heterogenous farm typologies that are very location specific [[2](#_ENREF_2)].  A major gap in existing work is the question of how patterns of generalized farming styles, which aim to identify ‘patterns of coherence underlying … heterogeneity’ (see “Why use farming styles instead of farm typologies?” in this table) [[1](#_ENREF_1)], may influence future farm development trajectories and how this may in turn impact on nutrition and heath. This is an important question with real world significance, directly related to debates about the future of farming in relation to, for example, sustainable intensification and agroecology [[6](#_ENREF_6)].  Introducing farming styles in climate-nutrition modelling, however, brings many complexities in itself and would be expected to have influences on many associated processes (for instance, labour access and migration; see the associated entries for these in this table). Our model does not attempt to address this full complexity and instead focusses on the implications of selected core processes. We do this for two reasons.  Firstly, attempting to introduce the full complexity, which includes many issues with are contentious, risks losing focus on the central issue of interest. In any case, no model is able to capture the full complexity of the real-world: choices must always be made [[23](#_ENREF_23)].  Secondly, to our knowledge and in our experience, the climate-health community is not familiar with the concept of farming styles or peasant farming. Consequently, at this time, it seems preferable to highlight key processes that differ to those underlying previous climate-nutrition models.  Given all the above, the goals of our model (as outlined in the introduction of the main paper), are to introduce the concept of farming styles to the climate-health impact community (and other interested groups), illustrate how patterns of farming styles may impact on hunger and health, and stimulate both debate and future work on these neglected but crucial issues.  Consequently, at this time, a simplified model – albeit one that isn’t an oversimplification give the state of knowledge and the goals of the model - serves the important purpose of bringing these real-world issues into view, illustrating their potential importance, and spurring future work. The latter should begin introducing more complexity to assess questions and controversies raised by this model. |

**References**

1. van der Ploeg JD (2018) The New Peasantries: Rural Development in Times of Globalization. Oxon: Routledge.

2. van Wijk MT, Rufino MC, Enahoro D, Parsons D, Silvestri S, et al. (2014) Farm household models to analyse food security in a changing climate: A review. Global Food Security 3: 77-84.

3. McMichael P (2012) The land grab and corporate food regime restructuring. The Journal of Peasant Studies 39: 681-701.

4. van der Ploeg JD (2017) The importance of peasant agriculture: a forgotten truth. Wageningen: Wageningen University.

5. Gliessman SR (2015) Agroecology: The Ecology of Sustainable Food Systems. Boca Raton, Florida: CRC.

6. HLPE (2019) Agroecological and other innovative approaches for sustainable agriculture and food systems that enhance food security and nutrition. A report by the High Level Panel of Experts on Food Security and Nutrition of the Committee on World Food Security. Rome: HLPE.

7. Clapp J, Newell P, Brent ZW (2017) The global political economy of climate change, agriculture and food systems. The Journal of Peasant Studies: 1-9.

8. Mazoyer M, Roudart L (2006) A History of World Agriculture: from the Neolithic Age to the Current Crisis. London: Earthscan.

9. Rosset PM, Altieri MA (2017) Agroecology: Science and Politics. Rugby: Practical Action Publishing.

10. Seufert V, Ramankutty N, Foley JA (2012) Comparing the yields of organic and conventional agriculture. Nature 485: 229-232.

11. Pretty JN, Morison JIL, Hine RE (2003) Reducing food poverty by increasing agricultural sustainability in developing countries. Agriculture, Ecosystems & Environment 95: 217-234.

12. van der Ploeg JD (2013) Peasants and the Art of Farming: A Chayanovian Manifesto. Halifax: Fernwood Publications.

13. Ray DK, Mueller ND, West PC, Foley JA (2013) Yield Trends Are Insufficient to Double Global Crop Production by 2050. PLOS ONE 8: e66428.

14. FAO (2003) FAO methodology for the measurement of food deprivation. Rome: FAO.

15. FAO (2014) Revision of the methodology for the estimation of the Prevalence of Undernourishment. ROME: Food and Agricultural Organization.

16. Svedberg P (2000) Poverty and Undernutrition: Theory, Measurement, and Policy. Oxford: Oxford University Press.

17. Hasegawa T, Fujimori S, Shin Y, Tanaka A, Takahashi K, et al. (2015) Consequence of climate mitigation on the risk of hunger. Environmental Science & Technology 49: 7245-7253.

18. Lloyd SJ, Kovats RS, Chalabi Z (2011) Climate Change, Crop Yields, and Undernutrition: Development of a Model to Quantify the Impact of Climate Scenarios on Child Undernutrition. Environmental Health Perspectives 119: 1817-1823.

19. Nelson GC, Rosegrant MW, Palazzo A, Gray I, Ingersoll C, et al. (2010) Food security, farming, and climate change to 2050. Washington, DC: IFPRI.

20. Springmann M, Mason-D'Croz D, Robinson S, Garnett T, Godfray HCJ, et al. (2016) Global and regional health effects of future food production under climate change: a modelling study. The Lancet 387: 1937-1946.

21. Krieger N (2011) Epidemiology and the People's Health: Theory and Context. Oxford: Oxford University Press.

22. IFAD. Conference on New Directions for Smallholder Agriculture, 24-25 January 2011, Rome, IFAD HQ: Proceedings of the Conference; 2011; Rome. IFAD.

23. Levins R (1966) The Strategy of Model Building in Population Biology. American Scientist 54: 421-431.
