# Supplementary material for: Climate change, hunger and rural health through the lens of farming styles: An agent-based model to assess the potential role of peasant farming
Source: PLoS One. 2021 Feb 11;16(2):e0246788. doi: 10.1371/journal.pone.0246788 (PMC7877765; doi:10.1371/journal.pone.0246788)
Supplement: S1 Appendix — (DOCX) [file pone.0246788.s002.docx]

**S1 Appendix. ODD+D model documentation**

Accompanying the paper, **“Climate change, hunger and rural health through the lens of farming styles: an agent-based model to assess the potential role of peasant farming”**; Lloyd SJ, Chalabi Z.

Table of Contents

[1 Overview 2](#_Toc26890920)

[1.1 Purpose 2](#_Toc26890921)

[1.2 Entities, state variables, and scales 2](#_Toc26890922)

[1.3 Process overview and scheduling 4](#_Toc26890923)

[2 Design Concepts 6](#_Toc26890924)

[2.1 Theoretical and Empirical Background 6](#_Toc26890925)

[2.2 Individual Decision Making 8](#_Toc26890926)

[2.3 Learning 9](#_Toc26890927)

[2.4 Individual sensing 10](#_Toc26890928)

[2.5 Individual prediction 10](#_Toc26890929)

[2.6 Interaction 11](#_Toc26890930)

[2.7 Collectives 11](#_Toc26890931)

[2.8 Heterogeneity 11](#_Toc26890932)

[2.9 Stochasticity 12](#_Toc26890933)

[2.10 Observation 12](#_Toc26890934)

[3 Details 12](#_Toc26890935)

[3.1 Implementation details 12](#_Toc26890936)

[3.2 Initialization 12](#_Toc26890937)

[3.3 Input data 13](#_Toc26890938)

[3.4 Sub-models 13](#_Toc26890939)

[4 Tables, figures, and pseudo-code 13](#_Toc26890940)

[4.1 Tables 13](#_Toc26890941)

[4.2 Figures 17](#_Toc26890942)

[4.3 Pseudo-code 20](#_Toc26890943)

[5 REFERENCES 29](#_Toc26890944)

# Overview

## Purpose­­­­­­­­­­­­­­

### What is the purpose of the study?

The purpose of the model is to gain an understanding of *how* the development trajectories of constellations of producer-consumer farming households practicing different styles of farming *may affect* patterns of hunger and health in a farming community, *under* *scenarios for* climate change, agricultural policy, global price transmission, and style preference patterns, *in* a global food system in which food prices are tending to fall and oscillate.

The model represents a stylized farming community and aims to assess the potential importance of previously unexplored aspects of the relation between climate change and hunger. Previous climate-health impact models have focused on changes in food quantity and quality for consumers, as well as the impacts of dietary patterns on both consumer health and the environment. This ABM focusses on how patterns of farming styles – which differ in terms of farmer goals and degree of market dependence – may shape both nutrition and conditions that support the health of rural communities, and, it acts as a virtual lab for testing the implications of various scenarios.

The key outcomes assessed are total food production, local food price, household nutritional status, farm labour (number of full-time equivalent workers), income Gini (i.e. income inequalities), average net farm income (and its rate of change), and ‘real land productivity’ (an ecologically sensitive measure of farming intensity).

### For whom is the model designed?

The model was designed for researchers with an interest in climate change and health. The results may be of interest to decision-makers and stakeholders involved in and/or affected by debates and choices about the future of farming.

## Entities, state variables, and scales

### What kind of entities are in the model?

**Agents** are farming households practicing a given style of farming: ‘peasant’ style, with sub-types of ‘orphan’ farming (i.e. subsistence farming on one hectare using manual tools) and ‘agroecology’ (labour-intensive; largely using on-farm produced inputs; using the market as an outlet to sell surplus yields; using savings rather than credit to make purchases; has a key goal of maximising autonomy), or, ‘entrepreneurial-style’ (capital-intensive; dependent on the market to both purchase farm inputs and to sell yields; if needed, uses credit to finance purchases; a key goal of expansion) (van der Ploeg, 2018, Mazoyer and Roudart, 2006). There is also an exogenous forcing term for global corporate agriculture, which is represented as a tendency for global food prices to fall and oscillate^[[1]](#footnote-2)^. The **spatial units** are 1 hectare plots of farm land. The **environment** is represented as climate change, composed of a warming trend and change in drought risk, which affects crop yields.

### By what attributes (i.e. state variables and parameters) are these entities characterized?

**Farming households** are characterized by: a list of adjacent 1 hectare plot they occupy, with farm size influencing their productive potential; their current and preferred farming style; their farm equipment, which determines the amount of land one worker is able to farm: **manual tools** (e.g. a hoe, which allows one worker to farm 1 hectare), **working animals** (e.g. oxen, which allow one worker to farm up to 5 hectares), or a **small tractor** (which allows one worker to farm to 16 hectares)**;** their preferences for saving money and rationing spending, which together represent their preference for developing the farm vs. feeding the family a basic diet; family nutritional status, as the proportion of a basic diet consumed; current farm income as well as a list of their income in previous years; their expected food price in the coming year, given actual food prices in previous years and their style-specific goals; their optimum (given their style-specific goals), target (given their resources and preferences), and actual (given weather and random variation (the latter representing unmodelled factors)) yields; their asking food price (i.e. when selling their yield) given their actual yield and farming style-specific goals; and, short-, mid-, and long-term debt.

**Global corporate agriculture** is characterised by: current global food price; average rate of price decline; and, the amplitude and frequency of oscillation.

The **1 hectare plots** are characterized by: whether the plot is occupied by a farmer; the maximum yield of the plot in the absence of inputs (e.g. fertilizer); and, the potential yield multiple of the plot under agroecology following an **agroecology transition period**.

### What are the exogenous factors / drivers of the model?

**Climate change**, characterised as a warming trend which sets the temperature anomaly relative to year 0, and, an annual drought risk. Both the warming trend and annual drought risk increase as the climate scenario worsens. The temperature anomaly causes yield losses for farming households, and it (implicitly) causes the average rate of global price decline to slow. If a drought occurs in the farming community, households lose a proportion of their yields; if a drought affects corporate agriculture, global food price rises. The climate scenario is selected by the model user as: no, low, or high climate change.

**Agricultural policy** is selected by the user and favours one style of farming over another. ‘Entrepreneurial’ policy favours entrepreneurial farming by subsidising farm inputs and lowering interest rates on credit; ‘Entrepreneurial eroding’ policy is initially the same as the previous but support erodes over time; ‘Peasant’ policy favours peasant farming by stimulating research and supporting community networks, represented implicitly in the model as increased rates of yield increase for peasant farmers; ‘No’ policy which means there are no actions supporting any style of farming.

**Global price transmission** which determines the influence of global food price (i.e. associated with corporate agriculture) on local food price (i.e. the price faced by the community of farming households (agents)). This is a user selected elasticity (e.g. a setting of 0.5 would mean for each 1% rise in global food price, local price would rise by 0.5).

**Farming style preference pattern** which sets the proportion of farming households that prefer to develop via a given style. At model initialisation, all farmers are peasants practicing orphan farming and they may develop either via by maintaining a peasant style but adopting agroecology- or by adopting entrepreneurial-style. The preference pattern is user selected; for instance, preferences may be set such that 40% prefer agroecology and 60% prefer entrepreneurial.

**Interest rates** for short-, mid-, and long-term credit (used for purchases of farm inputs, farm equipment, and land, respectively) are set according to the agricultural policy scenario.

### If applicable, how is space included in the model?

The **landscape** is composed of 1 hectare farm plots. Each farming household initially occupies 1 hectare of land but may expand to unoccupied adjacent (in any direction, but the world does not ‘wrap around’) plots if they have the necessary resources to purchase and cultivate it. Each plot has a randomly assigned maximum productive potential. Climate change and global food price influence all plots equally (i.e. for the former it is assumed that climate change does not vary across space occupied by the farming community, and for the latter it is assumed that factors such as distance from the nearest market do not affect food price). The landscape is stylized and does not represent a real-world location.

### What are the temporal and spatial resolutions and extents of the model?

Each **time step** represents one year and each simulation runs for 50 years. Each **cell** represents 1 hectare of farmland in a 21 by 21 grid (i.e. 441 cells).

## Process overview and scheduling

### What entity does what, and in what order?

**In each year** (i.e. time step), **six processes** occur in the following order, with the entity undertaking the process shown in square brackets (where the ‘observer’ is the model controller^[[2]](#footnote-3)^). In processes involving farming households, the order in which each makes decisions and/or takes actions is randomly determined.

1. Set weather [observer]

Given the climate change scenario, **weather** conditions are updated and the expected impacts are calculated: the **temperature anomaly** is incremented and the associated yield losses are calculated; **drought** risk is incremented and whether droughts affecting the farming community and/or corporate agriculture occur is assessed, along with the associated expected **average yield losses** and **global price rise**, respectively.

1. Produce crops [all farming households]

All farming households attempt to produce their **target yield** (for setting of target yield, see ‘Consumption and production decisions’ ahead), with **actual yield** being a function of the temperature anomaly, drought (if occurred), and random variation (representing unmodelled factors). Following this, each household calculates their **asking price** (i.e. the selling price they aim for), given their target yield, actual yield, and style-specific goals.

Finally, assessment is made of (i) the potential **yield increments** in the coming year for peasant farmers, and (ii) whether farmers have **transitioned a plot to agroecology**, with the transition period being set by the user^[[3]](#footnote-4)^,^[[4]](#footnote-5)^.

1. Set food prices and other prices [observer]

Global and local food prices are set. **Global food price** is set such that: it tends to fall annually by a climate scenario-specific average amount (with actual change varying randomly around the average, including the possibility of a price rise); tends to oscillate with a given amplitude and period; and, is adjusted upwards if a drought affecting corporate agriculture has occurred (this adjustment is made according to a climate scenario-specific average amount, with actual rise being randomly determined).

**Local food price** is set by combining household asking prices to give a production-weighted average price (i.e. where each household’s asking price is given a weight according the quantity of food produced by that household), and this is then adjusted according to global food price and the user selected level of **global price transmission**. Local food price represents both farm-gate and consumer price, which are assumed to be equal.

The following prices, which are functions of local food price, are then set: **low-skilled wage** (i.e. the cost of a full-time farm worker), **necessary inputs** (i.e. expenditure that is necessary to allow a worker to be productive, e.g. clothes, tool repair, building maintenance), **‘fertilizer’** (which stands in for all non-necessary purchased inputs, such as herbicides and pesticides), **farm equipment** (working animals and small tractors), and **land price** (for a 1ha plot). Under the ‘Entrepreneurial eroding’ policy scenario, current **interest rates** are calculated^[[5]](#footnote-6)^.

1. Consider farming style change [households practicing ‘orphan’ farming]

Farmers practicing **orphan agriculture decide whether to change to their preferred style of farming** (In the current iteration of the model, the only permitted changes are from ‘orphan’ to either ‘agroecology’ or ‘entrepreneurial’). Orphan farmers with a **preference for agroecology** will remain peasants and convert if they have sufficient savings (i.e. they avoid credit) to cover additional input costs during the agroecology transition period. Orphan farmers with a **preference for entrepreneurial** farming will change style if, after providing a basic diet for the family, their income plus savings would be sufficient to employ a full-time worker; they will use **short-term credit** to purchase fertilizer if they require additional funds.

1. Consider expansion [households practicing ‘agroecology’ or ‘entrepreneurial’ style]

Before converting to their preferred style, all orphan households occupy 1 hectare of land and use manual tools. Following conversion, a household may gradually expand the farm by acquiring adjacent (in any direction) unoccupied plots. In addition, to farm newly acquired land, households may require additional farm equipment and/or labour inputs.

**Agroecology** farms: An additional unoccupied adjacent 1 hectare plot and/or working animals will be acquired if the costs can be covered by household savings (i.e. credit is avoided). A maximum of one new plot can be acquired in each time step, and farmers will not acquire a new plot until the most recently acquired plot has been transitioned to agroecology. Agroecology households may have up to 10 hectares of land and two pairs of working animals as such a farm can be managed using family labour.

**Entrepreneurial** farms: An additional unoccupied adjacent 1 hectare plot and/or working animals or a small tractor may be acquired if farm income and savings cover half the cost; credit will be used to cover the remainder, if required. A maximum of one new plot can be acquired in each time step.

1. Allocate resources to consumption and production [all farming households]

All farming households make decisions about consumption (of food) and production, given their resources and style-specific goals. All households estimate their **expected food price** in the coming year, and then find their **optimum production level**.

**Peasant households** initially optimize given their farm land and equipment but without considering other resource constraints. Standard optimization methods are used except that farmers, who use family labour, do not cost labour when optimizing (i.e. they **maximise returns-to-labour**) (van der Ploeg, 2013). Instead, they must provide workers with a labour diet sufficient to allow them to produce at the optimal level (Strauss, 1986). Households then set their **target yield** at this optimal level unless (i) their resources do not permit this level production in which case they ration their resources across consumption and production, and will **abandon** the farm if, after selling farm assets, they are unable to meet at least 50% of a basic diet for the family; or (ii) optimal production would not provide an income sufficient to reproduce the farm and meet their autonomy-related goal of increasing value added per labour object, in which case they attempt to increase production.

**Entrepreneurial households** optimize, again using standard methods except that they aim to **maximise returns-on-investment** (van der Ploeg, 2013), given their resources (including the available credit). If at this optimal level returns-on-investment are negative (i.e. the farm would run at a loss), they sell an asset and re-optimize; if they have no assets to sell, they **abandon** the farm. They then assess whether income at optimal production would cover their expected expenses (e.g. debt obligations) and meet their income target: if not, they aim to produce at a level that would come as close to achieving this as possible; if, however, at this level returns-on-investment are negative, they sell an asset and re-optimize, unless they have no assets to sell, in which case they **abandon** the farm.

# Design Concepts

## Theoretical and Empirical Background

### Which general concepts, theories or hypotheses are underlying the model’s design at the system level or at the level(s) of the submodel(s) (apart from the decision model)?

The **general theory** underlying the model is that the root causes of hunger lie in patterns of poverty and inequality, and that these are partly generated by the food system itself; that is, the food system produces both wealth and poverty, and both good nutrition and hunger (Holt-Gimenez and Patel, 2009, Moore Lappe and Collins, 2015, Rossett, 2006, Buttel, 2000). **Two specific aspects of this are explored in the model**. Firstly, over-productive ‘corporate’ agriculture drives a tendency for food price to fall, which in turn reduces the viability of livelihoods for the least productive agricultures, pushing them into poverty and hunger (Mazoyer and Roudart, 2006). Secondly, policies that generally support ‘entrepreneurial’ farming tend to harm ‘peasant’ farming and may be generating a constellation of farms that is not sustainable and is highly vulnerable to changing institutional and market conditions (van der Ploeg, 2017).

The key **concept** employed in the model is **‘farming styles’** (van der Ploeg, 2018). While farms may differ by size and farmers may differ in terms of, for example, risk averseness, van der Ploeg (2018) suggests that, alongside these quantitative differences, there are also crucial qualitative distinctions. A key distinction is between ‘peasant’ (represented in the model as ‘orphan’ farming and ‘agroecology’) and ‘entrepreneurial’ styles of farming^[[6]](#footnote-7)^. Here, some key differences lie in (i) farmer goals, (ii) the means employed to intensify farming, and (iii) the way farms are connected to markets. **Peasant farmers** have a key goal of deepening autonomy. Means of achieving this include avoiding market dependence and increasing value added per labour object. Production is increased via labour-intensification. The farm production process produces food as well as most farm inputs: this means the next round of production is guaranteed^[[7]](#footnote-8)^ without recourse to markets (notwithstanding some necessary inputs that must be purchased, including as clothing, tool repairs, or for building maintenance). Peasant farmers also avoid credit, making purchases using their savings. Of note, peasants do not isolate themselves from markets: the market is used as an outlet for surplus production.

**Entrepreneurial farmers** have a key goal of expansion, and production is increased via capital-intensification which is generally partly financed using credit. The farm production process relies on purchased inputs (e.g. fertilizers) and employed workers, again often financed using credit. Yield is almost entirely sold on the market. This means the logic driving production decisions is largely shaped by off-farm processes, such as price ratios (determining the margin) and technology (determining scale); thus, the market acts as an ordering principle, and another goal of entrepreneurial farming is to maximise returns-on-investment.

**Corporate agriculture** has a central goal of maximising profit. In the ABM, however, corporate farms are not explicitly represented; rather, they are represented implicitly as a tendency for global food price to fall and oscillate.

The **hypothesis** of the model is that the development trajectories of different constellations of farming styles will have implications for both nutrition and the conditions supporting the health of rural communities, and that climate change, agricultural policy and global price transmission will modify these implications.

### On what assumptions is/are the agents’ decision model(s) based

Decisions on **optimal production** are assumed to be consistent with standard economic approaches (i.e. using a production function and a total factor cost curve) (Debertin, 2012, Ellis, 1993) but modified using van der Ploeg’s (2013) Chayanovian-based approach, in which goals differ by farming style (see above).

Decisions on **asking price** (i.e. for food to be sold in the market), on whether to **convert to the preferred style of farming**, and on whether to **expand the farm or purchase equipment**, are assumed to be taken in line with farming style-specific goals (van der Ploeg, 2013, van der Ploeg, 2018). For instance, for the latter, peasant farmers will only use saving to make purchases while entrepreneurial farmers will use credit (if needed).

Decisions on **rationing resources** (between nutrition and production) are guided by arbitrarily assigned fixed preferences, with the exception that it is assumed that nutrition will be favoured over production when faced with starvation (De La O Campos et al., 2018). For peasants, the decision to **abandon a farm** is made when nutrition falls below a threshold for survival, which is set to consumption of half a basic diet, on the assumption that when faced with ‘ultra hunger’ (De La O Campos et al., 2018) farming is no longer viable. For entrepreneurial farmers, it is assumed that the farm will be abandoned when it is running at loss or debt obligations cannot be met.

### Why is a/are certain decision model(s) chosen?

The decision models of central importance to the model are based on the empirically grounded theories on **farming styles** developed by van der Ploeg (van der Ploeg, 2013, van der Ploeg, 2016, van der Ploeg, 2017, van der Ploeg, 2018). These where chosen for the following related reasons.

Firstly, producer-consumer farming households comprise a large proportion of those affected by, and at risk of, poverty and hunger (IFAD, 2011), yet it has been argued this same group could hold the key to feeding populations healthily, mitigating climate change (and other environmental damages), and providing decent rural livelihoods (La Via Campesina, 2019, HLPE, 2019). In previous global-level climate-undernutrition models, however, production and consumption are separated by design, and both producer and consumers are represented essentially homogenously: production is not distinguished qualitatively by farming style, and, all people are cast as homogenous consumers (i.e. producer-consumers are not represented) (e.g. Lloyd et al., 2011).

Secondly, between-style distinctions go to the heart of debates on the future of farming. The High Level Panel of Experts on Food Security (HLPE, 2019) distinguish between ‘sustainable intensification and related approaches’ (SI) (which includes, for example, ‘climate smart agriculture’), and, ‘agroecological and related approaches’ . The former is analogous to ‘entrepreneurial’ farming and the latter to ‘agroecology’. The HLPE crucially notes that these two approaches are ‘… grounded in very different visions of the future of food systems’ (HLPE, 2019).

Thus, the model employs decision models based on theories of farming styles in order to assess the health implications of these possible future food systems under climate change.

### If the model/a submodel (e.g. the decision model) is based on empirical data, where does the data come from?

The model does not draw on explicit empirical data. Rather, it uses generalizations based on published empirically-based studies where possible.

### At which level of aggregation were the data available?

Not applicable.

## Individual Decision Making

### What are the subjects and objects of decision-making? On which level of aggregation is decision-making modelled? Are multiple levels of decision making included?

The decision-making subjects are farming households. The objects of decisions are: target production, whether to convert to their preferred farming style, whether to expand the farm and/or purchase new equipment, how to ration (if required) resources between consumption and production, whether to liquid assets if additional funds are needed, and whether to abandon the farm.

All decisions are made at the household level.

### What is the basic rationality behind agents’ decision-making in the model? Do agents pursue an explicit objective or have other success criteria?

Households find their optimal production according to their objectives (i.e. goals), which differ by farming style. Decisions on target production, as well as farm expansion, are made given resource constraints, style-specific goals, fixed preferences (assigned at model initialization), and thresholds (e.g. for abandoning the farm).

### How do agents make their decisions?

Decisions are made according to the rules represented in the decision trees (Figures A, B and C). Within these decision trees, decisions on optimal production are initially made using modified standard economic methods; i.e. based on production functions and cost curves (Debertin, 2012, Ellis, 1993) (see 4.3.6.3).

### Do the agents adapt their behaviour to changing endogenous and exogenous state variables? And if yes, how?

No.

### Do social norms or cultural values play a role in the decision-making process?

Not explicitly. However, the goals of peasant farmers arise from underlying peasant norms and values (van der Ploeg, 2018).

### Do spatial aspects play a role in the decision process?

When attempting to expand the farm, households may only acquire unoccupied adjacent plots (in any direction, but the world does not ‘wrap around’). If no adjacent plots are unoccupied, they cannot expand.

### Do temporal aspects play a role in the decision process?

When estimating expected food price in the coming year, agents consider the price trend over the previous 5 years. When making decisions guided by income-related goals, households base decisions on their average net income over the previous 5 years. When making decisions about production, agents account for previous yield losses due to climate change-associated warming trends.

### To which extent and how is uncertainty included in the agents’ decision rules?

No information that agents obtain (e.g. food prices) contains uncertainty. When agents are estimating their expected food price in the coming year, a random element is used to represent unmodelled factors which partly represent farmer uncertainty regarding future prices.

## Learning

### Is individual learning included in the decision process? How do individuals change their decision rules over time as consequence of their experience?

No

### Is collective learning implemented in the model?

No

## Individual sensing

### What endogenous and exogenous state variables are individuals assumed to sense and consider in their decisions? Is the sensing process erroneous?

Agents sense and use the following variables in their decision making: local food price and other prices (low-skilled wage, necessary inputs, fertilizer, working animals, small tractors, land, interest rates), warming trend-associated yield losses, the productive potential of a plot they are considering purchasing. Sensing processes are not erroneous.

### What state variables of which other individuals can an individual perceive? Is the sensing process erroneous?

None

### What is the spatial scale of sensing?

All sensed variables are sensed globally, except the productivity of plots being considered for purchase, for which only plots adjacent to the farm may be sensed.

### Are the mechanisms by which agents obtain information modelled explicitly, or are individuals simply assumed to know these variables?

Agents are assumed to know.

### Are costs for cognition and costs for gathering information included in the model?

No

## Individual prediction

### Which data do the agents use to predict future conditions?

They predict their expected food price in the coming year based on the local price in the previous five years. They predict their maximum possible yield in the coming year based on the current temperature anomaly (i.e. due to climate change).

### What internal models are agents assumed to use to estimate future conditions or consequences of their decisions?

An agent’s expected food price is based on a combination of: current local price and the price trend over the previous five years, random variation to reflect unmodelled factors, and their style-specific goals.

For predictions of temperature anomaly-associated yield losses, all agents are assumed to know the current anomaly and the associated yield losses.

### Might agents be erroneous in the prediction process, and how is it implemented?

The actual local food price in the coming year arises from prices and expectations of all agents; thus, predictions of individual agents are likely to be erroneous. All households face the same local food price, regardless of the expectations or initial asking price.

Temperature anomaly-associated yield loss predictions are not erroneous. However, actual yields for each household are subject to random variation (to capture unmodelled factors, which implicitly includes, for example, growing season temperatures that diverge from the anomaly-associated average)).

## Interaction

### Are interactions among agents and entities assumed as direct or indirect?

Households interact indirectly through local food price (which is determined by the production and expectations of all agents).

Households with farms located close to each other may also interact indirectly when purchasing additional plots. Households purchase the adjacent plot with highest productive potential, and once a plot is occupied it is unavailable to other households. Entrepreneurial farmers may expand faster than those practicing agroecology as the latter will not purchase additional land until the previously purchased plot has been transitioned (to agroecology).

### On what do the interactions depend?

Interaction via local food price depends on relative farm productivities; i.e. those with the highest production have the greatest influence on local food price, which is a production-weighted average.

Interactions via land purchase depend on spatial proximity of farms.

### If the interactions involve communication, how are such communications represented?

Not applicable.

### If a coordination network exists, how does it affect the agent behaviour? Is the structure of the network imposed or emergent?

Not applicable.

## Collectives

### If a coordination network exists, how does it affect the agent behaviour? Is the structure of the network imposed or emergent?

Not applicable.

### How are collectives represented?

Not applicable.

## Heterogeneity

### Are the agents heterogeneous? If yes, which state variables and/or processes differ between the agents?

Between-farming style heterogeneity is of central concern in the model. Peasant (orphan and agroecology) and entrepreneurial farmers are heterogenous; for details see 1.3.1 and 2.1.1, Figures B and C, section 4.3.

### Are the agents heterogeneous in their decision-making? If yes, which decision models or decision objects differ between the agents?

Peasant (orphan and agroecology) and entrepreneurial farmers are heterogenous in the decision making for: decisions on style change (i.e. depending on preferred style of the orphan farmer) and expansion (see 4.3.4 and 4.3.5), and decisions on allocation of resources to consumption and production (see Figures B and C) including when optimizing , setting target production and whether to abandon the farm (see Figures B and C, and 4.3.6.3), as well as when deciding their asking price (see 4.3.2.2).

## Stochasticity

### What processes (including initialization) are modelled by assuming they are random or partly random?

At model initiation: households are placed on randomly selected one hectare plots; the maximum potential yield and the agroecology yield multiple of each plot is randomly assigned (Table A); household preferences for the use of savings (use all, use half, don’t use, save additional 10% of income) and rationing (favour production, favour consumption, favour both equally) are randomly distributed (but in fixed proportions), as are style preferences (in user specified proportions).

In each time step: droughts occur randomly given their risk, and average expected yield loss (Table A) and rises in global food price are randomly set (Table B); yields have a random component to represent unmodelled factors (varying by about $\pm$15%; normally distributed, mean = 0, std dev = 6.5); global food price has a general tendency to fall, but actual change is partly randomly determined (and included the possibility of a rise); if local or global food price fall below 5c/kg they are increased by a random amount (see 4.3.3.1 and 4.3.3.2); household expected food prices in the coming year contain a random component to reflect unmodelled processes (see 4.3.6.3).

## Observation

### What data are collected from the ABM for testing, understanding, and analysing it, and how and when are they collected?

The key model outputs are described in 4.3.7. All data are collated at the end of each time step.

### What key results, outputs or characteristics of the model are emerging from the individuals?

The above outputs are assessed at the system level but they are not strictly ‘emergent’ (e.g. they are sums or aggregates of individual-level variables), but collectively they give an indication of the productive potential and health of the community as a whole.

# Details

## Implementation details

### How has the model been implemented?

Netlogo 6.0.1 (Wilensky, 1999).

### Is the model accessible and if so where?

On request from the author.

## Initialization

### What is the initial state of the model world, i.e. at time t=0 of a simulation run?

See Tables A and B.

### Is initialization always the same, or is it allowed to vary among simulations?

Households are randomly located. Style preference patterns are user selected.

### Are the initial values chosen arbitrarily or based on data?

Initial values are derived for the empirically-based literature where possible (see Tables A and B).

## Input data

### Does the model use input from external sources such as data files or other models to represent processes that change over time?

No

## Sub-models

### What, in detail, are the submodels that represent the processes listed in ‘Process overview and scheduling’?

For model a description of model variables and parameters, their initial values, and how they change over time, see Tables A and B. For decision trees, see 4.2. For pseudo-code, see 4.3.

### What are the model parameters, their dimensions and reference values?

See Tables A and B.

### How were sub-models designed or chosen, and how were they parameterized and then tested?

The model sub-models for farming household decisions were designed as expressions of van der Ploeg’s (2018) empirically-based theories of style-specific goals and behaviours, drawing on standard economic methods (Debertin, 2012, Ellis, 1993) but modifying them as required (Holt-Gimenez, 2019, van der Ploeg, 2013), empirical studies of farm productivity (Pimentel and Pimentel, 2008), ‘rules of thumb’ (for productive potential given equipment and land) (Mazoyer and Roudart, 2006). Additionally, *ad hoc* decision trees were developed, intended to represent style-specific goals and plausible farmer behaviour when faced with starvation or a farm that is running at a loss.

The climate sub-model was designed to be an approximation of possible changes in climate and weather and the possible impacts on farming.

The sub-models were test iteratively, which each section of code being tested (e.g. by tracking individual working variables), de-bugged, and modified as necessary.

# Tables, figures, and pseudo-code

## Tables

The tables in this section describe model variables and parameters, their functions, their initial values, and how they change over time. Table A shows factors associated with agents and the environment; Table B shows the prices of factors that may be purchased.

Table A. Key environment and agent factors, their initial values, and how they change over time

| **Factor** | **Function or effect** |  | **Initial value** | **Change over time** | **Notes** |
| --- | --- | --- | --- | --- | --- |
| **Landscape** |  |  |  |  |  |
| ‘Local area’ | Grid of 1 ha plots. |  | 441 plots. | No change. | A 21 by 21 grid of arable plots. |
| Plot max productivity | Each plot has a maximum productivity under orphan agriculture (i.e. in which no non-labour farm inputs are used). |  | Randomly set for each plot: 1000kg/year $\pm$20% (uniform distribution).  [Based on Mazoyer and Roudart (2006)] | Gradual increase on ‘optimized’ peasant farms. ‘Peasant’ policy: orphan 1.5%/year, Agroecol 3% per year; other policies: Orphan 1%/year, Agroecol 1.5%/year. Max production = 10t/ha. [Based on van der Ploeg (2013)] | ‘Optimized’ in terms of production; assumed that if farmer unable to optimize, then also unable to gain production increases.  Assumes no land degradation under any style. |
| Plot agroecology yield multiple | Max productivity of a plot is raised by a given multiple after transitioning to agroecology. |  | Randomly set for each plot: mean=4, SD=1.5 (normal distribution, restricted to values between 2 and 7).  [Based on Pretty et al. (2003), Pretty et al. (2018), Rosset and Altieri (2017)] | No change. | Productivity rises slowly during the transition phase, with the full yield multiple being achieved after the agroecology transition period (see 4.3.2.4) |
| Agroecology transition period | Number of years to transition a plot to agroecology. |  | 3 years. [Based on Rosset and Altieri (2017)] | No change. | Transition achieved via labour intensification (see ‘Agroecology labour multiple’) |
| **Agents** |  |  |  |  |  |
| Farming households | Farming households, each of four people, practicing a particular style of farming. Using manual tools, each household can farm one hectare. |  | 250; each randomly assigned a 1ha plot; all practicing orphan agriculture; preference to develop via a particular style distributed according to scenario. All households are assumed to have consumed a basic diet, be aiming to produce their maximum yield, and have no savings | Households change to preferred style if they have access to sufficient resources (see 4.3.4), or, abandon farming if nutrition falls below 50% of a basic diet. | Initially ~40% of plots are unoccupied. Approximates conditions in lower income countries. (Bruinsma, 2003, Mazoyer and Roudart, 2006, World Bank, 2019) |
| Family basic diet | Quantity of cereal equivalents providing a basic diet to a family for one year. |  | 700kg/year (equiv. to ~2200kcal/person/day). [Based on Mazoyer and Roudart (2006)] | No change. Households abandon their farm if they are unable to obtain 50% of a basic diet. | Household members do not age over time. |
| Labour diet | Worker calorie intake/day to allow a given amount of labour power. |  | 5100kcal/day for max production on 1ha; diminishing returns as intake increases to this level (see 4.3.6.1) [Based on Strauss (1986) & Pimentel and Pimentel (2008)] | Acquiring working animals or a small tractor allows a worker to farm more than 1ha (Table B). Labour input requirements double under agroecology. | For orphan agriculture, max production on 1ha with manual tools requires 150 ten hour labour days/year. [Based on Pimentel and Pimentel (2008)] |
| Agroecology labour multiple | Increase in labour requirements for maximum production in agroecology. |  | 2 (i.e. for max production, required labour time doubles). [Based on Rosset and Altieri (2017)] | No change. | ‘Necessary input’ requirements rise proportionally with labour (Table B). |

*Table A, continued*

| **Factor** | **Function or effect** | **Initial value** | **Change over time** | **Notes** |
| --- | --- | --- | --- | --- |
| **Climate** |  |  |  |  |
| Warming trend and yield losses | Yields decline as warming increases, with lower losses for agroecology.  (For effects on global food price, see Table B). | Warming = 0.  Yield loss = 4%/degree of warming [Based on Moore et al. (2017) and Zhao et al. (2017)]; losses reduced by 10% under agroecology. [Based on Rosset and Altieri (2017)] | Linear rise in warming.  High CC: 2 degrees/50years (i.e. 0.04 degrees per year); Low CC: 1 degree/50 years (i.e. 0.02 degrees per year); No CC: no warming. [Based on Knutti and Sedláček (2012)] | An approximation guided by average warming under the Representative Concentration Pathways (Moss et al., 2010). Agroecology loss reductions are an approximation. |
| Drought risk and yield losses | Proportion of yield lost if a drought occurs; lower losses under agroecology.  (For effects on global food price, see Table B). | Drought risk = 5%/year  Drought yield losses are -  High CC: av. 15%, up to 30%; Low CC: av. 10%, up to 25%; No CC: av. 7.5%, up to 20%. Losses reduced by 20% under agroecology (see 4.3.1.1 and 4.3.2.1). [Based on Rosset and Altieri (2017)] | Linear increase in risk –  High CC: doubles after 50 years; Low CC: 1.5 times after 50 years; No CC: no change.  Yield losses are fixed over time. | Drought losses are contingent on multiple processes meaning no generally applicable quantification available. Plausible approximations used, including for agroecology. |

Table B. Prices for key factors, their initial values, and how they change over time

| **Factor** | **Function or effect** | **Initial value** | **Change over time** | **Notes** |
| --- | --- | --- | --- | --- |
| **Food price** |  |  |  |  |
| Local food price | Food price faced by farming households. | 40c/kg (Given input prices (see below), this places the average farmer close to the threshold for development.) | Calculated as the production-weighted average of farmer asking prices, adjusted for global price given price transmission (see 4.3.3.2). | Farm gate and consumer prices assumed to be the same. |
| Global food price | Represents price arising from global corporate agriculture: influences trend in local price via global price transmission (Figure A) | 40c/kg | General tendency to fall (most rapidly under ‘no climate change’ and most slowly under ‘high climate change’ (due to warming)) & oscillate. Drought causes price increases, with the greatest increases under ‘high climate change’ (see 4.3.3.1). | The simulations aim to assess the impact of the tendency for global prices to fall and oscillate on smallholder farming. [Based on Mazoyer and Roudart (2006)] |
| **Inputs** |  |  |  |  |
| Labour: low skilled wage | Cost of a full-time farm worker (Labour time may be purchased in fractions given target yield). | Price = 180% of the cost of a basic diet for a family of four; i.e. price = 700kg * local food price * 1.8. [e.g. Wage Indicator Foundation (2019)] | Same formula (based on average local price over last 5 years), but with an additional rise of 2% per year [Based on ILO (2016)]. | Peasants do not cost labour. Over time, food costs represent a smaller proportion of people’s income. |
| Purchased inputs: necessary inputs | ‘Necessary inputs’ represent expenditure required to enable production. Assumed to be scalable given target production. | Necessary inputs for max production: price/ha = 15% of a low skilled wage.  [Based on Petersen and Silveira (2017) and van der Ploeg (2016)] | Under agroecology, necessary inputs for maximum production double (i.e. in proportion to increased labour requirements (Table A)). | Necessary inputs include clothing, tool repair, building maintenance, etc (Mazoyer and Roudart, 2006). |
| Purchased inputs: fertilizer | Increases productivity of a plot up to 10 times (Mazoyer and Roudart, 2006), with diminishing returns as quantity used increases to max (see 4.3.6.2) | Price of 1kg = local food price/kg * 10. Max productivity at 500kg [e.g. (Roser and Ritchie (2019), van der Velde et al., 2013, Yamano and Arai, 2011)]. Under ‘Entre’ and ‘entre eroding’ policy: 50% subsidy. | Same formula, but price rises 1%/year.  Under ‘entre eroding’, subsidy falls by 1%/year. | ‘Fertilizer’ assumed to represent all non-necessary purchased inputs (e.g. pesticides, seeds). Thus, the fertilizer:food price ratio accounts for this. |
| Working (i.e. draught) animals | Allows one worker to farm up to 5ha (cf. manual tools, which allow 1ha to be farmed). | Price = 30 years of net income (i.e. after feeding the family) of average orphan ag farm (See 4.3.3.3). [Based on Mazoyer and Roudart (2006)] | Same formula, based on average local food price over the last five years. | Working animals allow workers to farm a greater area but do not increase plot productivity. |
| Small tractor | Allows one worker to farm up to 16 hectares (cf. manual tools, which allow 1ha to be farmed). | Price = 150 years of net income (i.e. after feeding the family) of av orphan ag farm. (See 4.3.3.3) [Based on Mazoyer and Roudart (2006) and Pimentel and Pimentel (2008)] | Same formula, based on average local food price over the last five years. | Tractors allow workers to farm a greater area but do not increase plot productivity. |
| Land price | Farmers may expand by purchasing unused adjacent plots. | Price/ha = the cost of 30 tonnes of cereal (Equivalent to the value of 30 years of average max production of orphan agriculture) (See 4.3.3.3). | Same formula, based on average local food price over the last five years. | Price chosen as this roughly represents the gross value produced on the land over the working life of an orphan farmer. |
| **Credit** |  |  |  |  |
| Annual interest rates | Interest rates on loans for fertilizer (short-term), animals and tractors (mid-term), and land (long-term)  (van der Ploeg, 2018). | Short-term (1 year): 20%,  mid-term (3 to 6 years): 15%, long-term (8 years): 10%.  Rates halved under ‘Entre’ and ‘Entre eroding’ policy. | Fixed, except under ‘Entre eroding’ policy where rates increase linearly over time, returning to their full values after 50 years. | Peasant farmers do not use credit. Rates based on Chandio and Jiang (2018), Chisasa and Makina (2012), Duniya and Adinah (2015), Malik and Nazli (1999). |

## Figures

Figure A show household actions and decision related to production, and Figures B and C show household actions and decisions related to the allocation of resources to consumption and production for peasant and entrepreneurial farmers, respectively.


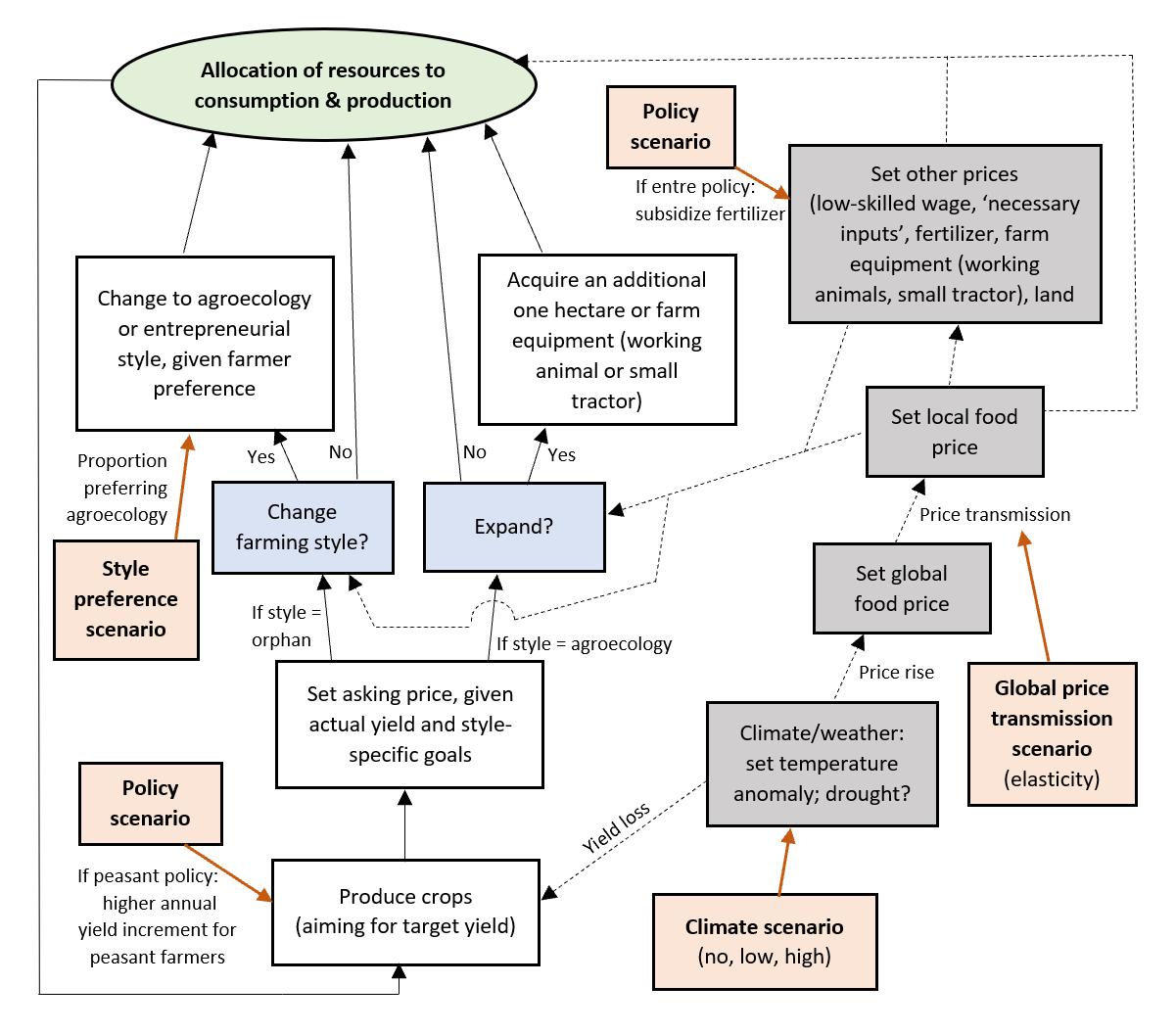


Figure A Household actions and decisions related to production. Scenarios are shown in orange; ‘observer’ (i.e. model controller) are shown in grey boxes and by dotted arrows; household decisions are shown in blue boxes; household actions are shown in white boxes and linked by solid arrows. Decisions related to the allocation of resources to consumption and production are shown in green and differ for peasant and entrepreneurial farmers (see Figures B and C)


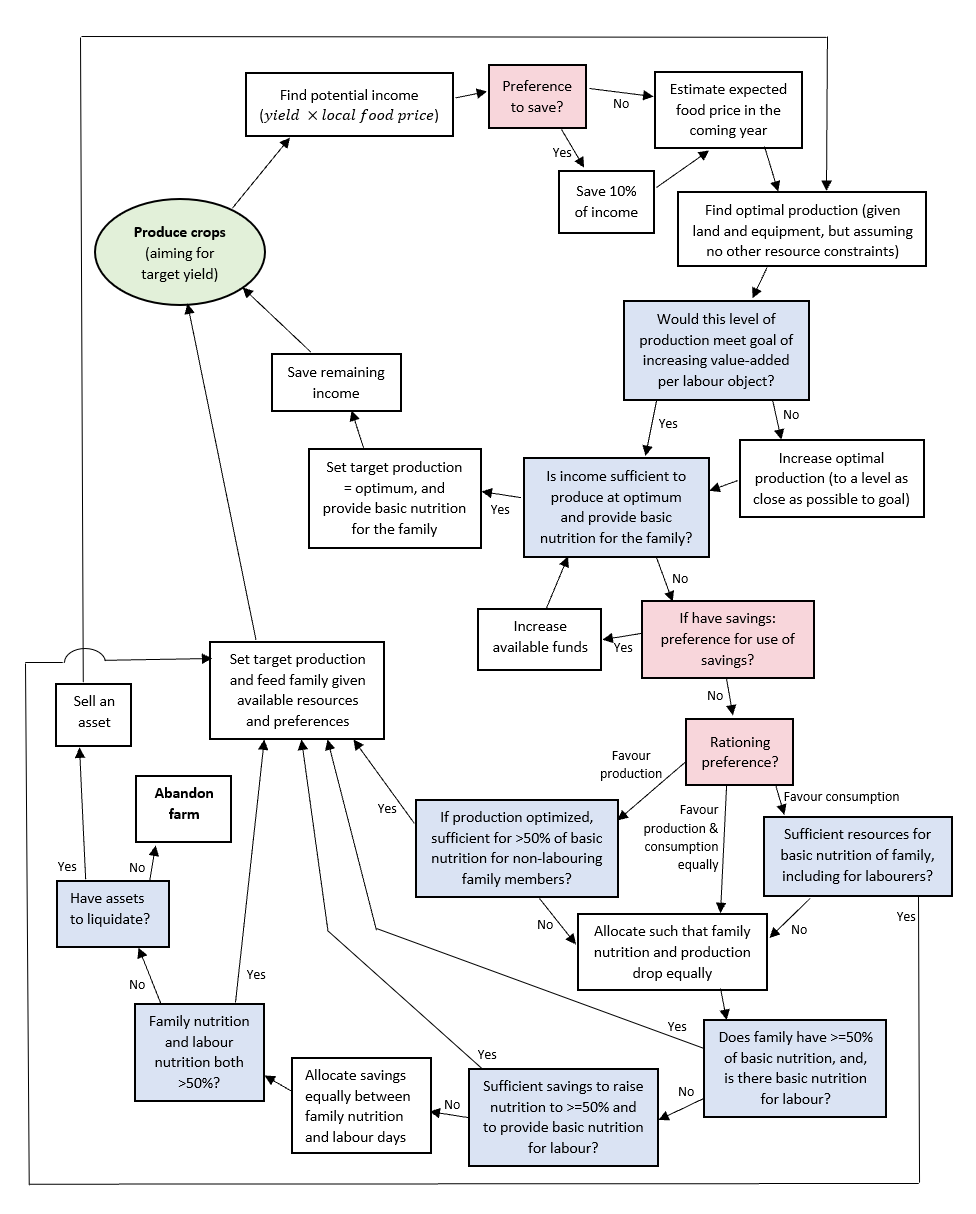


Figure B Peasant farmer actions and decisions related to the allocation of resources to consumption and production. Household decisions are shown in blue boxes except for decisions shaped by fixed preferences which are shown in red; household actions are shown in white boxes and linked by arrows. Decisions related to production are shown in green (see Figure A).


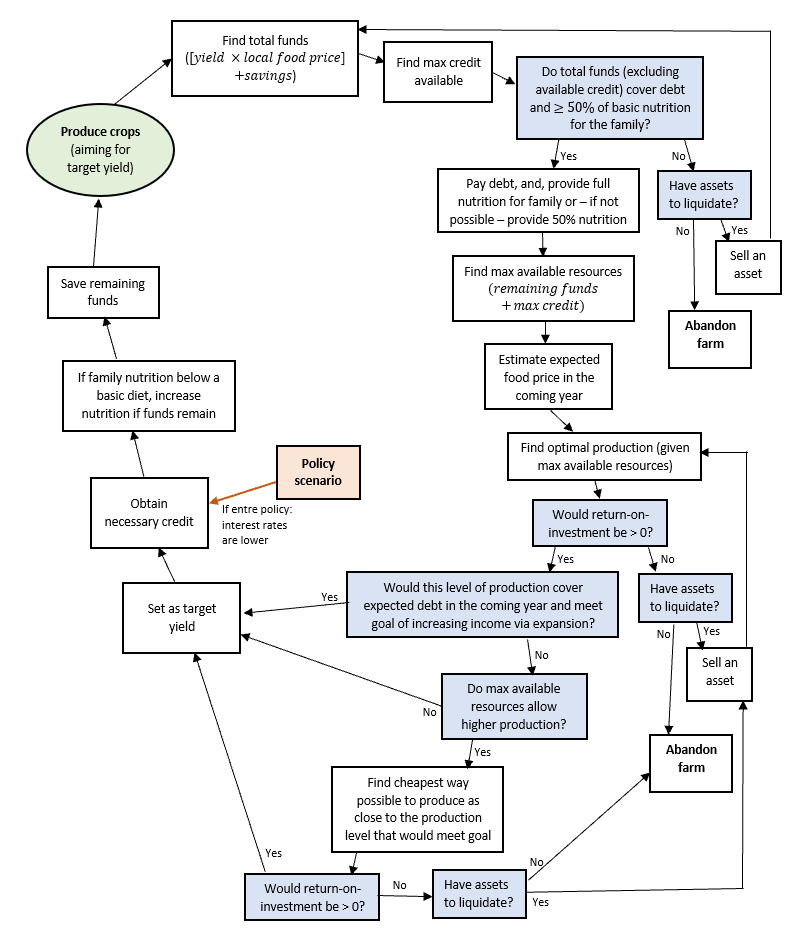


Figure C Entrepreneurial farmer actions and decisions related to the allocation of resources to consumption and production. Household decisions are shown in blue boxes; household actions are shown in white boxes and linked by arrows; scenarios are shown in orange. Decisions related to production are shown in green (see Figure A).

## Pseudo-code

In this section, relevant pseudo-code is shown for each of the six major model processes (see 1.3) and for the main model outputs (see 1.1). See also Tables A and B for how additional variables and parameters change over time.

### Set weather

#### Average yield losses if a drought occurs

Average yield losses if a drought occurs:

For high climate change, average yield loss = 15% to ~30%

= (15 + absolute value [normally distributed random floating point number

with (mean = 0, std dev = 7)]) / 100

For low climate change, average yield loss = 10% to ~25%

= (10 + absolute value [normally distributed random floating point number

with (mean = 0, std dev = 7)]) / 100

For no climate change, average yield loss = 7.5% to ~20%

= (7.5 + absolute value [normally distributed random floating point number

with (mean = 0, std dev = 6.5)]) / 100

### Produce crops

#### Household yield losses if a drought has occurred

For orphan and entrepreneurial households (note that ‘average yield losses’ are calculated in 4.3.1.1):

household drought yield loss = 80% to 120% of average losses for 90% of households

= average yield loss × (1 + ([normally distributed random

floating point number with (mean = 0, std dev = 10)] / 100))

For agroecology households, losses are calculated as for orphan and entrepreneurial households but then decreased by 20%.

#### Actual yield

Actual yield is target yield, reduced by warming-associated yield losses (Table A), drought-associated yield losses (4.3.2.1), and randomly adjusted (by up to ~$\pm$15%) to account for unmodelled factors.

actual yield = (target yield – (warming-associated yield losses + drought associated yield losses)) $\times$

((1 + normally distributed random floating point number with (mean = 0, std dev = 6.5) / 100)

#### Household asking price

Households finding their asking price in style-dependent manner. Asking prices are restricted such that they do not differ by more than $\pm$ 15% of expected price.

For peasant farmers:

yield for sale = what would remain of the yield if the family were provided with a basic diet and

sufficient for 90% of a maximum labour diet were set aside

= actual yield – (quantity required to provide a basic family diet +

quantity to provide 90% of a maximum labour diet)

desired income = the maximum of: either the cost of 90% of maximum necessary inputs, or, a net income of 105% of the

average net income over the previous five years^[[8]](#footnote-9)^

= max (90% of cost of maximum necessary inputs, 105% of five year average net income)

If yield for sale > 0 [asking price = (desired income) / (yield for sale)

if asking price > expected price $\times$ 1.15 [set asking price = expected price $\times$ 1.15]

if asking price < expected price $\times$ 0.85 [set asking price = expected price $\times$ 0.85]

]

If yield for sale < 0 [asking price = expected price $\times$ 1.15]^[[9]](#footnote-10)^

For entrepreneurial farmers:

yield for sale = actual yield

asking price = (target yield $\times$ expected price) / yield for sale)

if asking price > expected price $\times$ 1.15 [set asking price = expected price $\times$ 1.15]

if asking price < expected price $\times$ 0.85 [set asking price = expected price $\times$ 0.85]

#### Agroecology yield rise during transition period

Household yields rise gradually during the agroecology transition period, achieving the full agroecology yield multiple after the transition period:

During transition period, achieved yield multiple = (plot agroecology yield multiple) /

(agroecology transition period – plot years under agroecology)

### Set food prices and other prices

#### Global food price

Global food price is set such that it has a tendency to fall (fastest when no climate change; slowest under high climate change), oscillate, and rise in response to a drought.

Roughly linear tendency to fall (but may also rise):

For high climate change, working global food price = average fall of 1% per year

= global food price_(t-1)_ –

(global food price_(t-1)_ $\times$ 0.01) + normal distributed random floating point number with

(mean = 0, std dev = global food price_(t-1)_ $\times$ 0.025)

For low climate change, working global food price = average fall of 1.25% per year

= global food price_(t-1)_ –

(global food price_(t-1)_ $\times$ 0.0125) + normal distributed random floating point number with

(mean = 0, std dev = global food price_(t-1)_ $\times$ 0.0275)

For no climate change, working global food price = average fall of 1.5% per year

= global food price_(t-1)_ –

(global food price_(t-1)_ $\times$ 0.015) + normal distributed random floating point number with

(mean = 0, std dev = global food price_(t-1)_ $\times$ 0.03)

(where t is the current time step)

Check working global food price > 5c/kg (i.e. assume a price lower than this would be unreasonably low):

while (working global food price) $\leq$ 5c/kg [

set (working global food price) =

working global food price + (random floating point number $\geq$0 and $<$ 5)

]

Adjust for oscillation:

let amplitude = 0.3 and period = 10

oscillator = amplitude $\times$ $sin\left( \frac{2\pi}{period}\times time step \times\frac{180}{\pi} \right)$

working global food price = working global food price + (oscillation * 0.05)

Check working global food price >5c/kg, as per code above.

Adjust for drought:

For high climate change, drought price increase = 10% to 17.5%

= (random floating point number $\geq$ 10 and <17.5) / 100

For low climate change, drought price increase = 7.5% to 12.5%

= (random floating point number $\geq$ 7.5 and <12.5) / 100

For no climate change, drought price increase = 5% to 7.5%

= (random floating point number $\geq$ 5 and <7.5) / 100

global food price = working global food price $\times$ (1 + drought-price-increase)

#### Local food price

Local food price is the production-weighted average of household asking prices adjusted by global price transmission:

Production-weighted average of household asking prices =

$$\sum_{all households} \left[ household asking price \times\left( \frac{household yield for sale}{\sum_{all households} \left( yield for sale \right)} \right) \right]$$

Check production weighted price > 5c/kg (i.e. assume a price lower than this would be unreasonably low):

while (production-weighted average of household asking prices) $\leq$ 5c/kg [

set (production-weighted average of household asking price) =

production-weighted average of household asking price + (random floating point number $\geq$0 and $<$ 5)

]

Adjust for global price transmission:

% change global food price = (global food price_(t)_ – global food price_(t-1)_) / global food price_(t-1)_, where t is the current

time step

local food price = production-weighted average of household asking prices $\times$

((1 + % change in global food price) * global price transmission))

#### Prices of working animals, small tractors, and land

Prices are linked to average local food price over the previous 5 years.

price of working animals plus simple equipment = ~30 years of average income for an orphan farmer after feeding the

family a basic diet

= 30 years $\times$ (1000kg – 700kg) $\times$ local food price

$\approx$ ~10 years of average gross value product per ha for an orphan farmer

= 10 $\times$ 1000kg $\times$ five year average local food price

price of small tractor = ~150 years of average income for an orphan farmer after feeding the family a basic diet

= 150 years × (1000kg – 700kg) × local food price

≈ ~50 years of average gross value product per ha for an orphan farmer

= 50 × 1000kg × five year average local food price

price of 1ha of land = ~30 years of average gross value product per ha for an orphan farmer

= 30 × 1000kg × five year average local food price

### Consider farming style change

Orphan with preference for agroecology:

Change style if:

savings $\geq$ additional inputs required during transition period, which is:

[additional necessary inputs] + [additional labour diet], which is:

[(necessary inputs $\times$ (agroecology labour multiple – 1)] +

[(daily labour diet – daily basic diet) * annual labour days * local food price *

(agroecology labour multiple – 1)]

Orphan with preference for entrepreneurial:

Change style if:

savings + (income – family basic diet) $\geq$ low skilled wage

### Consider expansion

Orphan farmers cannot expand their farm beyond one hectare (the maximum area that can be managed by an orphan farmer using manual tools). Agroecology farmers may acquire up to 10 hectares plus two set of working animals (as this can be managed using family labour), and they only make purchased using their savings. Entrepreneurial farmers, who use wage labour, may acquire an unlimited number of hectares plus working animals or small tractors; they make purchases if they can pay at least half the cost using savings and acquire credit for the balance (mid-term credit for working animals and tractors; long-term credit for land).

Agroecology farmers:

IF all plots transitioned to agroecology [

AND IF have only 1 hectare of land AND savings > (price of working animals + one hectare of land) [

IF at least one plot adjacent to the farm is unoccupied, acquire the plot with the greatest yield potential

and one pair of working animals

]

ELSE IF have working animals that are able to farm more land than currently owned AND own < 10 hectares AND

savings > price of one hectare of land [

IF at least one plot adjacent to the farm is unoccupied, acquire the plot with the greatest yield potential

]

ELSE IF own 5 hectare of land AND own 1 pair of working animals AND savings > price of working animals [

acquire an additional pair of working animals

]

]

Entrepreneurial farmers:

IF own $\geq$ 1 tractor AND could farm more land than currently owned with these tractors AND

savings $\geq$ (price of one hectare / 2) [

IF at least one plot adjacent to the farm is unoccupied, acquire the plot with the greatest yield potential

]

ELSE IF (savings + current income + (price of owned working animals / 2)^[[10]](#footnote-11)^ $\geq$ (price of a small tractor / 2)) AND

mid-term debt = 0) [

acquire a tractor

]

ELSE IF own $\geq$ 1 pair of working animals AND could farm more land than currently owned with these working animals

AND savings $\geq$ (price of one hectare / 2) [

IF at least one plot adjacent to the farm is unoccupied, acquire the plot with the greatest yield potential

]

ELSE IF ((savings + current income) $\geq$ (price of working animals / 2)) AND no tractors owned AND mid-term debt = 0)[

acquire a pair of working animals

]

ELSE IF ((savings + current income) $\geq$ (low skilled-wage $\times$ farm size)^[[11]](#footnote-12)^) AND no working animals or tractors are

owned AND savings $\geq$ (price of one hectare / 2) [

IF at least one plot adjacent to the farm is unoccupied, acquire the plot with the greatest yield potential

]

### Allocate resources to consumption and production

#### Proportion of maximum yield achieved as a function of labour diet

Based on Strauss (1986).

Proportion of maximum yield achieved = *f*(proportion of maximum labour power)

= *f*(proportion of maximum labour diet consumed)

= -(proportion of maximum labour diet consumed)^2 +

2$\times$(proportion of maximum labour diet consumed)

#### Yield increases with fertilizer

With maximum fertilizer input (assumed to be 500kg/hectare) the yield multiple is assumed to be 10 (e.g. if yield on a hectare of land were 1 tonne then the application of 500kg of fertilizer would lead to a yield of 10 tonnes). Fertilizer use up to this maximum brings diminishing returns.

The table below shows the relation between fertilizer inputs and the achieved yield multiple. The top row (‘Prop’) is the proportion of maximum fertilizer used (e.g. 0.2 means 100kg of fertilizer was used) and the second row (‘Mult’) shows the associated yield multiple (e.g. if a proportion of 0.2 were used the yield multiple would be 3.6).

| **Prop** | .05 | .1 | .15 | .2 | .25 | .3 | .35 | .4 | .45 | .5 | .55 | .6 | .65 | .7 | .75 | .8 | .85 | .9 | .95 | 1 |
| --- | --- | --- | --- | --- | --- | --- | --- | --- | --- | --- | --- | --- | --- | --- | --- | --- | --- | --- | --- | --- |
| **Mult** | 1.1 | 1.9 | 2.8 | 3.6 | 4.4 | 5.1 | 5.8 | 6.4 | 7 | 7.5 | 8 | 8.4 | 8.8 | 9.1 | 9.4 | 9.6 | 9.8 | 9.9 | 9.98 | 10 |

#### Optimization

First, all households find their expected price in the coming year.

Expected price

Each household estimates their expected food price in the coming year, where expected price reflects the price farmers would aim to sell their yield for, given price trends and their style-specific goals. Thus, expected price is based on an initial estimate given price trends, which is then adjusted as follows to reflect style-specific goals: orphan farmers make no adjustment; entrepreneurial farmers aim to expand by capturing more of the market so reduce their expected price; agroecology farmers aim to increase autonomy by increasing returns-to-labour-object so they increase their expected price.

Step 1: find initial expected price

Initial expected price = current local food price +

(slope of local food price over previous five years $\times$ (random floating point number > -3.5 and <1.5)

Where, the random number represents unspecified factors when making judgements about price trends, but there is a general tendency to assume that the current trend will slow.

IF initial expected price $\leq$ 0 THEN set initial expected price = current price $\times$ 1.05

Step 2: adjust initial expected price given farming style

Orphan farmers:

Expected price = initial expected price

Entrepreneurial farmers:

Expected price = initial expected price $\times$ 0.98

Agroecology farmers:

Expected price = initial expected price $\times$ 1.15

Following this, households find their optimal production.

Find optimal production: peasant households

Step 1: Find production level that would maximise returns-to-labour, given land and equipment but assuming no other resource constraints. Maximising returns-to-labour is equivalent to optimizing production using standard economic methods (i.e. to maximise profit) (Debertin, 2012, Ellis, 1993) but without costing labour (van der Ploeg, 2013).

The total value product (TVP) curve is derived by modifying the curve for ‘proportion of maximum yield achieved’ given labour inputs (see 4.3.6.1) so that it accounts for household maximum achievable yield and expected food price.

TVP = [-(proportion of maximum labour diet consumed)^2 +

2×(proportion of maximum labour diet consumed)] $\times$ (expected price $\times$ maximum yield)

It is assumed that, for a given proportion of maximum labour power (which is equal to the proportion of a maximum labour diet consumed) to be productive, an equivalent proportion of maximum necessary inputs is required. Thus:

TVP = [-(proportion of maximum necessary inputs)^2 +

2×(proportion of maximum necessary inputs)] $\times$ (expected price $\times$ maximum yield)

[where the x-axis is proportion of maximum necessary inputs, and the y-axis is $]

The total factor cost (TFC) curve is derived such that it begins at the origin and slope is equal to the cost of necessary inputs that would allow maximum production. For the TFC curve: x-axis is proportion of maximum necessary inputs and y-axis is $.

TFC = (cost of necessary inputs that would allow maximum production) $\times$

(proportion of maximum necessary inputs)

Then, production is optimal when: $\frac{d(TVP)}{dx}=\frac{d(TFC)}{dx}$

Step 2: IF optimal production is at a level such that workers would not be consuming a basic diet THEN boost optimal production to a level where workers consume a basic diet.

IF optimal proportion of maximum necessary inputs $\times$ calories for maximum labour < calories in basic daily diet

THEN [optimal proportion of maximum necessary inputs =

calories in basic daily diet / calories for maximum labour power

= 2200 / 5100 = 0.43

]

Step 3: Assess whether optimal production would provide sufficient income to reproduce the farm: i.e. provide a family basic diet, and, allow the purchase of sufficient necessary inputs to enable workers consuming a basic labour diet to be fully productive. If not, attempt to increase production.

IF (gross income at optimal production – (family basic diet $\times$ expected food price)) <

((2200 / 5100) $\times$ cost of necessary inputs that would allow maximum production)

THEN [set optimal production to a level that provides required income

OR if not achievable, set optimal production = maximum achievable production

]

Step 4: Assess whether optimal production would provide sufficient income to achieve autonomy-related goal of increasing value added per labour object. This is assumed to be achieved if a household’s five year average net income is increased by 5%. If not, attempt to increase production.

IF (gross income at optimal production – (family basic diet $\times$ expected food price)) <

(five year average net income $\times$ 1.05)

THEN [set optimal production to a level that provides desired income

OR if not achievable, set optimal production = maximum achievable production

]

Step 5: see Figure A for subsequent household decisions on target production, rationing if necessary, and whether to abandon the farm.

Find optimal production: entrepreneurial households

Step 1: find production level that would maximise returns-on-investment given available resources including maximum available credit. Maximising returns-on-investment is based on the same curves used to optimize production in standard economic methods (i.e. to maximise profit) (Debertin, 2012, Ellis, 1993) but aims for a different target (van der Ploeg, 2013).

The total value product (TVP) curve has an x-axis for proportion of maximum labour used (which is equal to proportion of maximum necessary inputs used), a y-axis for proportion of maximum fertilizer inputs^[[12]](#footnote-13)^ used, and a z-axis for total value product in $. The total factor cost (TFC) curve has the same x- and y-axes but the z-axis is total factor cost in $. And for each combination of inputs, returns-on-investment (ROI) is given by:

$ROI= \frac{TVP-TFC}{TFC}$

The household sets optimal production at the level which, of the viable options (given available resources), maximises ROI.

$$viable ROIs=subset of ROIs that are achievable given resources$$

$$target ROI=max(viable ROIs)$$

$$optimal production=production level at which target ROI is achieved$$

Step 2: Check whether the expected return on investment is positive. If not, sell assets and re-optimize.

IF viable ROI < 0, THEN

[IF they have assets to sell [sell assets (see 4.3.6.4) and return to step 1]

ELSE [abandon the farm]

]

Step 3: Assess whether optimal production would allow household to cover expenses and achieve desired income in the coming year. If not, attempt to increase production.

Desired income is either (i) average net income per hectare over the previous 5 years if no additional land was acquired in the current time step, or (ii) if an additional hectare was acquired in this time step, 95% of average net income per hectare over the previous five year. The latter is because entrepreneurial farmers aim to increase income via expansion, where income is determined by margin per ha multiplied by scale (i.e. farm size) (van der Ploeg, 2018). Thus, if entrepreneurial farmers have expanded by acquiring an additional plot in the current time step, the margin per ha may fall while income rises.

IF no land acquired in the current time step

THEN [desired net income = five year average net income per hectare $\times$ farm size]

ELSE [desired net income = five year average net income per hectare $\times$ farm size $\times$ 0.95]

IF TVP at optimal < (debt obligations + basic diet for the household + desired income)

THEN [set target TVP = min ((debt obligations + basic diet for the household + desired income),

maximum viable TVP)

find lowest cost way of achieving target TVP

IF ROI for target TVP < 0 THEN [IF they have asset to sell [sell assets (see 4.3.6.4) and

return to step 1]

ELSE [abandon the farm]

For decisions and actions taken by peasant and entrepreneurial farmers following optimization see Figures B and C, respectively.

#### Sell assets

If households require additional funds to for the farm to survive they will sell equipment and land for half its value (i.e. assumed to require a quick sale by a struggling farm).

IF at least one small tractor owned [sell tractor for half its value AND sell any land that can no longer be farmed without

this equipment^[[13]](#footnote-14)^

]

Else IF at least one pair of working animals owned [sell pair of working animals for half their value AND sell any land

that can no longer be farmed without this equipment^[[14]](#footnote-15)^

]

### Model outputs

#### Total food produced

Total food produce by all households in a given year, quantified as kilograms of cereal equivalents, where 700kg feeds a family of 4 a basic diet (Mazoyer and Roudart, 2006).

$$T{otal food produced}_{t}= \sum_{all households} {household total food production}_{t}$$

(where $t$ is the current time step)

#### Local food price

See 4.3.3.2.

#### Income slope

Shown as average change in average net income over the previous 10 years for farmers practicing each style (units: $/year).

household net income = household gross income – all household expenses

five year average household net income = [sum of (household net income) over the previous five years] / 5

mean of five year average household net income for all household practicing a given style =

[sum of (five year average household net income for households practicing a given style)] /

(number of households practicing that style)

For finding average income slope for all households practicing a given style , let x-axis = time step and y-axis = mean of five

year average household net income for households practicing a given style, then:

style-specific income slope = $\frac{dy}{dx}$ , over the previous 10 years

#### Orphan nutrition

Average nutrition across all orphan households, as the proportion of a basic diet being consumed.

Household nutrition = (kg of cereal equivalents consumed by a household) / 700kg, where 700kg provide a basic diet.

Orphan nutrition = mean (household nutrition) of orphan households

#### Farm labour

For orphan agriculture, max production on 1ha with manual tools requires 150 ten hour labour days/year (Based on Pimentel and Pimentel, 2008). Thus, a full-time worker is assumed to be working 150 ten hour labour days/year, regardless of the area they farm (which is dependent on their equipment). Full-time workers include both family labour on peasant farms and wage labour on entrepreneurial farms.

Farm labour = sum of (full-time equivalent workers) on all farms

#### Income Gini

The Gini coefficient is calculated using standard methods (e.g. Milanovic, 2005), based on five year average household net incomes. A value of 0 indicates perfect inequality and a value of 1 indicates maximum inequality.

#### Mean net farm income

Mean net farm income is the average of the five year average net income of all farming households.

household net income = household gross income – all household expenses

five year average household net income = [sum of (household net income) over the previous five years] / 5

mean of five year average household net income = [sum of (five year average household net income)] /

(number of households)

#### Real land productivity

Real land productivity is net income per hectare adjusted for the proportion of value that was added on the farm (‘endogeneity’), calculated (based on Petersen and Silveira, 2017) as follows.

For each household:

$real land productivity [{\$}/{ha}]= \frac{household net income in a given year [\$]}{farm size [ha]} \times endogeneity$ , where:

$endogeneity= \frac{value added on the farm [\$]}{value of total farm production [\$]}$

$= \frac{value of total farm production-\left( purchased inputs excluding labour \right) [\$]}{value of total farm production [\$]}$ , where:

$value of total farm production=actual farm \times local food price$

# REFERENCES

BRUINSMA, J. (ed.) 2003. *World Agriculture: Towards 2015/2030. An FAO Perspective,* London: Earthscan.

BUTTEL, F. H. 2000. Ending Hunger in Developing Countries. *Contemporary Sociology,* 29**,** 13-27.

CHANDIO, A. A. & JIANG, Y. 2018. Determinants of Credit Constraints: Evidence from Sindh, Pakistan. *Emerging Markets Finance and Trade,* 54**,** 3401-3410.

CHISASA, J. & MAKINA, D. 2012. Trends in credit to smallholder farmers in South Africa. *The International Business & Economics Research Journal,* 11**,** 771-784.

DE LA O CAMPOS, A. P., VILLANI, C., DAVIS, B. & TAKAGI, M. 2018. Ending extreme poverty in rural areas – Sustaining livelihoods to leave no one behind. Rome: FAO.

DEBERTIN, D. L. 2012. *Agricultural Production Economics,* Lexington, Kentucky, David L Derbetin (Amazon Createspace).

DUNIYA, K. & ADINAH, I. 2015. Probit analysis of cotton farmers' accessibility to credit in northen guinea savannah of Nigeria. *Asian J Agric Ext Econ Sociol,* 4**,** 296-301.

ELLIS, F. 1993. *Peasant Economics,* Cambridge, Cambridge University Press.

HLPE 2019. Agroecological and other innovative approaches for sustainable agriculture and food systems that enhance food security and nutrition. A report by the High Level Panel of Experts on Food Security and Nutrition of the Committee on World Food Security. Rome: HLPE.

HOLT-GIMENEZ, E. 2019. *Cna we fed the world without detroying it?,* Cambridge, Polity.

HOLT-GIMENEZ, E. & PATEL, R. 2009. *Food Rebellions! Crisis and the Hunger for Justice,* Oxford, Pambazuka Press.

IFAD. Conference on New Directions for Smallholder Agriculture, 24-25 January 2011, Rome, IFAD HQ: Proceedings of the Conference. New Directions for Smallholder Agriculture, 2011 Rome. IFAD.

ILO 2016. Global Wage Report 2016/17: Wage inequality in the workplace. Geneva: International Labour Organization.

KNUTTI, R. & SEDLÁČEK, J. 2012. Robustness and uncertainties in the new CMIP5 climate model projections. *Nature Climate Change,* 3**,** 369.

LA VIA CAMPESINA. 2019. *La Via Campesina: International Peasants Movement* [Online]. Available: http://www.viacampesina.org/en/ [Accessed October 22 2019].

LLOYD, S. J., KOVATS, R. S. & CHALABI, Z. 2011. Climate Change, Crop Yields, and Undernutrition: Development of a Model to Quantify the Impact of Climate Scenarios on Child Undernutrition. *Environmental Health Perspectives,* 119**,** 1817-1823.

MALIK, S. J. & NAZLI, H. 1999. Rural Poverty and Credit Use: Evidence from Pakistan. *The Pakistan Development Review,* 38**,** 699-716.

MAZOYER, M. & ROUDART, L. 2006. *A History of World Agriculture: from the Neolithic Age to the Current Crisis,* London, Earthscan.

MILANOVIC, B. 2005. *Worlds Apart: Measuring International and Global Inequality,* Princeton, Princeton University Press.

MOORE, F. C., BALDOS, U., HERTEL, T. & DIAZ, D. 2017. New science of climate change impacts on agriculture implies higher social cost of carbon. *Nature Communications,* 8**,** 1607.

MOORE LAPPE, F. & COLLINS, J. 2015. *World Hunger: Ten Myths,* New York, Food First.

MOSS, R. H., EDMONDS, J. A., HIBBARD, K. A., MANNING, M. R., ROSE, S. K., VAN VUUREN, D. P., CARTER, T. R., EMORI, S., KAINUMA, M., KRAM, T., MEEHL, G. A., MITCHELL, J. F. B., NAKICENOVIC, N., RIAHI, K., SMITH, S. J., STOUFFER, R. J., THOMSON, A. M., WEYANT, J. P. & WILBANKS, T. J. 2010. The next generation of scenarios for climate change research and assessment. *Nature,* 463**,** 747-756.

PETERSEN, P. F. & SILVEIRA, L. M. 2017. Agroecology, Public Policies and Labor-Driven Intensification: Alternative Development Trajectories in the Brazilian Semi-Arid Region. *Sustainability,* 9.

PIMENTEL, D. & PIMENTEL, M. H. 2008. *Food, Energy, and Society,* Boca Raton, CRC Press.

PRETTY, J., BENTON, T. G., BHARUCHA, Z. P., DICKS, L. V., FLORA, C. B., GODFRAY, H. C. J., GOULSON, D., HARTLEY, S., LAMPKIN, N., MORRIS, C., PIERZYNSKI, G., PRASAD, P. V. V., REGANOLD, J., ROCKSTRÖM, J., SMITH, P., THORNE, P. & WRATTEN, S. 2018. Global assessment of agricultural system redesign for sustainable intensification. *Nature Sustainability,* 1**,** 441-446.

PRETTY, J. N., MORISON, J. I. L. & HINE, R. E. 2003. Reducing food poverty by increasing agricultural sustainability in developing countries. *Agriculture, Ecosystems & Environment,* 95**,** 217-234.

ROSER, M. & RITCHIE, H. 2019. *Fertilizer and Pesticides* [Online]. Available: https://ourworldindata.org/fertilizer-and-pesticides [Accessed October 11 2019].

ROSSET, P. M. & ALTIERI, M. A. 2017. *Agroecology: Science and Politics,* Rugby, Practical Action Publishing.

ROSSETT, P. 2006. *Food is Different: Why We Must Get the WTO Out of Agriculture,* London, Zed Books.

STRAUSS, J. 1986. Does Better Nutrition Raise Farm Productivity? *Journal of Political Economy,* 94**,** 297-320.

VAN DER PLOEG, J. D. 2013. *Peasants and the Art of Farming: A Chayanovian Manifesto,* Halifax, Fernwood Publications.

VAN DER PLOEG, J. D. 2016. Perspective: How Peasants Read Their Farm. *Farming Matters,* 32.

VAN DER PLOEG, J. D. 2017. The importance of peasant agriculture: a forgotten truth. Wageningen: Wageningen University.

VAN DER PLOEG, J. D. 2018. *The New Peasantries: Rural Development in Times of Globalization,* Oxon, Routledge.

VAN DER VELDE, M., SEE, L., YOU, L., BALKOVIČ, J., FRITZ, S., KHABAROV, N., OBERSTEINER, M. & WOOD, S. 2013. Affordable Nutrient Solutions for Improved Food Security as Evidenced by Crop Trials. *PLOS ONE,* 8**,** e60075.

WAGE INDICATOR FOUNDATION. 2019. *Wage Indicator* [Online]. University of Amsterdam. Available: https://wageindicator.org/ [Accessed October 10 2019].

WILENSKY, U. 1999. Netlogo. Evansto, IL.: Centre for Connected Learning and Computer-Based Modeling, Northwestern University

WORLD BANK. 2019. *World Bank Development Indicators* [Online]. Available: http://data.worldbank.org/data-catalog/world-development-indicators [Accessed October 10 2019].

YAMANO, T. & ARAI, A. 2011. Fertilizer Policies, Price, and Application in East Africa. *In:* YAMANO, T., OTSUKA, K. & PLACE, F. (eds.) *Emerging Development of Agriculture in East Africa: Markets, Soil, and Innovations.* Dordrecht: Springer.

ZHAO, C., LIU, B., PIAO, S., WANG, X., LOBELL, D. B., HUANG, Y., HUANG, M., YAO, Y., BASSU, S., CIAIS, P., DURAND, J.-L., ELLIOTT, J., EWERT, F., JANSSENS, I. A., LI, T., LIN, E., LIU, Q., MARTRE, P., MÜLLER, C., PENG, S., PEÑUELAS, J., RUANE, A. C., WALLACH, D., WANG, T., WU, D., LIU, Z., ZHU, Y., ZHU, Z. & ASSENG, S. 2017. Temperature increase reduces global yields of major crops in four independent estimates. *Proceedings of the National Academy of Sciences,* 114**,** 9326-9331.

1. Note that global corporate agriculture is not strictly an agent in that it does not take actions based on decisions; however, it is characterized as an agent here as it represents a farming entity and future iterations of the model will make it increasingly agent-like. [↑](#footnote-ref-2)
2. This is standard terminology in NetLogo, the platform in which the model was coded. [↑](#footnote-ref-3)
3. In the accompanying paper, the transition period is set to 3 years in all model runs. [↑](#footnote-ref-4)
4. Note these steps are included in ‘produce crops’ as the labour-intensive production process on peasant farms leads to both ongoing yield increments and agroecology transitions. [↑](#footnote-ref-5)
5. Interest rates remain constant over time in other agricultural policy scenarios. [↑](#footnote-ref-6)
6. In the real-world, this is not a binary distinction and farmers may be more peasant- or entrepreneurial-like. In this iteration of the model, however, as we are attempting to take a first look at the implications of farming style for hunger and health under climate change, we treat the distinction as being binary. [↑](#footnote-ref-7)
7. That is, guaranteed except if faced with unforeseen circumstances such as significant crop losses. [↑](#footnote-ref-8)
8. The former is the quantity of necessary inputs required for 90% of maximum labour to be fully productive; the latter represents an increase on value added per labour object (i.e. a key goal of peasant farmers). [↑](#footnote-ref-9)
9. If a household would have nothing for sale after meeting a basic diet and 90% of a labour diet they will boost they asking price by as much as they think is viable: this is assumed to be 15% above their expected price. Households will sell at least some of their yield as they require at least some necessary inputs if they are to produce crops. [↑](#footnote-ref-10)
10. If moving for using working animals to using tractors, farmers will sell their working animals at half their initial value. [↑](#footnote-ref-11)
11. This is the cost of wages to farm all the plots currently owned; i.e. using manual tools, each working can manage 1 hectare). [↑](#footnote-ref-12)
12. ‘Fertilizer’ stands in for all purchased inputs (e.g. herbicides, pesticides). [↑](#footnote-ref-13)
13. i.e. each small tractor allows one worker to farm 16 hectares. [↑](#footnote-ref-14)
14. i.e. each pair of working animals allows one worker to farm 5 hectares. Note that after selling the last pair of working animals a household will have only 1 hectare of land. [↑](#footnote-ref-15)
